# The relationship between leaf area growth and biomass accumulation in *Arabidopsis thaliana*

**DOI:** 10.3389/fpls.2015.00167

**Published:** 2015-04-09

**Authors:** Sarathi M. Weraduwage, Jin Chen, Fransisca C. Anozie, Alejandro Morales, Sean E. Weise, Thomas D. Sharkey

**Affiliations:** ^1^Department of Biochemistry and Molecular Biology, Michigan State UniversityEast Lansing, MI, USA; ^2^Department of Energy Plant Research Laboratory, Michigan State UniversityEast Lansing, MI, USA; ^3^Department of Computer Science and Engineering, Michigan State UniversityEast Lansing, MI, USA; ^4^Centre for Crop Systems Analysis, Wageningen UniversityWageningen, Netherlands

**Keywords:** carbon partitioning, photosynthesis, leaf area, leaf thickening, growth, specific leaf area

## Abstract

Leaf area growth determines the light interception capacity of a crop and is often used as a surrogate for plant growth in high-throughput phenotyping systems. The relationship between leaf area growth and growth in terms of mass will depend on how carbon is partitioned among new leaf area, leaf mass, root mass, reproduction, and respiration. A model of leaf area growth in terms of photosynthetic rate and carbon partitioning to different plant organs was developed and tested with *Arabidopsis thaliana* L. Heynh. ecotype Columbia (Col-0) and a mutant line, *gigantea*-2 (*gi*-2), which develops very large rosettes. Data obtained from growth analysis and gas exchange measurements was used to train a genetic programming algorithm to parameterize and test the above model. The relationship between leaf area and plant biomass was found to be non-linear and variable depending on carbon partitioning. The model output was sensitive to the rate of photosynthesis but more sensitive to the amount of carbon partitioned to growing thicker leaves. The large rosette size of *gi*-2 relative to that of Col-0 resulted from relatively small differences in partitioning to new leaf area vs. leaf thickness.

## Introduction

Leaf area growth determines light interception and is an important parameter in determining plant productivity (Gifford et al., 1984; Koester et al., 2014). In addition, high-throughput phenotyping of plants often relies on optical methods in which leaf area growth is compared with estimates of photosynthesis derived from fluorescence signals. Optical measurements such as leaf area are well suited for high throughput screening for plants with altered photosynthetic rates because they are non-destructive and cost-effective (Golzarian et al., 2011; Zhang et al., 2012; Tessmer et al., 2013). Introduction of a High-Throughput Plant Growth Analysis Model that allows determination of the total leaf area based on projected leaf area (Tessmer et al., 2013), estimation of shoot biomass from high throughput plant images (Golzarian et al., 2011) and the use of high-throughput optical phenotyping to reveal genetic variation between plants (Zhang et al., 2012) have been attempted. In order to use leaf area as a tool to screen for plants with enhanced biomass or mass-based relative growth rates (RGR_M_) (see Table 1 for a list of abbreviations), it is important to understand the relationship between leaf area growth and accumulation of biomass.

**Table 1:** **Descriptions and symbols of key input and output state variables and parameters of the *Arabidopsis* Leaf Area Growth Model**.

**Description**		**Symbol**	**Units**	**Input values**
**STATE VARIABLES**				
A	Net rate of photosynthesis per leaf area	*A*	μmol m^−2^ s^−1^	Measured
	Net rate of photosynthesis per plant	*A*_P_	μmol plant^−1^ s^−1^	
S	Rate of starch synthesis per plant	*S*_P_	μmol plant^−1^ h^−1^	
	Rate of starch degradation per plant	*S*^D^_P_	μmol plant^−1^ h^−1^	
s	Surface area of leaf	s	m^2^ plant^−1^	
	Initial leaf area		m^2^ plant^−1^	5.0 × 10^−6^
M	Mass of leaf	M_L_	g plant^−1^	
	Initial leaf mass		g plant^−1^	1.8 × 10^−5^
	Mass of inflorescence	M_i_	g plant^−1^	
	Mass of root	M_r_	g plant^−1^	
	Mass of whole plant	M_P_	g plant^−1^	
R	Leaf maintenance respiration	R^m^_L_	μmol m^−2^ s^−1^	3.5 × 10^−1^
	Inflorescence maintenance respiration	R^m^_i_	μmol plant^−1^ h^−1^	
	Root maintenance respiration	R^m^_r_	μmol plant^−1^ h^−1^	
	Growth respiration of leaf growth	R^g^_L_	g plant^−1^ h^−1^	
	Inflorescence growth respiration	R^g^_i_	g plant^−1^ h^−1^	
	Root growth respiration	R^g^_r_	g plant^−1^ h^−1^	
	Plant growth respiration	R^g^_P_	g plant^−1^ h^−1^	
E	Root exudation	E	μmol plant^−1^ h^−1^	1.0 × 10^−3^
**INPUT AND OUTPUT PARAMETERS**				
	Ratio of mobilized reserves to seed weight			2.5 × 10^−1^
	Ratio of starch synthesis to photosynthesis	σ		6.0 × 10^−1^
ŕ	Inflorescence maintenance respiratory coefficient	ŕ*^m^_i_*	μmol g^−1^ s^−1^	7.0 × 10^−3^
	Root maintenance respiratory coefficient	ŕ^m^_r_	μmol g^−1^ s^−1^	1.6 × 10^−2^
	Growth respiratory coefficient of leaves	ŕ^g^_L_	g g^−1^	1.04 × 10^−1^
	Growth respiratory coefficient of inflorescence	ŕ^g^_i_	g g^−1^	1.7 × 10^−1^
	Growth respiratory coefficient of roots	ŕ*^g^_r_*	g g^−1^	1.3 × 10^−1^
RGR	Area-based relative growth rate	*RGR_s_*	m^2^ m^−2^ day^−1^	
	Mass-based relative growth rate	*RGR*_M_	g kg^−1^ day^−1^	

*Abbreviations and units for key state variables and parameters and specific input values are given. Letters in subscripts represent plant_(P)_ or organs: leaf_(L)_, inflorescence_(i)_, and root_(r)_. Letters in superscript provides a description: degradation^(D)^, thickening^(t)^, maintenance^(m)^, and growth^(g)^. Letters in regular script represents model state variables: photosynthesis (A), starch synthesis (S), surface area (s), mass (M), respiration (R), exudation (E), and parameters: starch partitioning coefficient (σ), respiratory coefficients (ŕ), and relative growth rate (RGR)*.

Carbon (C) that is fixed in photosynthesis is partitioned, first between sucrose synthesis for immediate use and export, and starch synthesis to supply reduced C at night. The reduced C supplied to the plant supports maintenance respiration with the remaining C available for growth. Reduced C used for growth is partially consumed in growth respiration which provides energy to convert the remaining C to new biomass. C partitioning to drive leaf thickening, leaf area growth, as well as to drive growth of other organs may depend on the developmental phase of the plant. Depending on C partitioning, leaf area may or may not be a good indicator of total plant biomass. RGR_M_ of a plant or a specific plant organ depends on the partitioning of photosynthetic C between new leaf and root growth, respiration, exudation, and reproduction. While area-based photosynthesis has been shown to only weakly correlate with RGR_M_, differences in RGR_M_ between plants is very sensitive to variations in parameters related to leaf area (s) including leaf area per unit leaf mass (M_L_) or specific leaf area (s/M_L_ or SLA) and the proportion of total plant mass invested in leaves, or leaf mass ratio (M_L_/M_P_) (Table 1) (Shipley, 2002; Lambers et al., 2008; Poorter et al., 2009). Growth in leaf mass can result from an increase in area or thickness; Total leaf mass growth is the sum of mass increase for leaf area growth and leaf thickening.

Many growth models have been developed to simulate growth and development of a variety of plants *in silico*, including that of *Saccharum* (Marin and Jones, 2014), *Brassica* (Grossman et al., 2011), and *Arabidopsis* (Mündermann et al., 2005; Rasse and Tocquin, 2006). Some models have been designed to simulate growth of a specific plant organ such as the root (Bidel et al., 2000), leaves (Asl et al., 2011; Tessmer et al., 2013), and inflorescence (Letort et al., 2006). At present, several *Arabidopsis* growth models which simulate shoot development (Mündermann et al., 2005), plant growth (Rasse and Tocquin, 2006), leaf epidermal cell division and expansion (Asl et al., 2011), and determination of total leaf area from projected leaf area (Tessmer et al., 2013) exist. Rasse and Tocquin (2006) investigated the effect of transient starch production on plant growth. However, the relationship between leaf area growth and biomass, the effect of variation in C partitioning between leaf area growth and thickening, and its impact on biomass accumulation have not been treated by these models.

The *Arabidopsis* Leaf Area Growth Model was developed to follow the flow of storage C and photosynthetic C from seed germination to leaf senescence. The model simulates the use of assimilated C in respiratory processes and the partitioning of the remaining C or net assimilated C to leaf area growth and leaf thickening, root growth, and reproduction or stem/inflorescence growth. The model was tested using data obtained from *Arabidopsis thaliana* L. Heynh. ecotype Columbia (Col-0) wild type and a mutant line, *gigantea*-2 (*gi*-2) because gigantea plants have very large rosettes.

## Theory

**Figure 1:**
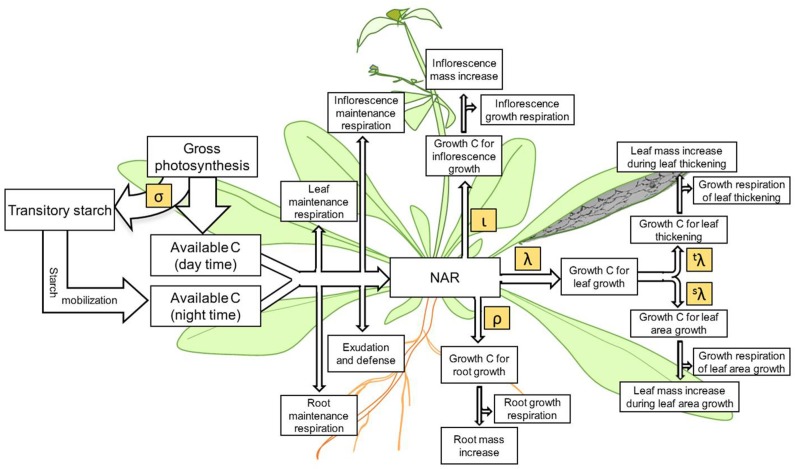
**The underlying scheme of C flow represented in the *Arabidopsis* Leaf Area Growth Model**. Processes of C assimilation, consumption, partitioning and accumulation accounted for in the present model is highlighted. During the day, while a portion of assimilated carbon is directly used to support growth and maintenance processes in the plant a significant portion of assimilated C is partitioned to starch, which is later degraded and mobilized to support growth and maintenance processes during the night. The net assimilation rate or NAR is the net amount of assimilated C remaining for plant growth after consumption in maintenance respiration, exudation, and defense processes. Some of the C partitioned to leaf, inflorescence/stem/seeds, and roots is used in growth respiration to produce the energy to transform the remaining C to new biomass. Carbon allocated to leaf growth is partitioned to increase leaf area (s) and to increase leaf thickness (t). The symbols σ, ι, ρ, λ, ^s^λ, and ^t^λ represent the partition coefficients of the corresponding processes.

The Net Assimilation Rate (NAR) (Lambers et al., 2008) provides C for growth. This is CO_2_ assimilation, as typically measured by gas exchange, minus respiration. We define growth to be the use of reduced C to make new leaf, root, or inflorescence tissue but do not include starch synthesis as growth. Therefore, growth can be positive at night, if starch is converted to new tissue. NAR is thus photosynthesis minus starch synthesis minus maintenance respiration (Figure 1).

Leaf area growth depends on partitioning at several levels. Partitioning of photosynthate between starch and sucrose is necessary to ensure sufficient C reserves through the night but as long as that criterion is satisfied a range of partitioning can be tolerated (Stitt and Zeeman, 2012). In the model, C for growth is partitioned among the leaves, roots, and inflorescences plus stems. Within leaves, growth C can be partitioned between area growth and leaf thickening. Growth C can be partitioned between expansive growth (mostly water uptake) and addition of new mass to the tissue. Our model tracks only mass; over a long period of time water uptake and mass deposition converge so that the water content of plants is relatively constant.

The NAR during the day is the whole plant assimilation minus starch synthesis and maintenance respiration. The partitioning of photosynthetic C to starch is denoted by σ and other abbreviations are given in Table 1.

(1) $$NAR_{Day}=A\;\cdot \;s\;\cdot \;\left(1-\sigma \right)-R_{i}^{m}-R_{r}^{m}-E$$

Starch available at night (Σ) is the sum of all the starch made during the day:

(2) $$\sum \;=\;\sum_{Lights\;on}^{Lights\;off}A\;\cdot \;s\;\cdot \;\sigma$$

Therefore, NAR at night is:

(3) $$NAR_{Night}=\frac{\sum }{Night\;length}-R_{L}^{m}-R_{i}^{m}-R_{r}^{m}-E$$

C available for growth is partitioned among leaves, inflorescence plus stem, and roots as follows:

(4) $$Growth\text{ C}=\frac{NAR\;\cdot \;\left(\lambda +\iota +\rho \right)}{C}$$

The partitioning coefficients of photosynthetic C available for growth to leaves, inflorescence/stem, and roots are denoted by λ, ι, and ρ, respectively, with each partition coefficient being the fraction of available C used by the leaf, inflorescence/stem and root. In the model these three partitioning coefficients sum to 1. Since NAR is in units of mole/plant, the concentration of carbon, *C*, is used to convert from mole/plant of carbon to g/plant of plant biomass. Some of the C partitioned to growth is consumed in growth respiration. Moreover, the C partitioned to leaves can cause leaves to expand in area or to become thicker. The partitioning to leaves can be broken out as follows:

(5) $$NAR\;\cdot \;\lambda =\frac{\Delta^{s}M_{L}}{1{-}^{s}R^{g}{}_{L}}+\frac{\Delta^{t}M_{L}}{1{-}^{t}R^{g}{}_{L}}$$

In words, the proportion of fixed C partitioned to leaves can increase leaf area or leaf thickness or both and the amount of mass added to the leaf is reduced by the respiratory processes needed to convert the fixed C into tissue. The increase in leaf area for a given increase in leaf mass will depend on the Specific Leaf Area (SLA) which is a measure of thickness of the leaf. That is, more fixed C is required to increase the area of thick leaves than of thin leaves.

(6) $$SLA=\frac{s}{M_{L}}$$

Therefore, leaf area growth will be linearly related to SLA.

(7) $$\Delta s=\Delta^{s}M_{L}\;\cdot \;\frac{s}{M_{L}}$$

The full equation for leaf area growth during the day can be derived from the above equations.

(8) $$\begin{array}{l} \Delta s=\frac{s}{M_{L}}\cdot \left(1{-}^{s}R^{g}{}_{L}\right)\;\cdot \;(\left(\frac{A\;\cdot \;s\;\cdot \;\left(1-\sigma \right)-R_{i}^{m}-R_{r}^{m}-E}{C}\right)\text{​}\\ -\frac{\Delta^{t}M_{L}}{1{-}^{t}R^{g}{}_{L}}-\text{ ​​}\frac{\Delta M_{i}}{1-R_{i}^{g}}-\text{​ ​}\frac{\Delta M_{r}}{1-R_{r}^{g}} \end{array}$$

Equation (8) shows that increases in leaf area will be linearly related to SLA but the relationship between leaf area growth and photosynthesis per unit leaf area (as commonly measured) is less direct. This allows for non-intuitive relationships between growth, especially leaf area growth, and area-based photosynthetic rate. Since growth can occur at night as well as day (Schurr et al., 2006), the model calculates growth at night as:

(9) $$\Delta s=\frac{s}{M_{L}}\;\cdot \;\left(1{-}^{s}R^{g}{}_{L}\right)\;\cdot \;\left(\left(\frac{NAR_{Night}}{C}\right)\;\cdot \;\left(1{-}^{t}\text{​​}\lambda -\iota -\rho \right)\right)$$

## Materials and methods

### Development of the *Arabidopsis* Leaf Area Growth Model

This model was designed and developed to simulate plant growth of *Arabidopsis* with special emphasis on C partitioning to leaf area growth and leaf thickening. The model simulates 90 days of plant growth using a fixed time step of 1 h. The modeled lifespan was divided into three main growth stages as follows: (1) heterotrophic phase [1–4 days after seeding (DAS)], (2) vegetative phase (5–66 DAS), and (3) reproductive phase (67–90 DAS). The durations of the growth phases were derived from experimental data obtained from Col-0 plants grown under an 8 h photoperiod. The first day on which the inflorescence was visible for Col-0 was taken as the 1st day of the reproductive phase. The 1st 2/3 and the last 1/3 of the vegetative phase were defined as the early vegetative phase (5–45 DAS) and the late vegetative phase (46–66 DAS), respectively. The model takes into consideration the fact that during the heterotrophic phase all energy requiring pre- and post-germination processes are dependent on stored C reserves (Kircher and Schopfer, 2012).

The model was also based on earlier findings that stored C reserves are depleted 4–5 days after germination after which cotyledons assume the role of the primary photosynthetic organ and that mass accumulation and true leaf growth occurs only after photosynthesis has begun (Kircher and Schopfer, 2012). Accordingly, as 90% of seed weight includes seed storage material and the cotyledons of the embryo is the major component of the mature *Arabidopsis* seed, the initial weight of the cotyledons was assumed to be equal to 90% of the weight of the seed. The initial leaf area was assumed to be that of the expanded cotyledons. The model was also designed to initiate root growth only after the cotyledons begin to supply photosynthetic C (Kircher and Schopfer, 2012).

The model first calculates the net amount of C fixed by photosynthesis per plant by multiplying the area-based photosynthetic rate by the projected leaf area. The total leaf area minus projected leaf area was considered to be leaves shaded by upper leaves and were assumed to photosynthesize at 10% of the rate of the exposed leaves. Next, the amount of C available for growth after subtracting C used in maintenance respiration and exudation (net assimilation rate, NAR) is computed (Figure 1). Because the typical measurement of A is net CO_2_ uptake, R*^m^_L_* and R*^g^_L_* is not included in daytime calculations but is included in nighttime calculations. If photosynthesis is estimated from fluorescence, R*^m^_L_* and R*^g^_L_* should be included in daytime calculations.

It was assumed that respiration in all leaves, inflorescence/stems, and roots consists of two major components: growth and maintenance respiration (Penning De Vries et al., 1974; Amthor, 1984). In the model, growth respiration is proportional to the growth rate of a plant or organ (Penning De Vries et al., 1974; Amthor, 1984; Thomas et al., 1993; Lambers et al., 2008). Thus, growth coefficients are defined as the amount of C respired (μmol or g) per unit increase in mass (g^−1^) or area (m^−2^) of the plant or specific organ (Mariko, 1988; Thomas et al., 1993). The value of a growth coefficient depends on the average biochemical composition of the plant or specific organ. Maintenance respiration is proportional to the dry mass of a plant or organ in the model (Penning De Vries et al., 1974; Amthor, 1984; Thomas et al., 1993; Lambers et al., 2008). Therefore, a maintenance coefficient (μmol g^−1^ s^−1^) is defined as the amount of C respired to maintain the existing mass or area of the plant or specific organ (Mariko, 1988; Thomas et al., 1993). The model is also based on the assumption that growth respiration coefficients of leaf thickening and leaf area growth are the same during all growth phases.

It was considered that starch/sucrose partitioning would be optimum if there were sufficient C at night to match the growth rate during the day. In this way resources such as ribosomes would be used at the same rate over the 24 h period rather than underutilized either during the day or night. The C available for growth is the amount of fixed C minus the C used in maintenance respiration, which is different between day (D) and night (N). The optimum starch sucrose partitioning (σ) will be when the growth rate during the day is the same as the growth rate during the night:

(10) $$A-A\;\cdot \;\sigma -R_{D}^{m}=\frac{A\;\cdot \;\sigma \;\cdot \;P}{\left(24-P\right)}-R_{N}^{m}$$

Solving for σ:

(11) $$\sigma =\frac{1-\left(R_{D}^{m}-R_{N}^{m}\right)/A}{\left(1+P/24-P\right)}$$

R*^m^_D_* and R*^m^_N_* denote maintenance respiration during the day and night, respectively. *P* denotes the photoperiod. Using data from day 44 of the growth model in Equation (10) indicates that 80% of C fixed during the day should be stored as starch (or other storage forms used at night). On the other hand, Sharkey et al. (1985), Gibon et al. (2009) and unpublished data from J. T. Yang, S. E. Weise, and T. D. Sharkey indicate that 60% is common and so this was used for σ in the model.

### State variables, parameters, inputs, and outputs

State variables and parameters of the *Arabidopsis* Leaf Area Growth Model are described in Table 1. Inputs of the model included seed or maternal characteristics (weight of storage reserves, ratio of mobilized storage reserves to stored reserves), leaf characteristics (initial leaf area, initial leaf mass, and the projected to total leaf area ratio), photosynthetic characteristics (net photosynthesis rate per unit leaf area), respiratory characteristics (leaf maintenance respiration, maintenance respiratory coefficients, growth respiratory coefficients), and partitioning coefficients (Table 1).

Initial “leaf” mass was set at 90% of the measured seed weight in Col-0 (Table 1). The ratio of mobilized storage reserves to stored reserves was set to 0.25 so that storage reserves would deplete by 4 days after seed sowing. The net photosynthesis rate was based on measurements obtained from Col-0 illuminated under a light intensity of 120 μmol m^−2^ s^−1^.

Initial leaf area was set at 5 × 10^−6^ m^2^. Leaf maintenance respiration per unit leaf area was 0.35 μmol CO_2_ m^−2^ s^−1^ (Givnish, 1988). Based on data from *Helianthus annuus*, root and inflorescence/stem maintenance respiratory coefficients were set to 0.016 and 0.007 μmol C g^−1^ s^−1^, respectively (Amthor, 1984) and leaf, inflorescence/stem, and root growth respiratory coefficients were set to 0.104, 0.17, and 0.13 g C g^−1^, respectively (Mariko, 1988). The rate of C exudation from roots was set to 0.001 μmol C m^−2^ h^−1^ based on unpublished data from S. E. Weise, and T. D. Sharkey. Root exudation was small enough to have no effect on the model but is included in the model as a mechanism for future exploration of hypotheses concerning partitioning to factors that include interactions with the rhizosphere and partitioning to defense compounds, for example glucosinolates.

In order to consider the effect of leaf area overlap on C assimilation and usage, the ratio of projected to total leaf area over time was entered to the model based on measured data from Col-0. Carbon concentration per gram (*C*) was taken to be 37,500 μmol g^−1^ dry mass of plant tissue based on reports that C content per unit of dry mass is around 45% (Schlesinger, 1991).

### Sensitivity analysis

Sensitivity of the model to variations in the inputs and assumptions was tested in Col-0 by simulating a 1% increase or decrease in NAR partitioned to growth processes, and other model inputs, and noting the resulting response in output. The model was deemed sensitive, if a change of more than 1% occurred in model outputs as a consequence of altering a specific input.

### Growth analysis

The model was tested by fitting it to measurements made with a standard laboratory strain of *Arabidopsis* (Col-0) and a strain known for large leaf growth (*gigantea-2, gi-2*). An extensive growth analysis was carried out to collect data needed to parameterize the model. Plants were grown in GC-20, Bigfoot series growth chambers (BioChambers Inc., Winnipeg, MB, Canada) in a hydroponics system using standing aerated nutrient solution technique. The hydroponics medium was ½ strength Hoagland's solution and plants were subjected to a light intensity of 120 μmol m^−2^ s^−1^ and an 8 h photoperiod, daytime and night time temperature of 22°C and 20°C respectively, and 60% relative humidity.

Growth measurements were taken throughout the life cycle of the plants at 3.5 week intervals and were collected just after gas exchange measurements. Before measuring gas exchange, photographs of intact rosettes were taken and later analyzed using ImageJ (http://imagej.nih.gov/ij/) software to determine projected leaf area. After measuring respiration at night, leaves were carefully separated from the rosette with the aid of a scalpel and fine tipped pair of forceps and the collections of separated leaves from each rosette were photographed and analyzed using ImageJ to determine the total leaf area. Data were used to obtain the ratio of projected to total leaf area.

At night, after photographs of total leaf area were taken, leaves, roots and inflorescences were harvested separately and freeze-dried for 48 h before measuring dry weights over time. Prior to germination, 100-seed weight was measured to obtain weight of a single seed. SLA, area-based relative growth rate (RGR_s_), RGR_M_, the leaf, stem and root mass ratios were determined.

### Measurement of leaf thickness

Leaves of similar age from 38-day old rosettes were harvested in the morning within 1 h of exposure to light. Avoiding the leaf midrib, ≈1 mm × 4 mm leaf sections were cut with the aid of a sharp scalpel blade in a watch glass containing fixative solution (2.5% glutaraldehyde, 2% paraformaldehyde, 0.1 M phosphate buffer—pH 7.4). Leaf sections were transferred to microfuge tubes containing fixative solution and placed at 4°C until further processing. Post-fixation processing, dehydration and embedding in epoxy resin followed by further excision into 500 nm thin sections with the aid of a PTXL ultramicrotome (RMC, Boeckeler Instruments, Tucson, AZ) was carried out at the Center for Advanced Microscopy, Michigan State University. Leaf cross sections were photographed and the distance between the adaxial and abaxial surfaces of the leaf (thickness) was measured by observing the leaf cross sections under an Olympus FluoView FV1000, Confocal Laser Scanning Microscope (Olympus, NJ, USA) located at the same institution. Leaf sections from leaves of three biological replicates from Col-0 and *gi-2* were used to obtain an average measurement of leaf thickness.

### Gas exchange measurements

Using a custom-built *Arabidopsis* rosette gas exchange cuvette connected to a LI-COR 6400 portable gas exchange system (LI-COR Environmental, Lincoln, NB), whole rosette photosynthesis and nighttime dark respiration was measured throughout the life cycle of the plants at 3.5 week intervals. The conditions in the *Arabidopsis* rosette gas exchange cuvette during photosynthesis measurements were: leaf temperature of 22°C, [CO_2_] of 400 ppm, and 120 μmol m^−2^ s^−1^ light intensity using a LED light source. During nighttime respiration measurements leaf temperature was maintained at 20°C.

### Parameterization of the model

Photosynthesis measurements obtained during four time points during the life cycle of Col-0 and *gi-2* were fitted with a polynomial 3rd order regression to extrapolate photosynthesis measurements through all time points of the life cycle. This equation was used in the model as input photosynthesis values. The measured ratio of projected to total leaf area obtained over time was fitted with a power equation to extrapolate the measurements through all time points of the life cycle and fitted to the model as an input.

The duration of each growth phase was entered for Col-0 and *gi-2* plants, based on the number of days required for flower initiation in each line along with other input parameters described above in Table 1.

The model was fitted to measured data from Col-0 and *gi-2* by adjusting all 16 partition coefficient parameters (partitioning to the inflorescence-ι, roots-ρ, leaf area growth-^s^λ, and leaf thickening-^t^λ, for each of the four growth phases), so that the modeled and measured leaf area, mass of stem, root and leaf matched (Figure 1). This means that variables that can be derived from those four, including whole plant mass, specific leaf area, leaf mass ratio, stem mass ratio, root mass ratio, RGR_S_, and RGR_M_, would also match. Modeled and measured data was considered as matched when the weighted absolute difference between the modeled data and the measured data were less than or equal to 1. The weighted absolute difference is a sigmoidal curve (Gibbs, 2000) defined as:

(12) $$Weighted\_diff=\sum \;\frac{1}{1+exp\left(\alpha \frac{dif}{SD}+\beta \right)}$$

In Equation (12), *dif* is the absolute difference between a modeled value and its corresponding measured value, *SD* is the standard deviation of the measured value, ∑ is the sum of all the weighted differences in one of the four measurement categories (leaf area, leaf mass, root mass and stem mass), and *exp* is the natural exponential function. In our experiment, we set α and β to be −10 and 5 respectively, so that the weighted absolute difference is equal to or greater than 1, if at least one absolute difference between a modeled value and its corresponding measured value falls outside the standard deviation of measured data.

Since there may exist multiple solutions satisfying Equation (12), a computer simulation is required. Specifically, considering leaf area, leaf mass, root mass and inflorescence/stem mass to be four independent objectives, we adopted a multi-objective optimization (MOO) solution for parameter tuning (Hwang and Masud, 1979; Miettinen, 1999). Mathematically, our multi-objective optimization problem can be formulated as:

(13) $$\begin{array}{l} \;min(weighted\_dif_{leaf\_area}(x),\;weighted\_dif_{leaf\_mass}(x),\\ weighted\_dif_{root\_mass}(x),\;weighted\_dif_{stem\_mass}(x))\;s.t.\;x\in X \end{array}$$

In Equation (13), the set *X* is the set of all feasible parameters. In multi-objective optimization, there does not typically exist a parameter setting that minimizes all the four objective functions simultaneously. Therefore, attention is paid to Pareto optimal solutions (Deb et al., 2003), which by definition are solutions that cannot be improved for any of the objectives without degrading at least one of the other objectives.

We implemented the *Arabidopsis* Leaf Area Growth Model in the R programming language (source code in Supplementary Data Sheet 1) and tuned all 16 parameters using a multi-objective optimization algorithm called non-dominated sorting genetic algorithm II (NSGA-II) (Deb et al., 2002) implemented in the R mco package (Mersmann, 2014). In each iteration of the optimization process, the NSGA-II algorithm approximates the Pareto front by adopting a fast non-dominated sorting approach, and then creates a mating pool by combining the parent and offspring populations and selecting the best individuals. The main advantage of using NSGA-II is that it can generate sets of parameter values, allowing exploration of the entire Pareto front.

Partitioning to leaves, roots and inflorescence/stem were constrained (Supplementary Table 1) based on prior measurements and physiological functions. For example, allocation to inflorescence was not allowed in the early vegetative period nor was zero allocation to roots. The model could give unrealistic results and take many generations to converge without these modest constraints.

**Figure 2:**
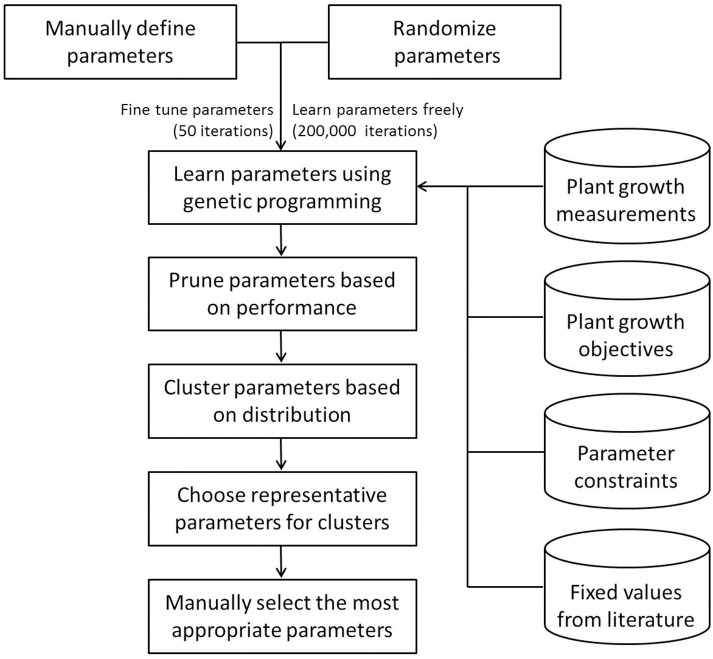
**Workflow of the parameterization process of the *Arabidopsis* Leaf Area Growth Model**. This schematic diagram illustrates key steps followed during parameterization of the *Arabidopsis* Leaf Area Growth Model. First, plant growth measurements, a literature survey, and expert opinion were used to generate parameters manually which allowed a reasonable match of modeled data to measured data and generation of parameter constraints (Supplementary Table 1) for the multi-objective optimization. Then the manual parameters were fine-tuned with genetic programming in R. Starting with random values, a number of qualified parameter settings were identified after 200,000 computer iterations of optimization steps based on four sets of objectives, such that the differences between modeled and measurements for each of leaf area and masses of leaf, inflorescence and root were less than or equal to 1. The large number of qualified parameter settings were subjected to a hierarchical clustering algorithm to categorize Col-0 and *gi-2* parameters into groups (Supplementary Table 2). From each cluster, the most representative and biologically feasible parameter settings were selected manually followed by further selection based on their best fit to leaf area and leaf mass.

The workflow of the parameterization process of the *Arabidopsis* Leaf Area Growth Model is shown in Figure 2. Without losing generality, we adopted two parallel approaches: (1) to estimate interactively the values of the parameters using expert knowledge, and (2) to determine the Pareto optimal values of the parameters using the multi-objective genetic algorithm with a randomized initial population of feasible parameters. While the former only needs a few iterations, the latter requires thorough simulation. To this end, we parallelized the original NSGA-II function in the mco package, enabling the use of a high performance computing cluster to reduce the total computational time required (source code in Supplementary Data Sheet 1). The combined results of the two approaches consisted of a set of parameter values, which were clustered and ranked. The top two parameter settings for each genotype were selected with an emphasis on the fit to leaf area and leaf mass for further analysis.

### Statistical analyses of experimental data

Experimental data for growth and gas exchange was collected from 10 plants per line at a given time point. Leaves from 3 biological replicates were used to obtain measurements of leaf thickness. Statistical analyses were carried out using SPSS 18 (IBM Corporation, NY, NY). The effect of the genotype was tested with a univariate general linear model and data were subjected to One-Way ANOVA at α = 0.05. Measured data presented in figures represent the mean ± standard error (SE) or, where appropriate, mean ± standard deviation (SD) and is specified in the figure legends.

## Results

### Mutant line *gi-2* exhibits greater leaf area, leaf, and plant mass, specific leaf area, and relative growth rate than Col-0

**Figure 3:**
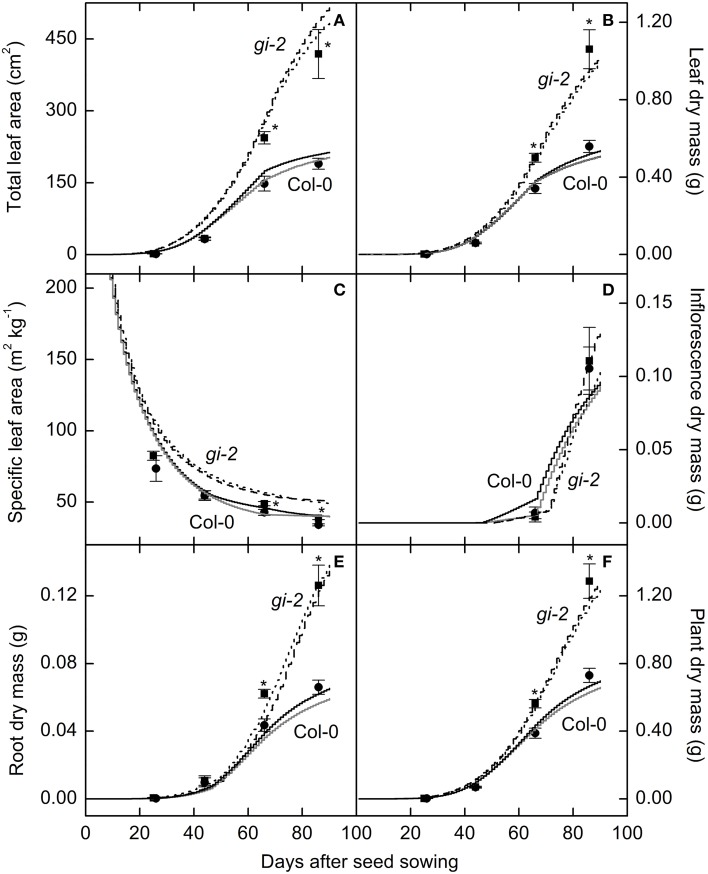
**Comparison of modeled and measured leaf and plant growth over time in Col-0 and *gi-2***. Modeled data generated using the selected parameter settings (**Table 2**) is compared to measured data for total leaf area **(A)**, leaf dry mass **(B)**, specific leaf area **(C)**, and dry masses of the inflorescence **(D)**, root **(E)**, and the entire plant **(F)**. Modeled data for Col-0 from simulation 1 (solid black lines) and simulation 2 (solid gray lines) and for *gi-2* from simulation 1 (dotted lines) and 2 (dashed lines) is given. Simulated data is from 1 to 90 DAS. Measured data for Col-0 (filled circles) and *gi-2* (filled squares) was initially taken at 26 DAS for Col-0 and 25 DAS for *gi-2* and at 44, 66, 86 DAS for both lines. Measured values represent the mean ± SE and *n* = 10 plants per line. Measurements of *gi-2* which showed a statistically significant difference from Col-0 at α = 0.05 are marked with an asterisk (^*^).

**Figure 4:**
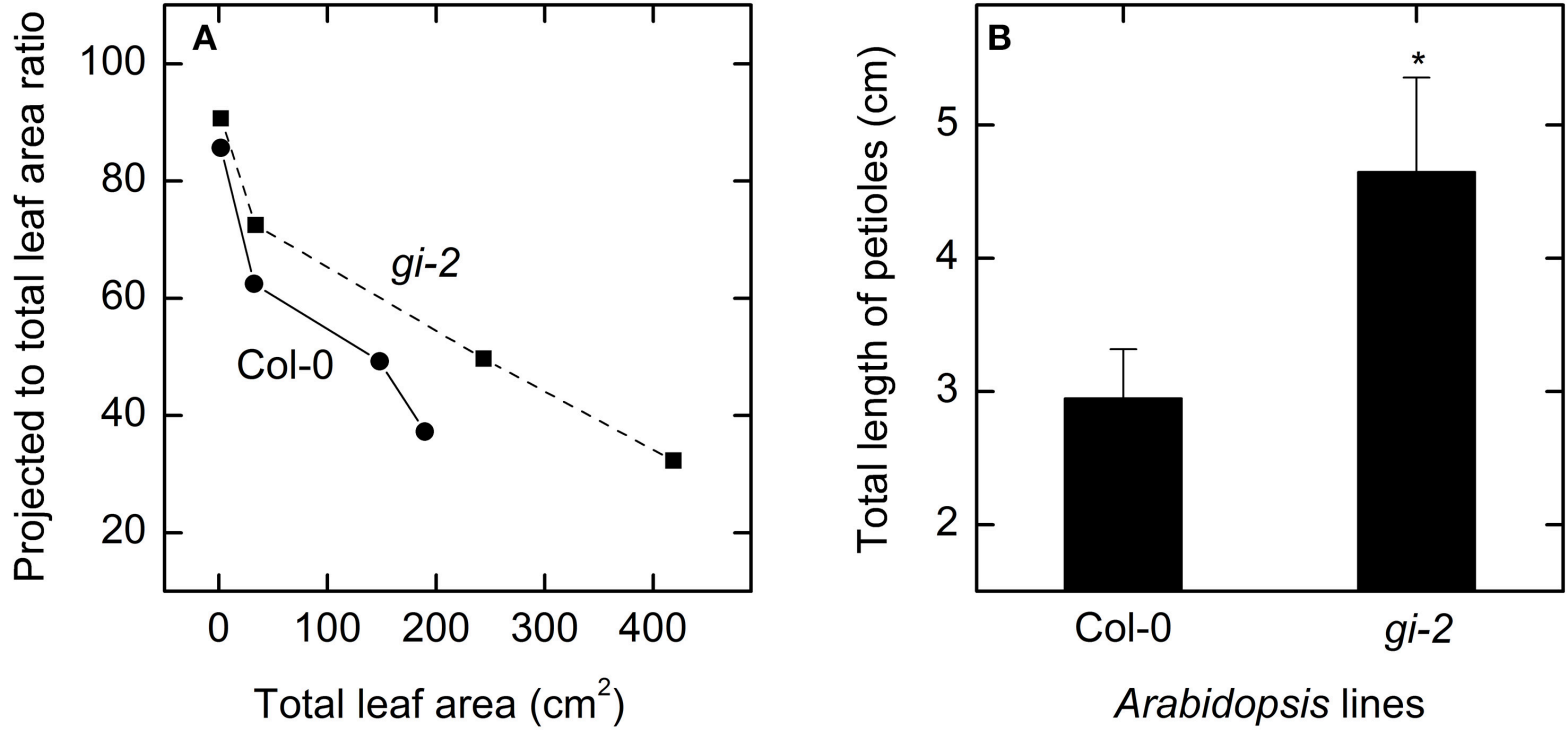
**Comparison of leaf overlap and petiolar length overtime in Col-0 and *gi-2***. A comparison between projected to total leaf area ratio and total leaf area for Col-0 (solid line and filled circles) and *gi-2* (dashed line and filled squares) **(A)** and the total length of petioles **(B)** is shown. In **(A)**, the 1st measurement (lowest leaf area and highest projected to total leaf area ratio) was taken at 26 DAS for Col-0 and 25 DAS for *gi-2* and the remaining data at 44, 66, 86 DAS for both lines, and values represent the average of 10 measurements from 10 plants per line. In **(B)**, data was taken at 26 DAS for Col-0 and 25 DAS for *gi-2* and values represent the mean ± SE and *n* = 10 plants per line. Measurements of *gi-2* which showed a statistically significant difference from Col-0 at α = 0.05 are marked with an asterisk (^*^).

Measured data for leaf area and leaf, root, and plant masses followed a logistic growth pattern (Figure 3). Total leaf area of *gi-2* was more than 2 times larger at 86 DAS than that in Col-0 (Figure 3A, Supplementary Figure 1 in Presentation 1). Leaves of *gi-2* showed 5–10% less leaf overlap than that in Col-0 (Figure 4A) in large measure because petioles were longer (Figure 4B).

**Figure 5:**
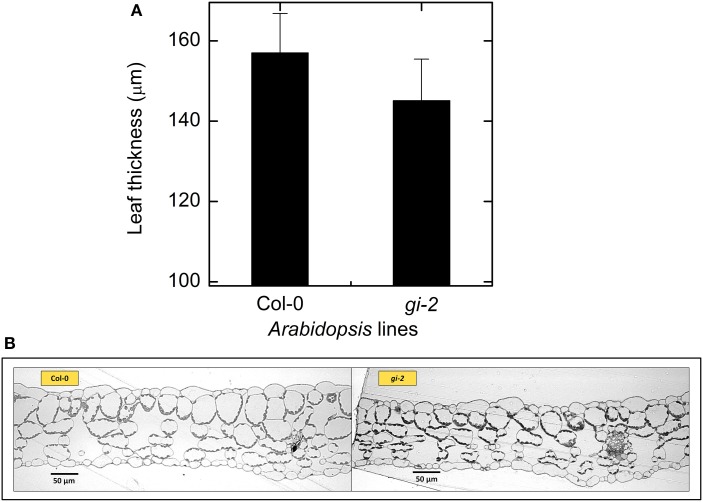
**Comparison of leaf thickness in Col-0 and *gi-2***. A comparison between leaf thickness measured from leaf sections of rosette leaves harvested from 38-day old plants **(A)** and representative photographs of leaf cross sections **(B)** for Col-0 and *gi-2* is given. In **(A)** values represent the mean ± SE and *n* = 3 plants per line.

Leaf, root, and whole plant dry weight of *gi-2* was about 1.5 times larger than Col-0 at 66 DAS (Figures 3B–F). At 86 DAS, leaf, root, and plant mass of *gi-2* was 2 times greater than that in Col-0. A decrease in SLA over time in both Col-0 and *gi-2*, indicated that leaf thickness may increase with plant age (Figure 3C). Throughout its life cycle, specific leaf area of *gi-2* was 12–15% greater than Col-0. Correspondingly, leaf thickness measured at 38 DAS showed that leaves of Col-0 were 8% thicker than *gi-2* leaves, even though this difference was not statistically significant (Figure 5A). Shorter palisade cells in *gi-2* may have contributed to reduced leaf thickness than that in Col-0 (Figure 5B).

Leaf area ratio or leaf area per unit plant mass was 13 and 23% greater in *gi-2* during the late vegetative and reproductive phase, respectively, than in Col-0 (Supplementary Figure 2A in Presentation 1). Leaf mass constituted close to 80% of plant biomass (Supplementary Figure 2B in Presentation 1). Comparatively, leaf mass ratio in *gi-2* was less than that in Col-0 at 26 DAS, but, was significantly greater at the final harvest. Mutant line *gi-2* also exhibited a greater root mass ratio and a smaller stem mass ratio than Col-0 at 86 DAS (Supplementary Figures 2C,D in Presentation 1).

**Figure 6:**
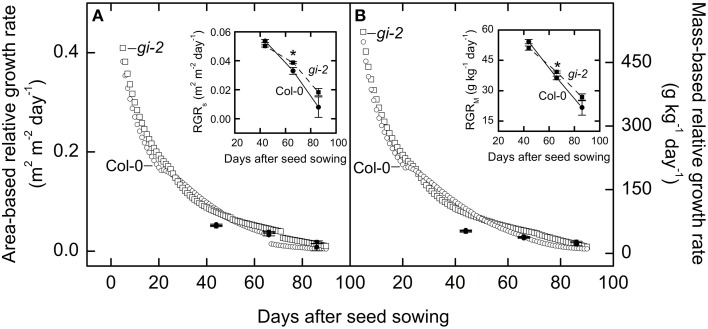
**Comparison of area-based and mass-based relative leaf growth rate over time in Col-0 and *gi-2***. Modeled data generated using partitioning coefficients in simulation 1 (**Table 2**) and measured data (also shown expanded in smaller panels) for area-based relative growth rate (RGR_S_) or relative increase in leaf area **(A)** and mass-based relative growth rate (RGR_M_) or relative increase in plant mass **(B)** for Col-0 and *gi-2* is provided. Modeled data for Col-0 (open circles) and *gi-2* (open squares) is simulated from 5 to 90 DAS. Relative growth rates measured for Col-0 (solid lines and filled circles) were calculated from 26 to 44 DAS, 44 to 66 DAS, and 66 to 86 DAS. Relative growth rates measured for *gi-2* (dashed lines and filled squares) was calculated from 25 to 44 DAS, 44 to 66 DAS, and 66 to 86 DAS. For measured data, values represent the mean ± SE and *n* = 10 plants per line. Measurements of *gi-2* which showed a statistically significant difference from Col-0 at α = 0.05 are marked with an asterisk (^*^).

Measured data indicated that mutant line *gi-2* exhibited slightly greater area-based and mass-based relative growth rates than Col-0 at 66 DAS and after (Figure 6). Modeled data revealed that *gi-2* maintained greater area-based and mass-based relative growth rates even earlier during the life cycle (Figure 6).

Flower initiation occurred on the 67th and 72nd day after seed sowing in Col-0 and *gi-2*, respectively, and this delay in transition from vegetative to the reproductive phase in *gi-2* was statistically significant (data not shown). During previous studies (Fowler et al., 1999), a more significant delay in flower initiation has been observed in *gi-2*. Use of short-day conditions during the present study compared to long-day conditions used by Fowler et al. (1999) may have reduced the delayed flowering phenotype in *gi-2*.

### Mutant line *gi-2* exhibits lower area-based photosynthesis

**Figure 7:**
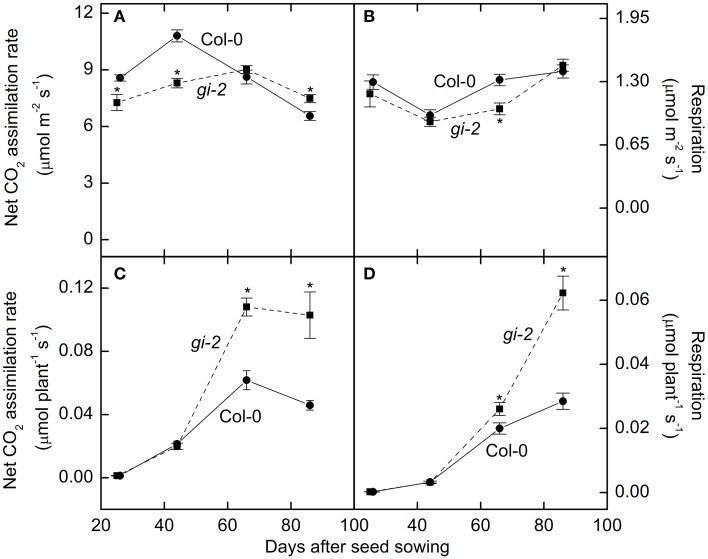
**Comparison of photosynthesis and respiration overtime in Col-0 and *gi-2***. Area-based photosynthesis **(A)**, area-based nighttime respiration **(B)**, photosynthesis on a whole plant basis **(C)**, and nighttime respiration on a whole plant basis **(D)** is shown for Col-0 (solid lines and filled circles) and *gi-2* (dashed lines and filled squares). The 1st measurement was taken at 26 DAS for Col-0 and 25 DAS for *gi-2* followed by 44, 66, 86 DAS for both lines. Values represent the mean ± SE and *n* = 10 plants per line. Measurements of *gi-2* which showed a statistically significant difference from Col-0 at α = 0.05 are marked with an asterisk (^*^).

Photosynthesis per unit leaf area was 15–23% lower in *gi-2* compared to that of Col-0 throughout the vegetative phase (Figure 7A). Area-based nighttime respiration rates remained similar between the 2 lines except at 66 DAS when *gi-2* exhibited lower area-based respiration than that in Col-0 (Figure 7B). Whole plant photosynthesis measured at 26 DAS and 44 DAS did not differ between the two lines (Figure 7C). However, by 66 DAS and 86 DAS, photosynthesis on a whole plant basis was 75 and 124% higher, respectively, in *gi-2* compared to that in Col-0 (Figure 7C). As a result of its larger rosette mass, respiration on a whole rosette basis was greater in *gi-2* than in Col-0 during the later stages of growth (Figure 7D).

### Computational parameterization of energy partitioning parameters for Col-0 and *gi-2*

We ran the parameterization process (i.e., optimization and clustering) including an optimization of 200,000 iterations on the Intel Xeon 1024-core distributed memory computer cluster at Michigan State University. The process identified 84 and 95 qualified parameter settings for Col-0 and *gi-2*, respectively (Supplementary Table 2). The density distributions of the four objectives (leaf area, leaf mass, root mass and stem mass) indicated that all of the parameters matched with measured data (Supplementary Figure 3 in Presentation 1).

The objective correlation figure revealed that there was a trade-off between the goodness of fit with respect to measurements of leaf area and biomass, resulting in a very distinctive Pareto front between these two objectives (Supplementary Figure 4 in Presentation 1). This Pareto front was clearest for the fit to the wild-type Col-0 and less so for the *gi-2* mutant. The existence of this trade-off justifies the need to use multi-objective optimization.

For the rest of the objectives (i.e., leaf, inflorescence and root biomass), no clear Pareto front can be discerned by means of visual analysis of the plots generated for each of the pairwise combinations. Rather, one can identify the existence of a dominant peak toward the origin of each plot, indicating that parameters that produce a good fit for one of those objectives tend to produce a good fit for the rest of the objectives.

The objective correlation figure also showed the marginal empirical distribution of the residuals of each individual objective (Supplementary Figure 4 in Presentation 1). They were all skewed toward the lower values, an indication that, for each objective, the majority of solutions minimize the differences between model and data, which is what one would expect if the algorithm was converging adequately. The presence of a smaller local maximum at higher values of the residuals in these distributions is the result of the existence of a trade-off between fitting leaf area and biomass.

**Figure 8:**
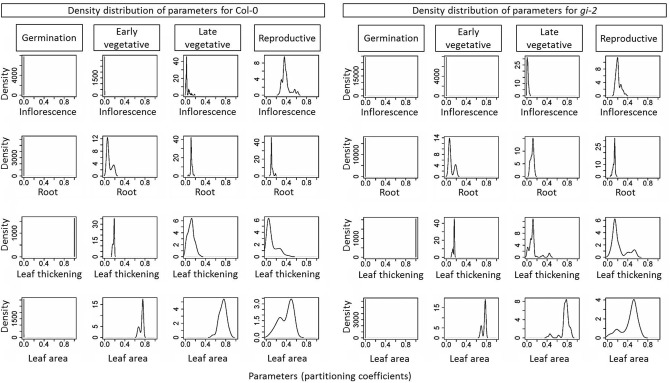
**The density distribution of learned parameters from the *Arabidopsis* Leaf Area Growth Model**. Density distribution of all 16 parameters namely, partitioning coefficients of C partitioning to inflorescence, root, leaf thickening and leaf area growth for four growth phases: germination phase, early and late vegetative phases, and reproductive phase as learned by the *Arabidopsis* Leaf Area Growth Model is given. The 84 identified qualified parameter settings for Col-0 and the 95 identified parameter settings for *gi-2* used to determine the densities are given in Supplementary Table 2.

Note that the residuals presented in the objective correlation figure (Supplementary Figure 4 in Presentation 1) are aggregated for the entire simulation. Thus, if there are trade-offs between objectives in specific, short growth stages, this may not become apparent in these figures. However, analysis of the distribution of parameters (Figure 8) allows us to infer the existence of trade-offs if such distributions were to present bimodality (i.e., two local maxima), which generate the so-called Pareto sets (linked to the corresponding Pareto fronts in objective space). Indeed, the distribution of values for each parameter showed two distinct patterns: (1) most parameters in both the seed and the late vegetative stages were unimodal with low variance, resulting in single, narrow peaks; and (2) most parameters in both the early vegetative and the reproductive stages were bimodal. For example, the partitioning coefficient to roots and leaf area were strongly bimodal in the early vegetative stage (Figure 8). This meant that there were two clusters of values for these two parameters that resulted in a Pareto front for that particular growth stage (i.e., one cluster fitted better leaf area and the other fitted better root mass). A similar pattern could be detected for the partitioning to leaf biomass and leaf area in the reproductive stage.

Overall, the existence of Pareto fronts of objectives and Pareto sets of parameters suggested that there were more than one optimal parameter setting for each genotype. Indeed, the parameter clustering analysis revealed four distinct parameter settings for Col-0 and five distinct parameter settings for *gi-2* (Supplementary Figure 5 in Presentation 1).

Following the workflow in Figure 2, we chose two parameter settings from simulation results for further analysis (Table 2). The parameter setting with the lowest score out of the two selected sets for Col-0 and *gi-2* are designated as simulation 1 and the other two as simulation 2. Any of the two parameter settings for Col-0 can be compared to any of the two parameter settings for *gi-2*. For user convenience and visualization purposes, the *Arabidopsis* Leaf Area Growth Model is provided as an excel file (Model 1 - Supplementary Data Sheet 2, Model 2 - Supplementary Data Sheet 3). Model 1 (Supplementary Data Sheet 2) provides modeled plant growth for Col-0 and *gi-2* using partitioning coefficients in simulation 1 (Table 2). Model 2 (Supplementary Data Sheet 3) provides modeled plant growth for Col-0 and *gi-2* using partitioning coefficients in simulation 2 (Table 2). The R-code for the model is provided in Supplementary Data Sheet 1.

**Table 2:** **Comparison of modeled partition coefficients for Col-0 and *gi-2* during different growth phases**.

**Partition coefficients**	**Growth phase**					
	**Early vegetative**	**Late vegetative**	**Reproductive**	**Early vegetative**	**Late vegetative**	**Reproductive**
	**Simulation 1 for Col-0**			**Simulation 1 for *gi-2***		
ι	0.01	0.06	0.34	0.00	0.02	0.20
ρ	0.06	0.11	0.10	0.08	0.12	0.14
^t^λ	0.18	0.13	0.23	0.15	0.12	0.15
^s^λ	0.75	0.70	0.32	0.77	0.74	0.51
	**Simulation 2 for Col-0**			**Simulation 2 for *gi-2***		
ι	0.00	0.02	0.40	0.00	0.02	0.25
ρ	0.05	0.11	0.10	0.06	0.11	0.14
^t^λ	0.19	0.20	0.05	0.16	0.12	0.07
^s^λ	0.76	0.67	0.45	0.78	0.76	0.54

*Learned partitioning coefficients from the Arabidopsis Leaf Area Growth Model for partitioning of NAR or net assimilated C available for growth to inflorescence (ι) and root growth (ρ), leaf thickening (^t^λ), and leaf area growth (^s^λ) during the early and late vegetative phases and the reproductive phase for Col-0 and gi-2 are given. The durations of the early and late vegetative phases and the reproductive phase is 5–45 DAS, 46–66 DAS, and 67–90 DAS, respectively, for Col-0 and 5–49 DAS, 50–71 DAS, and 72–90 DAS, respectively for gi-2*.

Modeled *Arabidopsis* plant growth in terms of leaf area and leaf, inflorescence, root, and plant mass increase followed a logistic growth pattern (Tessmer et al., 2013) and the two sets of parameters or partitioning coefficients selected for each line (Table 2) produced realistic growth trajectories for Col-0 and *gi-2* (Figure 3, Models 1, 2 in Supplementary Data Sheets 2, 3). For example, based on the initial inputs of values typical for *Arabidopsis* Col-0, and learned partitioning coefficients given in simulation 1 (Table 2), the model produced a plant with a leaf area of 209 cm^2^ comparative to measured total leaf area of 190 cm^2^ at 86 DAS (Figure 3A, Model 1-Supplementary Data Sheet 2); using learned partition coefficients given in simulation 2 (Table 2), the model produced a plant with a final leaf area of 197 cm^2^ at 86 DAS (Figure 3A, Model 2-Supplementary Data Sheet 3). Modeled leaf, inflorescence, root and plant mass of Col-0 was also comparable with measured data (Figures 3B–F, Models 1, 2 in Supplementary Data Sheets 2, 3). Similarly, the model produced a *gi-2* plant with a leaf area of 463 and 489 cm^2^ using partition coefficients in simulation 1 and simulation 2, respectively, comparative to measured total leaf area of 418 cm^2^ at 86 DAS (Table 2, Figure 3A, Models 1, 2 in Supplementary Data Sheets 2, 3). The modeled leaf, inflorescence, root, and plant mass of the modeled plant for *gi-2* was also comparable with measured data (Figures 3B–F, Models 1, 2 in Supplementary Data Sheets 2, 3).

The model also implemented the partitioning of photosynthetic C to starch and sucrose successfully. The available C for respiratory processes, exudation and growth during daytime was greater than that available at night (Supplementary Figure 6A in Presentation 1, Models 1, 2 in Supplementary Data Sheets 2, 3), following a realistic diel pattern (Pokhilko et al., 2014). NAR showed a similar pattern, but, NAR available at night was lower than during the day consistent with using a partitioning value of 0.6 rather than the theoretically optimum for an 8 h day of 0.8 (Supplementary Figure 6B in Presentation 1, Models 1, 2 in Supplementary Data Sheets 2, 3). The main cause of reduced NAR at night was the large cost associated with leaf respiration. The modeled *Arabidopsis* plant grew both during the daytime and nighttime (Supplementary Figure 6C in Presentation 1). However, corresponding with limited C availability at night, both area-based growth rate and mass-based growth rates were faster during the day than night (Models 1, 2 in Supplementary Data Sheets 2, 3). Based on model outputs, maximum relative growth rates could be seen during early morning hours, which is in accordance with diel leaf growth patterns known for *Arabidopsis* and other dicot species (Walter et al., 2009; Friedli and Walter, 2015).

**Figure 9:**
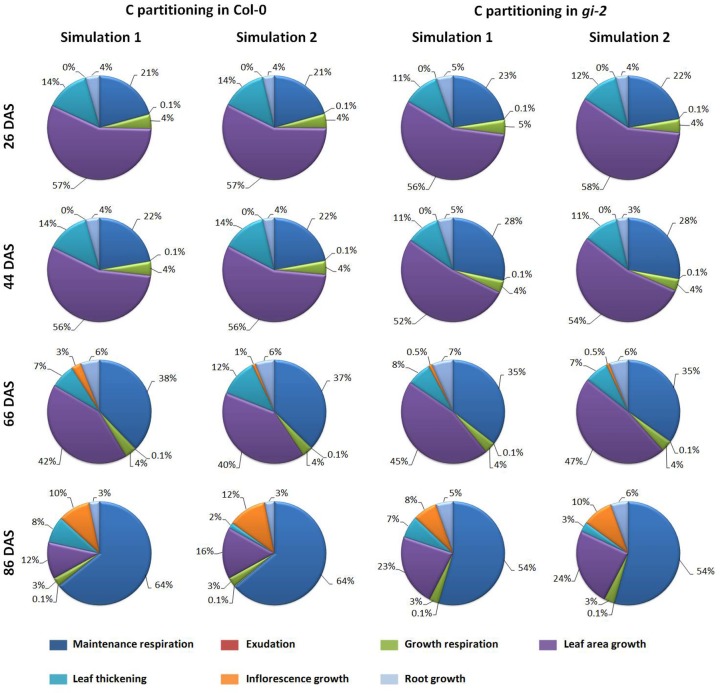
**Modeled changes in C partitioning to respiration and growth processes overtime in *Arabidopsis***. The amounts of C partitioned to drive maintenance and growth respiration, exudation, inflorescence, root and leaf area growth and leaf thickening is presented as percentages of the daily available C at 26, 44, 66, and 86 DAS, based on the outputs of the *Arabidopsis* Leaf Area Growth Model using learned parameters from simulation 1 and 2 for Col-0 and *gi-2* given in Table 2.

Learned partitioning coefficients provided in Table 2 were used to determine how much C as a proportion of total available C is being allocated to maintenance and growth respiration, exudation and to growth of different plant organs (Figure 9). The modeled data showed that during early stages of development, close to 3/4 of available C is reserved for growth excluding growth respiration. Most of this C was used for leaf area growth and leaf thickening. As the plant matured, maintenance respiratory costs gradually increased and accounted for half or more of the available C in the mature plant (Figure 9).

According to learned parameter coefficients of the *Arabidopsis* Leaf Area Growth Model, a major portion of NAR or net assimilated C available for growth was allocated to leaf growth throughout the plant's life cycle (Table 2, Models 1, 2 in Supplementary Data Sheets 2, 3). Both lines partitioned more than 90, 80, and 50% of NAR to leaf growth during the early and late vegetative, and reproductive phases, respectively. In addition, a major portion of C allocated for leaf growth (close to 80% or more in most cases) was partitioned to drive leaf area growth in both *Arabidopsis* lines (Table 2). The model also indicated that the amount of C partitioned to leaf growth was reduced by 40–50% upon transition to the reproductive phase as a result of diversion of C to support the growth of the inflorescence. In general, a smaller proportion, which was around 5–14% of NAR, was partitioned to roots throughout the life span in comparison to leaves and inflorescence (Table 2).

One important factor revealed by the *Arabidopsis* Leaf Area Growth Model with fitted data from Col-0 and *gi-2* was the significant amount of C being partitioned to leaf thickening throughout the life cycle (Table 2, Models 1, 2 in Supplementary Data Sheets 2, 3). During the early vegetative phase, up to 19 and 16% of NAR was partitioned to leaf thickening in Col-0 and *gi-2*, respectively. Comparatively, Col-0 and *gi-2*, partitioned only up to 6 and 8% of NAR, respectively, to roots and up to 1% to inflorescence growth during the same growth phase. During the late vegetative phase, simulations indicated a decrease in C partitioning to leaf thickening as C was diverted to support the developing inflorescence and root growth. For example, during the late vegetative phase, up to 20% of NAR in Col-0 and 12% in *gi-2* was partitioned to leaf thickening. Correspondingly, NAR partitioned to root growth increased up to 11% in Col-0 and 12% in *gi-2* and NAR partitioned to inflorescence growth increased up to 6% in Col-0 and 2% in *gi-2* (Table 2, Models 1, 2 in Supplementary Data Sheets 2, 3). Simulations indicated that NAR partitioned to leaf thickening can be as high as 23% in Col-0 and 15% in *gi-2* depending on the demand for C by the inflorescence and roots during the reproductive phase.

Corresponding with the gradual decrease in NAR or net assimilated C partitioned to leaf area growth with plant age the modeled SLA decreased with time (Table 2, Figure 3C, Models 1, 2 in Supplementary Data Sheets 2, 3).

### Sensitivity analysis

Sensitivity of the *Arabidopsis* Leaf Area Growth Model to variations in input parameters was tested using parameterizations for Col-0 and the key findings are summarized in Table 3. Users can test sensitivity of the model to any parameter using the designated page for sensitivity analysis provided in Models 1, 2 (Supplementary Data Sheets 2, 3).

**Table 3:** **Sensitivity of the *Arabidopsis* Leaf Area Growth Model to key model inputs**.

**Model input and alteration**		**% change in leaf area**				**% change in plant mass**			
		**26 DAS**	**44 DAS**	**66 DAS**	**86 DAS**	**26 DAS**	**44 DAS**	**66 DAS**	**86 DAS**
Photosynthesis	(+1)	+4	+4	+5	+5	+4	+5	+6	+6
	(−1)	−4	−4	−4	−5	−4	−5	−5	−6
Partitioning to starch (σ)	(+1)	−0.5	−0.5	−0.5	−0.5	−0.6	−0.6	−0.6	−0.6
	(−1)	+0.5	+0.5	+0.5	+0.5	+0.6	+0.6	+0.6	+0.6
Initial leaf area	(+1)	+4	+4	+5	+5	+4	+4	+5	+5
	(−1)	−4	−4	−5	−5	−3	−4	−4	−4
^t^λ —Early vegetative phase^†^	(+1)	−15	−24	−28	−28	−12	−21	−25	−26
	(−1)	+19	+34	+42	+43	+14	+28	+36	+39
ρ —Early vegetative phase^††^	(+1)	+11	+21	+28	+29	+8	+17	+23	+25
	(−1)	−10	−17	−20	−21	−7	−14	−18	−19
ι —Late vegetative phase^*^	(+1)	0	0	+0.6	+0.8	0	0	0	+0.1
	(−1)	0	0	−0.6	−0.8	0	0	0	−0. 1
Length of vegetative phase	(+1)	0	0	0	0	0	0	0	0
	(−1)	0	0	0	0	0	0	0	0

*Significant results of the sensitivity analysis of the Arabidopsis Leaf Area Growth Model parameterized with measured data from Col-0 and learned parameters from simulation 1 for Col-0 (Table 2) is presented. Magnitude of inputs were either increased (+) or decreased (−) by 1% and resulting changes in modeled leaf area and plant mass is presented as a % of their initial value at 26, 44, 66, and 86 DAS*.

† *Increasing and decreasing ^t^λ, decreased and increased ^s^λ, respectively*.

†† *Increasing and decreasing ρ, decreased and increased ^t^λ, respectively, while ^s^λ was kept constant*.

* *Increasing and decreasing ι, decreased and increased ^t^λ, respectively, while ^s^λ was kept constant. The symbols ι, ρ, ^s^λ, and ^t^λ stand for partition coefficients of net assimilated C partitioning to the inflorescence, roots, leaf area growth and leaf thickening, respectively*.

Modeled leaf area growth and plant mass was most sensitive to C partitioned to leaf thickening and area growth during the early vegetative phase (Table 3). For example, a 1% decrease in partitioning to leaf thickening with a corresponding 1% increase in partitioning to leaf area growth lead to a 43 and 39% increase in modeled leaf area and plant mass, respectively, by 86 DAS. A 1% increase in partitioning to leaf thickening with a corresponding 1% decrease in partitioning to leaf area growth lead to a 28 and 26% decrease in modeled leaf area and plant mass, respectively, by 86 DAS (Table 3). Interestingly, modeled leaf, root, inflorescence masses were also highly sensitive to changes in C partitioning to leaf area growth/leaf thickening during the early vegetative phase (data not shown). For example, decreasing partitioning to leaf thickening by 1% with a corresponding 1% increase in partitioning to leaf area growth lead to a 36, 39, and 37% increase in modeled leaf, inflorescence and root mass, respectively, by 86 DAS.

Modeled leaf area growth and plant mass was also highly sensitive to C partitioned to root growth during the early vegetative phase (Table 3). However, sensitivity analysis revealed that type and magnitude of impact on leaf area growth and overall plant growth by changes made to the root partitioning coefficient depends on whether the C is extracted from or allocated toward leaf area growth and/or leaf thickening. For example, if C partitioning to root growth was increased by 1% with a corresponding 1% decrease in partitioning to leaf thickening, modeled leaf area and plant mass increased by 29 and 25% by 86 DAS (Table 3). Root mass increased by 28%. In contrast, if an increase in C partitioned to root growth is modeled with a corresponding decrease in C for leaf area growth, there was a negative effect on modeled leaf area growth and other plant growth characteristics. For example, if C partitioning to root growth was increased by 1% with a corresponding 1% decrease in partitioning to leaf area growth, modeled leaf area and plant mass decreased by 10% and root mass decreased by 8% by 86 DAS (data not shown).

Modeled leaf area growth and plant mass was also sensitive to photosynthesis and initial leaf area (Table 3). Interestingly, sensitivity analysis revealed that the sensitivity of growth parameters to C partitioning to leaf thickening and area growth is far greater than their sensitivity to changes in photosynthesis. The modeled leaf area growth or plant mass was not sensitive to changes in C partitioning to starch (Table 3). This reflects the input assumption that growth can occur day or night and no specific penalty was given for shifting growth between day and night.

Interestingly, sensitivity analysis revealed that neither the leaf area nor plant mass was sensitive to changes in partitioning of growth C to inflorescence growth during the late vegetative phase (Table 3). However, increasing the C partitioning to inflorescence growth by 1% during the late vegetative phase led to a 4% increase in inflorescence mass and vice versa. Sensitivity analysis also revealed a 1% increase or decrease in the number of days required for flower initiation has no effect on leaf area growth or plant mass (Table 3) nor on leaf, root or inflorescence mass (data not shown).

Small changes during the early vegetative phase, to C partitioning coefficients of partitioning of NAR to growth processes, translated to moderate changes in leaf area growth and overall plant growth toward the end of the early vegetative phase, but, led to significant changes in growth by later stages of the life cycle (Table 3). However, changes made to partitioning coefficients of leaf thickening, area growth or root growth during the late vegetative phase led to only minor changes in final leaf area, root, leaf or plant mass (data not shown). Leaf and plant growth was not sensitive to changes made to partitioning coefficients during the reproductive phase. Thus, the model realistically simulated plant growth in the sense that leaf and plant growth was most sensitive to changes in C partitioning during the early vegetative phase (Models 1, 2 in Supplementary Data Sheets 2, 3).

### Mutant line *gi-2* partitioned less NAR to leaf thickening and more to leaf area growth and maintained a greater specific leaf area

Interestingly, one of the main differences between the two lines as revealed by the model was the greater partitioning of NAR to leaf area growth and reduced partitioning to leaf thickening in *gi-2* and vice versa in Col-0 (Table 2, Models 1, 2 in Supplementary Data Sheets 2, 3). For example, according to model simulation 1, although Col-0 allocated 93, 83, and 55% of NAR to drive leaf growth during the early vegetative, late vegetative and reproductive phases, respectively, only 81, 84, and 58% of this C (or 75, 70, 32% of total NAR) was partitioned to support leaf area growth, respectively. In contrast, according to model simulation 1, *gi-2* partitioned 92, 86, and 66% of NAR to leaf growth during the early vegetative, late vegetative and reproductive phases, respectively, out of which 87, 86, and 77% (or 77, 74, 51% of total NAR) was partitioned to drive leaf area growth.

In other words, partition coefficients in simulation 1 for Col-0 and *gi-2*, indicated that the latter partitioned 3, 1, and 8% less NAR during the early vegetative, late vegetative and reproductive phases, respectively, to leaf thickening than Col-0 (Table 2). Measured data provided evidence for reduced leaf thickness in *gi-2* (Figure 5). The sensitivity analysis (Table 3) indicated that even small differences in partitioning to leaf thickening and leaf area growth, especially early in the life cycle, could have a large effect on the final leaf area and subsequently on plant growth. The *gi*-2 mutant accumulates starch in leaves, especially when they begin to flower (Eimert et al., 1995). This might be expected to reduce growth but instead growth was enhanced in *gi-2*. Starch accumulation was not required for the late flowering phenotype of *gi-2* (Eimert et al., 1995) and was not associated with reduced growth in this study. With additional data this model could be used to determine how starch accumulation could affect overall C balance and growth.

Similar to measured data, modeled data indicated that despite the ontogenic decrease, SLA, remained significantly higher in *gi-2* than that in Col-0 throughout the life cycle (Figure 3C, Models 1, 2 in Supplementary Data Sheets 2, 3). Based on modeled partitioning coefficients, this can be attributed to enhanced partitioning of NAR to leaf area growth in *gi-2* (Table 2, Models 1, 2 in Supplementary Data Sheets 2, 3).

Partitioning patterns similar to above could also be seen when C being partitioned to respiration and growth was expressed as proportions of the total available C (Figure 9, Models 1, 2 in Supplementary Data Sheets 2, 3). The model revealed that *gi-2* partitioned a lower proportion of total available C to leaf thickening throughout the life cycle (Figure 9). Although, the partitioning coefficients of partitioning of NAR to leaf area growth was greater in *gi-2*, the proportions of C partitioned to leaf area growth out of the total available C, seem to be lower in *gi-2* at 26 DAS and 44 DAS (days on which the 1st two measurements were taken). However, at 26 DAS and 44 DAS, modeled available C in *gi-2* was 56 and 11% greater, respectively, compared to that in Col-0 (Supplementary Figure 6A in Presentation 1, Models 1, 2 in Supplementary Data Sheets 2, 3). However, when both maintenance and growth respiratory costs are excluded, a greater proportion of remaining C was allocated to leaf area growth in *gi-2* than that in Col-0 (Figure 9). By 66 DAS, *gi-2* partitioned a greater proportion of total C to leaf area growth, root growth and lower proportions to inflorescence growth than that in Col-0 (Figure 9, Models 1, 2 in Supplementary Data Sheets 2, 3). By 66 DAS, *gi-2* also partitioned lower proportions of the total available C to maintenance respiration compared to that in Col-0.

### Mutant line *gi-2* partitioned less NAR to inflorescence growth

The *Arabidopsis* Leaf Area growth Model revealed reduced partitioning of NAR to inflorescence growth in *gi-2* which was more evident in the reproductive phase compared to that in Col-0 (Table 2, Models 1, 2 in Supplementary Data Sheets 2, 3). This was also evident by measured inflorescence mass ratios (Supplementary Figure 2C in Presentation 1). However, both measured and modeled data showed that inflorescences of *gi-2* was similar in growth in terms of mass, if not larger, despite reduced partitioning to inflorescence growth in *gi-2* (Figure 3D). As modeled inflorescence growth was highly sensitive to partitioning of NAR to leaf area growth during the early vegetative phase, and least sensitive to the length of the vegetative phase (data not shown), reduced partitioning of NAR to inflorescence growth and a shorter reproductive phase in *gi-2*, may have been compensated by an increase in C partitioning to leaf area growth with subsequent increases in assimilated C.

### Mutant line *gi-2* partitioned more NAR to root growth

Modeled data supported findings from measured data that *gi-2* partitioned more C to root growth than Col-0 (Table 2, Supplementary Figure 2D in Presentation 1, Models 1, 2 in Supplementary Data Sheets 2, 3). Although this trend could be seen throughout the life cycle, it was most apparent during the reproductive phase. For example, both model simulations for Col-0 indicated that 10% of NAR was been partitioned to root growth during the reproductive phase compared to 14% in *gi-2*. Sensitivity analysis revealed that an enhancement in C partitioned to root growth during the early vegetative phase without compromising C partitioned to leaf area growth can have a positive effect on leaf area, root and plant growth (Table 3).

### Relationship between leaf area growth and plant growth

**Figure 10:**
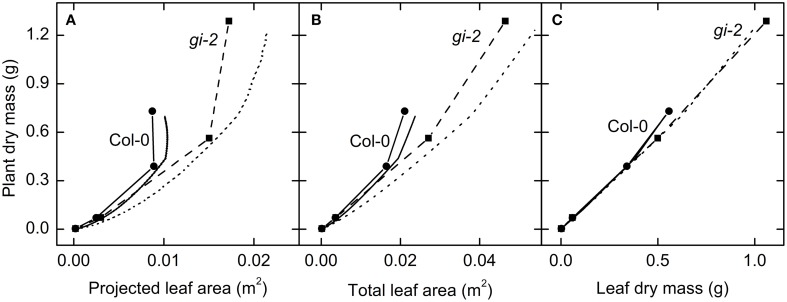
**The relationship between leaf area growth and plant growth over time**. Modeled data generated using partitioning coefficients in simulation 1 (Table 2) and measured data were used to determine the response of plant dry mass to variations in projected leaf area **(A)**, total leaf area **(B)**, and leaf mass **(C)**. Modeled data for Col-0 (solid lines) and *gi-2* (dotted lines) represent 1–90 DAS. Measured data for Col-0 (solid lines and filled circles) and *gi-2* (dashed lines and filled squares) was initially taken on 26 DAS for Col-0 and 25 DAS for *gi-2* followed by 44, 66, 86 DAS for both lines. Measured values represent the average of 10 measurements from 10 plants per line.

Plant mass increased with increasing projected and total leaf area and leaf mass (Figure 10). However, both modeled and measured data revealed that a linear relationship does not exist between projected leaf area, total leaf area, and total plant mass in both Col-0 and *gi-2* (Figures 10A,B). Data showed that above relationships altered from one growth phase to the next reflecting changes in C partitioning to different organs with time as shown in Table 2.

A linear relationship between leaf mass and plant mass could be seen during the vegetative phase of plant growth, when up to 88% of plant mass was composed of leaf mass (Figure 10C). At this stage leaf mass could provide a reasonably accurate estimation of plant mass. However, as the plants attained reproductive maturity, more C was allocated to inflorescence growth which disrupted this linear relationship such that leaf mass tended to largely underestimated plant mass (Figure 10C).

**Figure 11:**
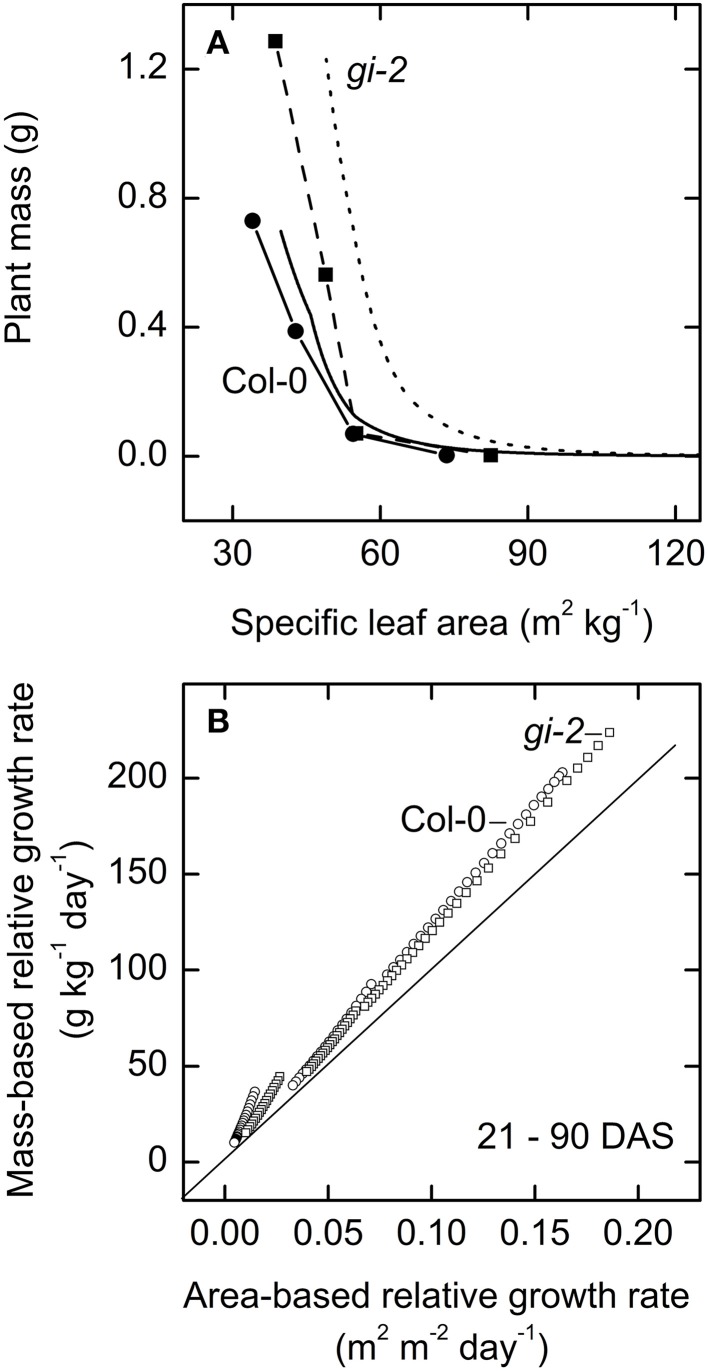
**The relationship between area-based relative growth rate and-mass based relative growth rate over time**. Modeled data generated using partitioning coefficients in simulation 1 (Table 2) and measured data were used to determine the relationship between plant mass and specific leaf area **(A)**, and to plot the relationship between mass-based relative growth rate and area-based relative growth rate **(B)**. In **(A)** modeled data for Col-0 (solid line) and *gi-2* (dotted line) represent 5–90 DAS and measured data for Col-0 (solid line and filled circles) and *gi-2* (dashed line and filled squares) was initially taken on 26 DAS for Col-0 and 25 DAS for *gi-2* followed by 44, 66, 86 DAS for both lines. Measured values represent the average of 10 measurements from 10 plants per line. In **(B)** modeled data for Col-0 (open circles) and *gi-2* (open squares) represent 21–90 DAS.

Both modeled and measured data revealed the dynamic relationship between specific leaf area and plant mass. While plant mass increased with plant age, specific leaf area decreased in both lines (Figure 11A). However, maintenance of higher specific leaf area seemed to yield greater plant biomass as seen in *gi-2* suggesting that higher specific leaf area may have been advantageous for *gi-2* under the low light conditions under which the plants were grown during the current experiment.

Modeled data indicated that the relationship between area-based relative growth rate and the mass-based relative growth rate altered from one growth phase to another based on the corresponding changes in C partitioning (Figure 11B, Table 2). Modeled data also showed that area-based relative growth rate tend to considerably underestimate mass-based relative growth rate during all growth phases.

## Discussion

### Key findings of the study

#### Leaf area is not a reliable tool to predict plant growth

The purpose of this study was to relate leaf area which is used as a tool in high-throughput-screening of plants with enhanced biomass or RGR_M_. The model was developed for *Arabidopsis* because it is a herbaceous annual plant which therefore provides a more simplistic system of photosynthetic C assimilation, partitioning and allocation. 88% of plant biomass at the end of the vegetative phase was leaf biomass in the *Arabidopsis* model system used in the present study. Thus, a near linear relationship between leaf mass and plant biomass existed during the vegetative phase. However, the model revealed that the relationship between projected and total leaf area with plant biomass and between area-based and mass-based relative growth rate was non-linear and inconsistent, which could be attributed to changes that occurred in C partitioning to various organs during transition from one growth phase to another. Thus, plant biomass can vary from leaf area measurements. Therefore, leaf area is only partly predictive of overall growth. Growth is more directly related to specific leaf area than to area-based photosynthetic rate even though photosynthesis is the source of all C used for growth.

#### Small changes in C partitioning, especially to leaf thickening and leaf area growth, can contribute to significant changes in plant growth

Mutant line *gi-2* grew larger than Col-0 both in terms of leaf area and plant mass despite greater area-based photosynthetic rates in Col-0. The *Arabidopsis* Leaf Area Growth Model developed during the present study was used as a tool to understand and explain the observed differences in growth in Col-0 and *gi-2* based on differences in C partitioning. Four important conclusions regarding partitioning of assimilated C and its contribution to plant growth could be made based on modeled growth for Col-0 and *gi-2*.

Firstly, overall plant growth in terms of leaf area growth and plant biomass is highly sensitive to the amount of C partitioned to leaf thickening. The model showed that maximizing leaf area growth by small reductions in C partitioning to leaf thickening with subsequent increments to leaf area growth, during early stages of vegetative growth has profoundly positive effects on leaf area, leaf mass, and plant biomass as seen in *gi-2*. Furthermore, mutant line *gi-2* continued to partition a greater proportion of NAR allocated to leaf growth to leaf area growth, continuously throughout its life cycle, which may have further enabled it to maintain larger leaf area and enhance total assimilated C as a consequence.

Leaf thickening has been attributed to longer palisade cells or extra number of cell layers and hence can increase the capacity for area-based photosynthesis (Pons and Pearcy, 1994; Mitchell et al., 1999; Evans and Pooter, 2001; Lambers et al., 2008). Partitioning between leaf thickening and area growth is considered a trade-off between the capacities for area based photosynthesis and light capture (White and Montes, 2005; Jullien et al., 2009). Increased C partitioning to leaf area growth enhances SLA. SLA has been shown to be determined by genetic and environmental factors and also by leaf and plant age and is a key parameter which contributes to morphological plasticity (White and Montes, 2005; White and Scott, 2006; Jullien et al., 2009; Karavin, 2013). For example, SLA has been shown to decrease with increasing plant demand for C (Jullien et al., 2009). It has been shown that plants grown under shade, produce leaves with a higher SLA or lower leaf mass area and allocate more nitrogen in leaves to light harvesting, thereby optimizing light interception and C assimilation per unit biomass of leaf (Evans and Pooter, 2001; Lambers et al., 2008). Also, previous studies have shown that growth is determined more by SLA than by area-based photosynthesis, especially under lower irradiances (Evans and Pooter, 2001; Lambers et al., 2008). During the present study, the modeled and measured data indicated an ontogenic decrease in SLA; however, under the low light conditions *gi-2* is capable of maintaining a greater specific leaf area and produce greater overall plant biomass.

At higher light levels there may be an advantage to thicker leaves that can take advantage of the extra light but for the typical growth conditions for *Arabidopsis* leaf, thickening diverts resources from area growth, increases future maintenance respiration cost, and increases the cost of adding new (thicker) leaf area (Pons and Pearcy, 1994; Mitchell et al., 1999; Evans and Pooter, 2001; Lambers et al., 2008). Thick leaves are commonly found in sun habitats and thin leaves in shade habitats (Pons and Pearcy, 1994; Mitchell et al., 1999; Evans and Pooter, 2001; Lambers et al., 2008). There are long-distance signals that may be involved in causing leaves to grow thicker (Ferjani et al., 2008) and it is known that leaf thickness responds to the total number of photons received in a day, not the peak irradiance (Chabot et al., 1979). For example, plants grown under shade conditions have been shown to have a higher specific leaf area and exhibit lower respiratory rates per unit area, which helps the plant to maximize C balance and compensate for reduced photosynthetic rates (Pons and Pearcy, 1994; Lambers et al., 2008). Upper canopy leaves are thicker and have correspondingly greater area-based leaf respiration rates (Mitchell et al., 1999). In general, the respiratory rates per unit area were lower in *gi-2* compared to Col-0. However, in the present model, Col-0 partitioned less available C to maintenance respiration than *gi-2* during early stages of growth. Lower maintenance respiratory costs in *gi-2* than in Col-0 became more apparent during the later stages of development, which may be as a result of reduced leaf thickness in *gi-2*. Thus, *gi-2* provides an excellent example of trade-off between increasing the capacity for area based photosynthesis by leaf thickening or light capture by area growth; the latter proving to be more crucial in enhancing leaf area growth, leaf mass, and subsequently plant mass in *gi-2*.

Many studies have been carried out to investigate mechanisms through which crop yields can be increased and the key mechanisms include: (1) enhancement of light interception by leaf canopy, (2) conversion of light energy to assimilated C, and (3) partitioning of assimilated C to harvested organs (Gifford et al., 1984; Koester et al., 2014). For example, faster canopy establishment, reduced lodging and longer growing periods have improved light interception in soybean leading to enhanced crop productivity (Koester et al., 2014). The *Arabidopsis* Leaf Area Growth Model indicates that enhanced partitioning to leaf area growth vs. leaf thickening enables plants to establish a canopy with a larger effective leaf area faster thereby enhancing light interception under low light conditions leading to greater NAR and yield. Many crop plants including soybean have been bred toward obtaining a greater harvest index (Gifford et al., 1984; Koester et al., 2014). The *Arabidopsis* Leaf Area Growth Model suggests that selecting plants with greater SLA or lower leaf mass area may allow selection of plants which can produce a greater harvest index under lower irradiances.

Secondly, based on model outputs, it seems enhanced C partitioning to leaf area growth can compensate for lack of C partitioned to inflorescence growth in plants with a shorter reproductive phase. In *gi-2*, the amount of C partitioned to inflorescence growth was considerably less than that partitioned to leaf area growth during all growth phases of the life cycle and the length of the reproductive phase did not seem to affect overall mass of the inflorescence nor plant biomass. However, because much more C was available, a smaller percentage partitioned to inflorescence growth was sufficient to allow inflorescence growth similar to Col-0.

Thirdly, the model revealed that although leaf area and plant growth was highly responsive to variations in C partitioned to leaf area growth and thickening, it was insensitive to C partitioning to starch or sucrose. Under normal conditions, starch stored during the day is mobilized at a nearly constant rate during the night allowing sufficient levels of sucrose for growth and maintenance processes at night (Pal et al., 2013; Pilkington et al., 2015). Carbon available for growth is the excess C after expenditure in maintenance respiration (Pilkington et al., 2015) and growth rates during the day and at night have been shown to positively correlate with the amount of sucrose available in the *Arabidopsis* rosette (Sulpice et al., 2009, 2014). Studies on diurnal patterns of protein synthesis and polysome loading have revealed that the rates of polysome loading is reduced at night and that it correlates with availability of sucrose in leaves (Pal et al., 2013). As protein synthesis is a major contributor to growth, lower polysome loading indicates reduced growth rate in leaves at night, which probably compensates for reduced C availability at night from starch degradation.

Finally, the *Arabidopsis* Leaf Area Growth Model revealed that leaf area growth and plant growth is more sensitive to changes in C partitioning to leaf thickening and area growth than to changes to photosynthesis. Thus, although plant growth depends on photosynthetic C, how much the plant grows depends on the dynamics of C partitioning, especially on the amount of C partitioned to leaf area growth and leaf thickening. Thus, C partitioning to leaf area growth and leaf thickening is a major mechanism through which photosynthesis drives plant growth.

### The *Arabidopsis* Leaf Area Growth Model is an effective tool to study how changes in C partitioning affects leaf area and plant growth

Most growth models have studied C partitioning to starch and sucrose and to leaf, root and inflorescence/stem growth (Figure 1) (Bidel et al., 2000; Mündermann et al., 2005; Letort et al., 2006; Rasse and Tocquin, 2006; Asl et al., 2011; Grossman et al., 2011; Tessmer et al., 2013; Marin and Jones, 2014). However, based on our knowledge, partitioning of net assimilated C to leaf thickening and its overall effects on area growth and whole plant growth has not been modeled. The *Arabidopsis* Leaf Area Growth Model successfully incorporated partitioning of C at all levels so that the impact on leaf area growth and subsequently plant biomass gain due to variations in C partitioning at each level could be simulated.

Most existing growth models do not account for root growth (Grossman et al., 2011). The *Arabidopsis* Leaf Area Growth Model is capable of demonstrating the effects of variations in C partitioning to root growth on leaf growth. The model also demonstrated variations in C partitioning to plant organs with plant age. As a result, it could reveal how the plant manages its limited reserves to prioritize growth of different organs at different growth phases thereby optimizing plant growth. As plants were grown hydroponically an accurate estimate of root mass was possible.

Importantly, not only does the *Arabidopsis* Leaf Area Growth Model allow testing of the effect of variation in C partitioning to leaf area growth and thickening, this can be varied during different growth phases of the plant. The model can predict the impact of C partitioning to leaf thickening vs. area growth and its broader effects on overall leaf area, total plant mass, RGR_S_ and RGR_M_.

The leaf area capable of light interception or the horizontal projection of the leaf area is defined as the effective leaf surface area (Honda and Fisher, 1978). The effective leaf area is often lower than the total leaf area as a result of branching patterns, multilayered leaf arrangement leading to leaf overlap and also leaf curling. A special feature of the *Arabidopsis* Leaf Area Growth Model is the built-in correction for leaf area overlap. This prevents overestimation of the amount of assimilated C. However, an assumption of 10% contribution of overlapping leaves toward total photosynthesis was required in order to assure that modeled leaf mass at 86 DAS matched to measured leaf mass. Nevertheless, taking into account the progression of leaf overlap with plant age provided a more physiologically realistic modeling of available C for leaf and plant growth. As maximizing the effective leaf area as much as possible enhances light interception and productivity in plants, it is possible to test the effect of reduced leaf overlap on C assimilation, leaf and plant growth in *Arabidopsis* mutant lines with different rosette and leaf architecture as was performed in the present study.

Since the *Arabidopsis* Leaf Area Growth Model simulates plant growth from seed, the model provides an opportunity to test maternal effects such as the impact of seed weight, rate of storage reserve mobilization, germination rate, initial leaf area or cotyledon area on leaf and plant growth; this feature is lacking in most models mentioned above.

The *Arabidopsis* Leaf Area Growth Model assumes that growth during night-time and day-time is equal as long as C availability and partitioning are optimum. Some studies show that as a result of night-time partial stomatal closure transpiration rates at night are 5–30% of that during the day which may have a negative impact on the supply of minerals such as NO^−^_3_ to the root zone, on the water status of the plant, and growth (Snyder et al., 2003, 2008; Caird et al., 2007). The magnitude of night-time reduction in growth under these circumstances is not known. Recent, studies on soybean under unstressed conditions show that leaf growth show consistent diel growth patterns and that nighttime RGR may or may not be greater than daytime RGR (Walter et al., 2009; Friedli and Walter, 2015). It has been shown that under adequate water availability, the magnitude of night-time transpiration rate does not affect leaf nutrient content or rosette dry weight in *Arabidopsis* (Christman et al., 2009). However, nutrient content and growth at night may be more sensitive under drought stress (Christman et al., 2009; Friedli and Walter, 2015). Expansive growth may require a minimum turgor pressure and may be increased at night depending on water relations. The *Arabidopsis* Leaf Area Growth Model can be modified to introduce a night-time growth penalty to represent specific circumstances such as water stress conditions under which stomatal closure can result in reduced night-time transpiration rates, N, and water availability.

In summary, the *Arabidopsis* Leaf Area Growth Model designed and developed during the present study is an effective tool to study how changes in C partition to maintenance and growth processes, especially to leaf area growth and leaf thickening affects plant growth. The model revealed that leaf area measurements may not represent plant growth accurately as the relationship between leaf area and plant biomass was non-linear and variable depending on C partitioning. The study revealed that while photosynthesis may drive plant growth, large changes in growth can occur as a result of small changes in partitioning of assimilated C to growth, especially to leaf area growth and thickening. The model provided several mechanisms through which to optimize leaf area growth and plant growth: (1) increased partitioning of NAR to leaf growth throughout the life cycle and especially during the early vegetative phase; (2) increased partitioning of NAR available for leaf growth to leaf area growth and reduced partitioning to leaf thickening throughout the life cycle and especially during the early vegetative phase; (3) maintenance of increased specific leaf area throughout the life span; (4) reduction in leaf overlap via longer petioles.

### Conflict of interest statement

The authors declare that the research was conducted in the absence of any commercial or financial relationships that could be construed as a potential conflict of interest.
