# Supplementary material for: The relationship between leaf area growth and biomass accumulation in Arabidopsis thaliana
Source: Front Plant Sci. 2015 Apr 9;6:167. doi: 10.3389/fpls.2015.00167 (PMC4391269; doi:10.3389/fpls.2015.00167)
Supplement: Supplementary file 1 [file Table1.DOCX]

**Supplementary Table 1: Constraints used for the multi-objective optimization of the *Arabidopsis* Leaf Area Growth Model.** Following constraints were selected based on plant growth measurements, literature survey, and expert opinion to constrain C partitioning to leaves, roots and inflorescence during multi-objective optimization of the *Arabidopsis* Leaf Area Growth Model. The symbols ι, ρ, ^s^λ, and ^t^λ stand for partition coefficients of C partitioning to the inflorescence, roots, leaf area growth and leaf thickening, respectively.

| Partitioning coefficients | Growth phase | | | |
| --- | --- | --- | --- | --- |
|  | Germination | Early vegetative | Late vegetative | Reproductive |
|  |  |  |  |  |
| ι | <0.001 | <0.001 | - | - |
| ρ | <0.001 | 0.05-0.20 | 0.05-0.20 | 0.05-0.20 |
| ^s^λ | >0.999 | - | - | - |
| ^t^λ | - | - | - | - |
