# Supplementary material for: The relationship between leaf area growth and biomass accumulation in Arabidopsis thaliana
Source: Front Plant Sci. 2015 Apr 9;6:167. doi: 10.3389/fpls.2015.00167 (PMC4391269; doi:10.3389/fpls.2015.00167)
Supplement: Supplementary file 3 [file Presentation1.PPTX]

## Slide 1
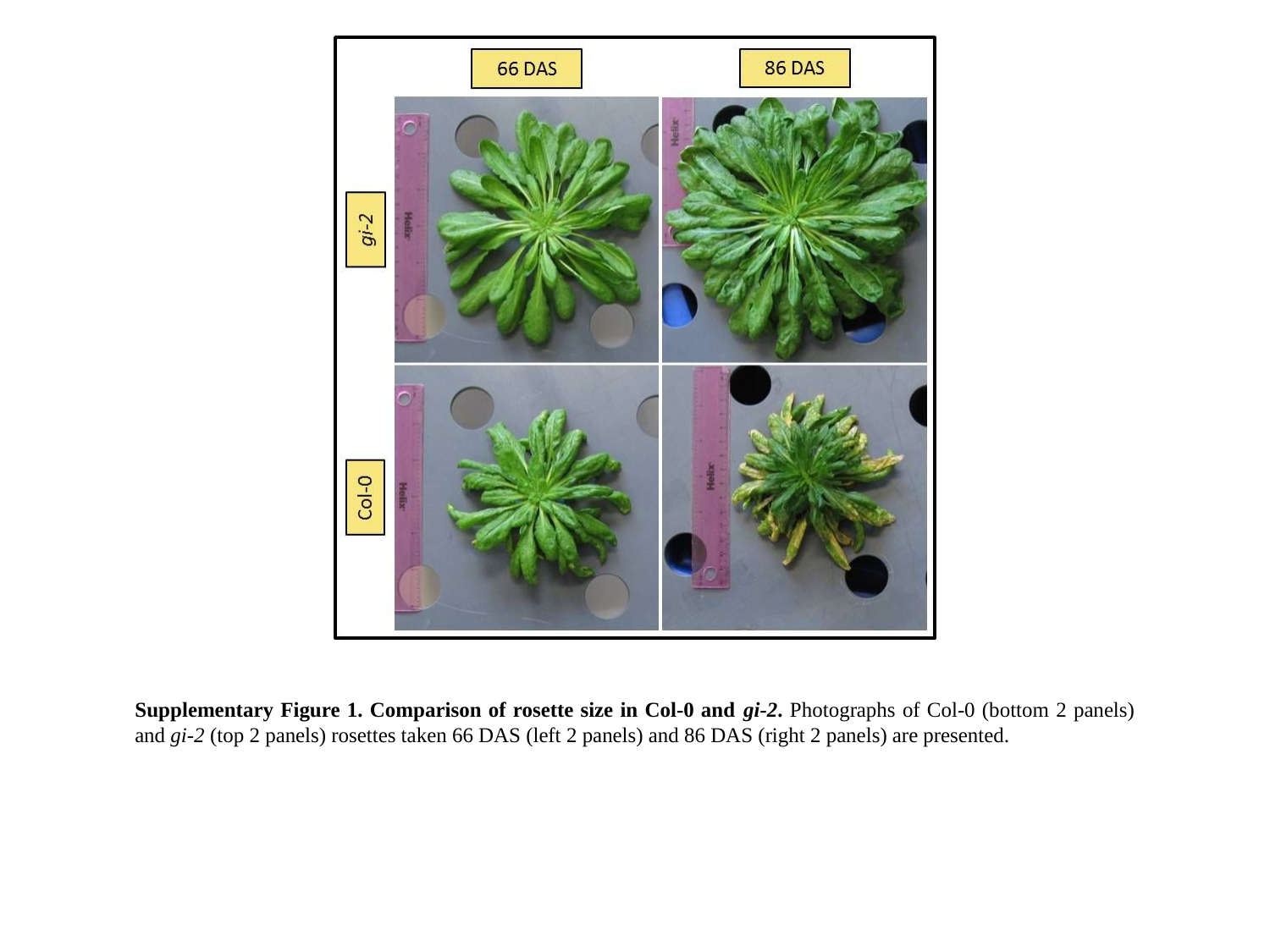

Supplementary Figure 1. Comparison of rosette size in Col-0 and gi-2. Photographs of Col-0 (bottom 2 panels) and gi-2 (top 2 panels) rosettes taken 66 DAS (left 2 panels) and 86 DAS (right 2 panels) are presented.

## Slide 2
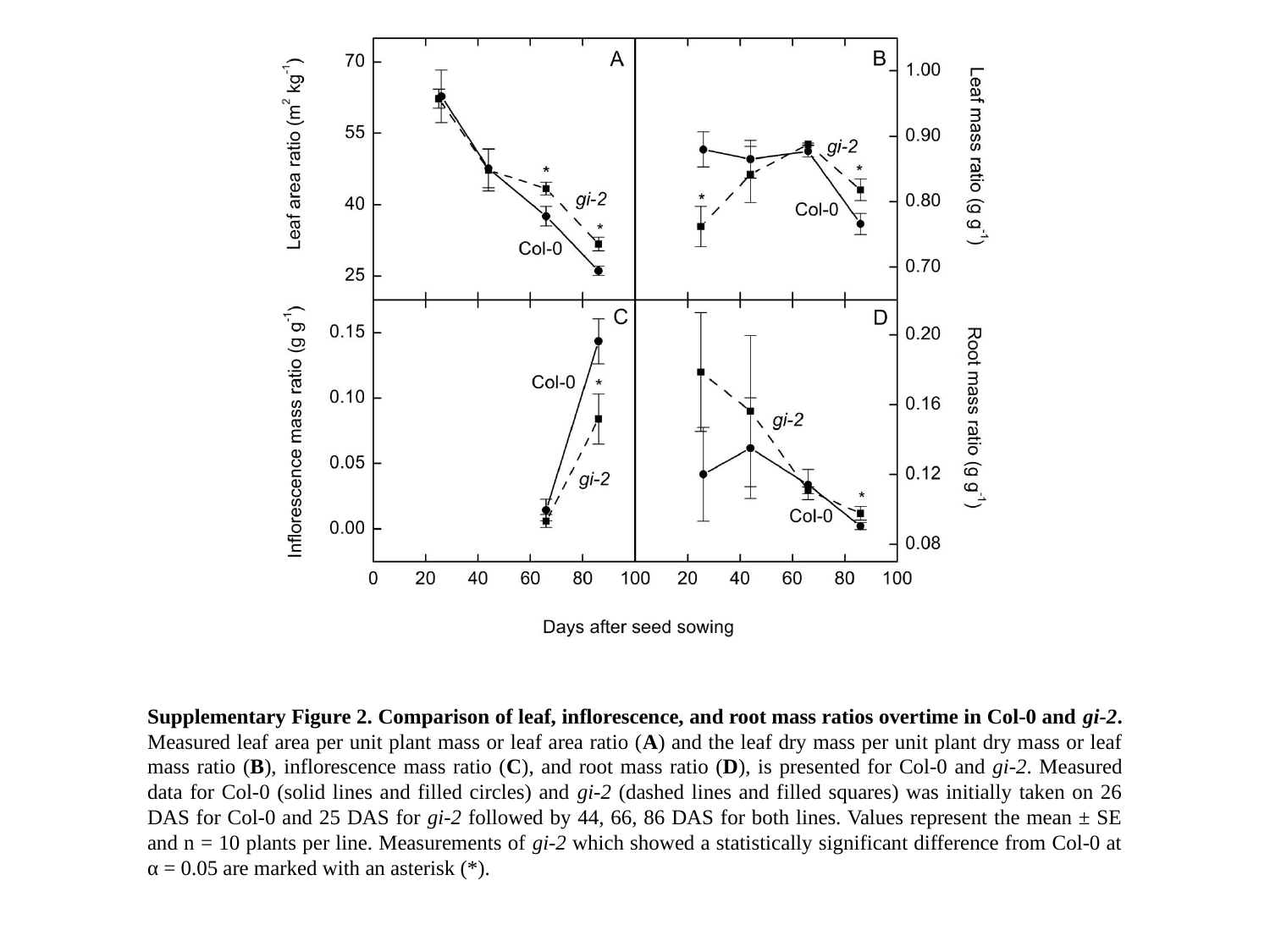

Supplementary Figure 2. Comparison of leaf, inflorescence, and root mass ratios overtime in Col-0 and gi-2. Measured leaf area per unit plant mass or leaf area ratio (A) and the leaf dry mass per unit plant dry mass or leaf mass ratio (B), inflorescence mass ratio (C), and root mass ratio (D), is presented for Col-0 and gi-2. Measured data for Col-0 (solid lines and filled circles) and gi-2 (dashed lines and filled squares) was initially taken on 26 DAS for Col-0 and 25 DAS for gi-2 followed by 44, 66, 86 DAS for both lines. Values represent the mean ± SE and n = 10 plants per line. Measurements of gi-2 which showed a statistically significant difference from Col-0 at α = 0.05 are marked with an asterisk (*).

## Slide 3
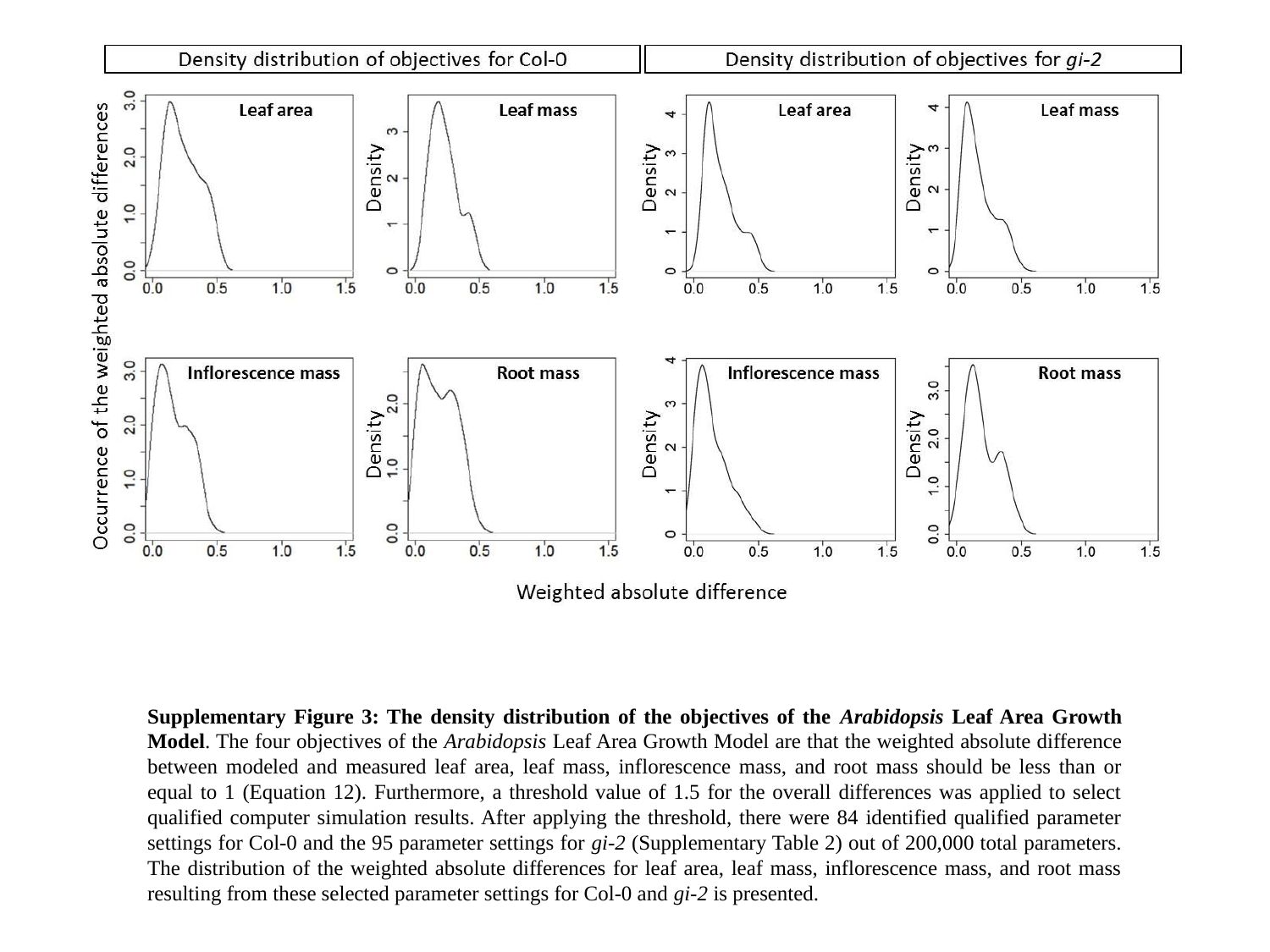

Supplementary Figure 3: The density distribution of the objectives of the Arabidopsis Leaf Area Growth Model. The four objectives of the Arabidopsis Leaf Area Growth Model are that the weighted absolute difference between modeled and measured leaf area, leaf mass, inflorescence mass, and root mass should be less than or equal to 1 (Equation 12). Furthermore, a threshold value of 1.5 for the overall differences was applied to select qualified computer simulation results. After applying the threshold, there were 84 identified qualified parameter settings for Col-0 and the 95 parameter settings for gi-2 (Supplementary Table 2) out of 200,000 total parameters. The distribution of the weighted absolute differences for leaf area, leaf mass, inflorescence mass, and root mass resulting from these selected parameter settings for Col-0 and gi-2 is presented.

## Slide 4
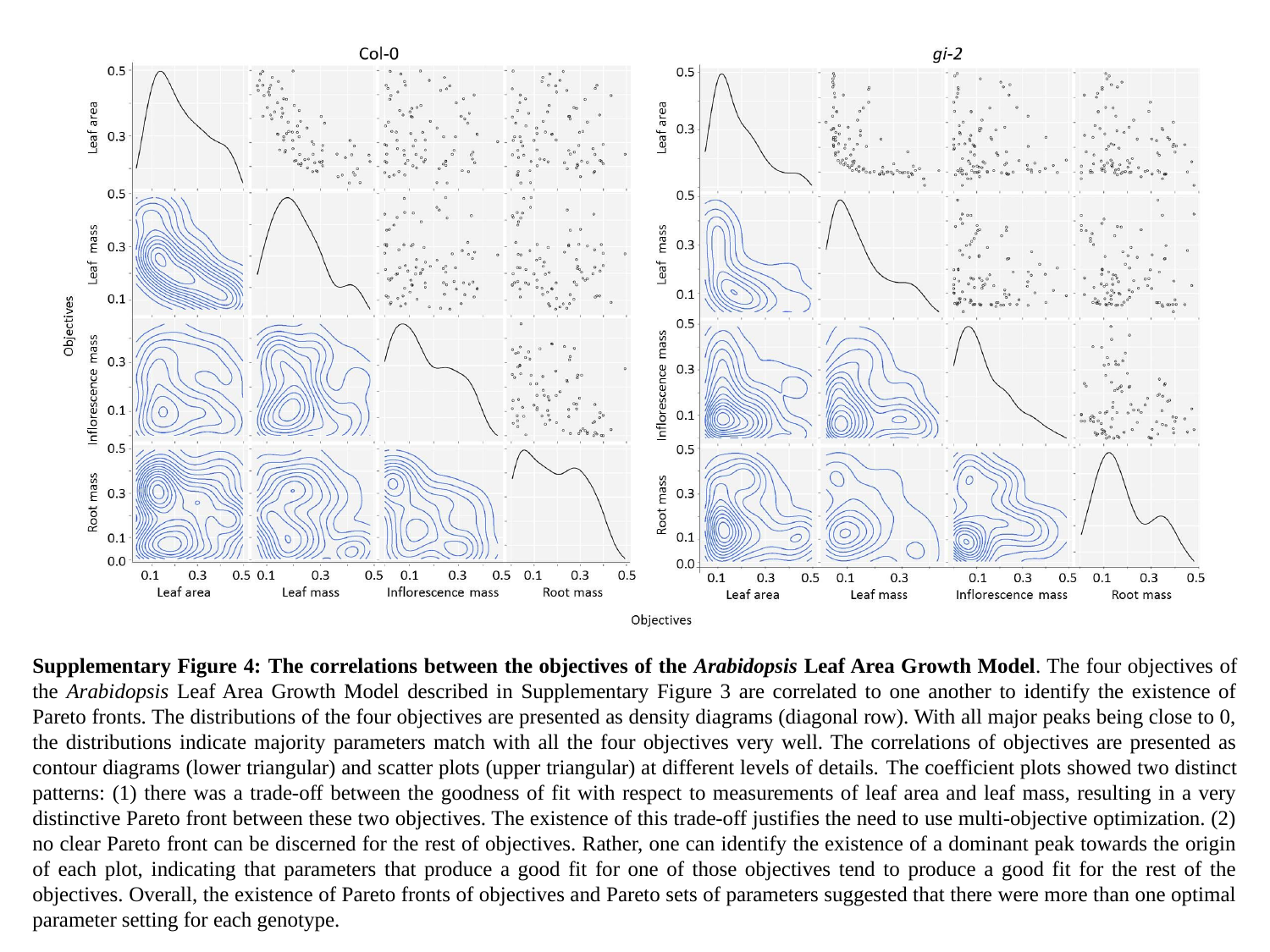

Supplementary Figure 4: The correlations between the objectives of the Arabidopsis Leaf Area Growth Model. The four objectives of the Arabidopsis Leaf Area Growth Model described in Supplementary Figure 3 are correlated to one another to identify the existence of Pareto fronts. The distributions of the four objectives are presented as density diagrams (diagonal row). With all major peaks being close to 0, the distributions indicate majority parameters match with all the four objectives very well. The correlations of objectives are presented as contour diagrams (lower triangular) and scatter plots (upper triangular) at different levels of details. The coefficient plots showed two distinct patterns: (1) there was a trade-off between the goodness of fit with respect to measurements of leaf area and leaf mass, resulting in a very distinctive Pareto front between these two objectives. The existence of this trade-off justifies the need to use multi-objective optimization. (2) no clear Pareto front can be discerned for the rest of objectives. Rather, one can identify the existence of a dominant peak towards the origin of each plot, indicating that parameters that produce a good fit for one of those objectives tend to produce a good fit for the rest of the objectives. Overall, the existence of Pareto fronts of objectives and Pareto sets of parameters suggested that there were more than one optimal parameter setting for each genotype.

## Slide 5
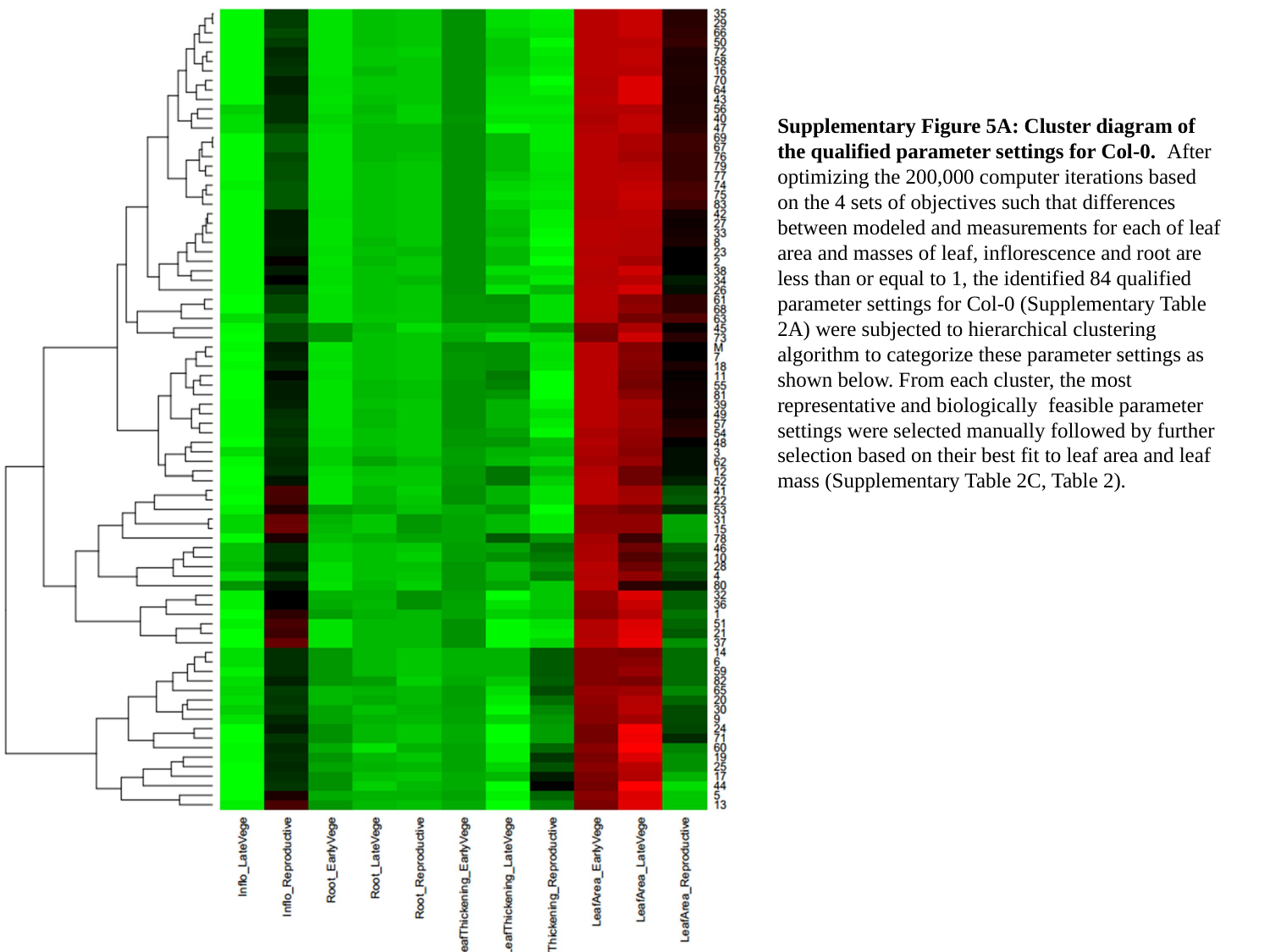

Supplementary Figure 5A: Cluster diagram of the qualified parameter settings for Col-0. After optimizing the 200,000 computer iterations based on the 4 sets of objectives such that differences between modeled and measurements for each of leaf area and masses of leaf, inflorescence and root are less than or equal to 1, the identified 84 qualified parameter settings for Col-0 (Supplementary Table 2A) were subjected to hierarchical clustering algorithm to categorize these parameter settings as shown below. From each cluster, the most representative and biologically feasible parameter settings were selected manually followed by further selection based on their best fit to leaf area and leaf mass (Supplementary Table 2C, Table 2).

## Slide 6
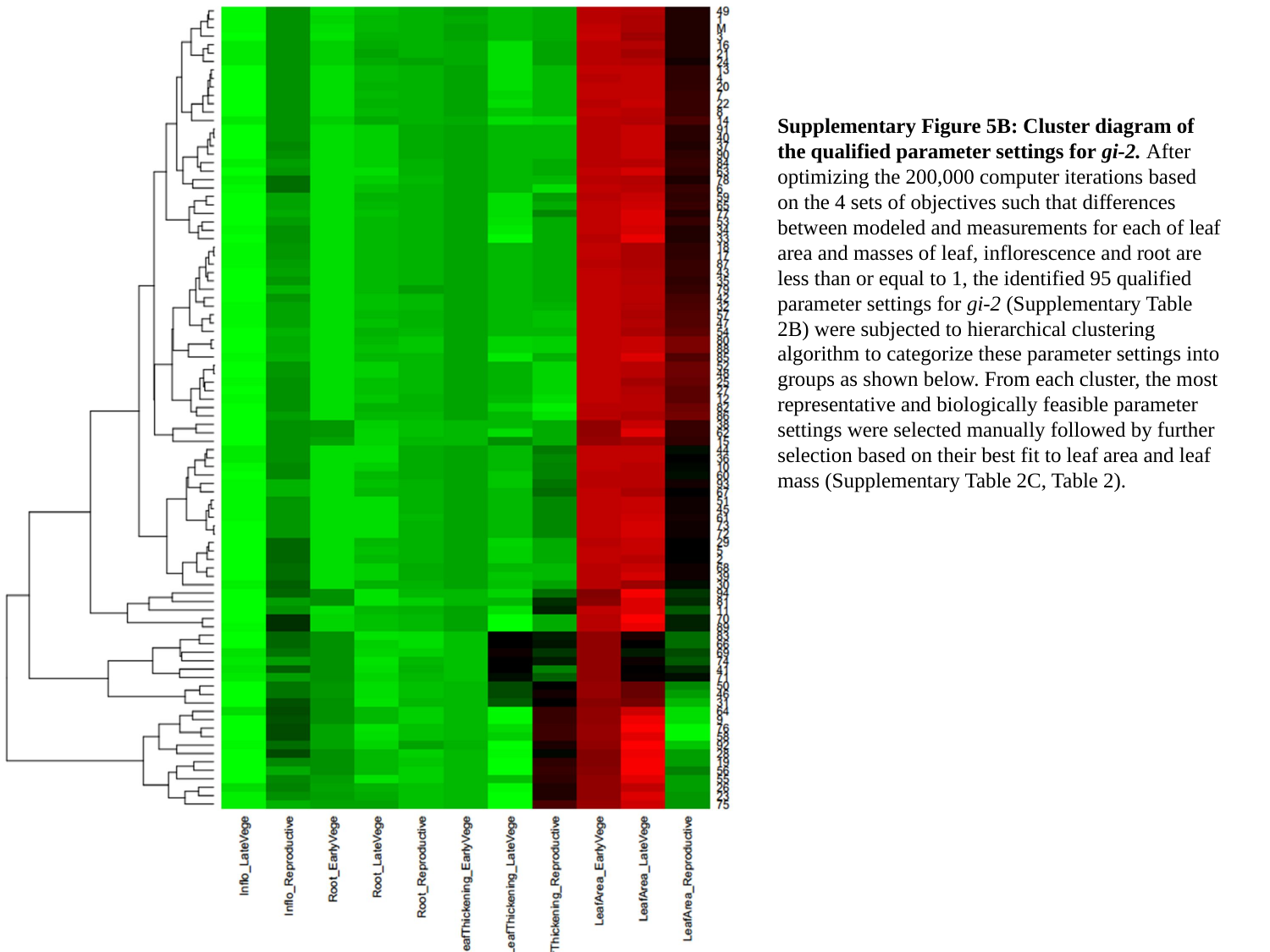

Supplementary Figure 5B: Cluster diagram of the qualified parameter settings for gi-2. After optimizing the 200,000 computer iterations based on the 4 sets of objectives such that differences between modeled and measurements for each of leaf area and masses of leaf, inflorescence and root are less than or equal to 1, the identified 95 qualified parameter settings for gi-2 (Supplementary Table 2B) were subjected to hierarchical clustering algorithm to categorize these parameter settings into groups as shown below. From each cluster, the most representative and biologically feasible parameter settings were selected manually followed by further selection based on their best fit to leaf area and leaf mass (Supplementary Table 2C, Table 2).

## Slide 7
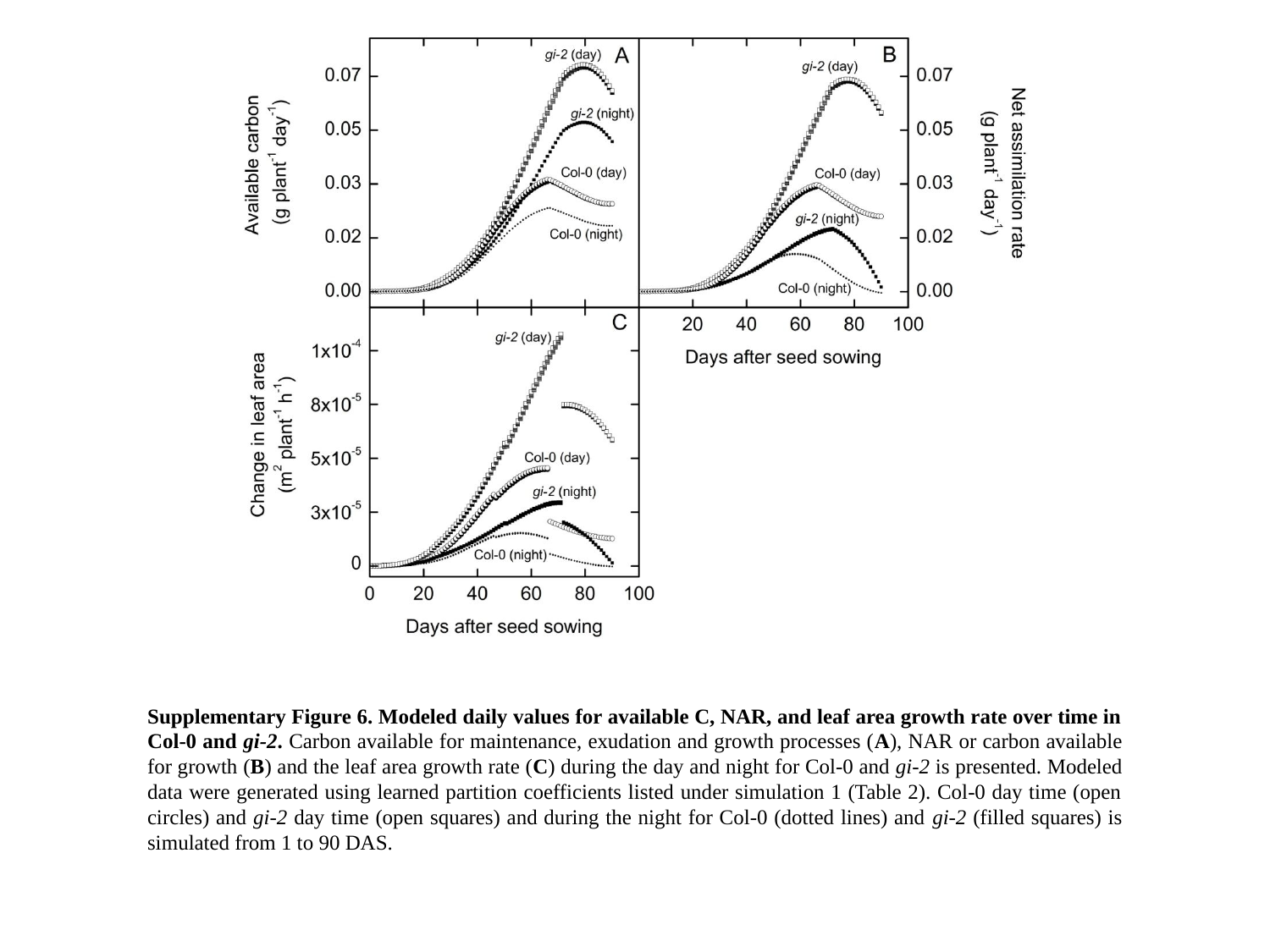

Supplementary Figure 6. Modeled daily values for available C, NAR, and leaf area growth rate over time in Col-0 and gi-2. Carbon available for maintenance, exudation and growth processes (A), NAR or carbon available for growth (B) and the leaf area growth rate (C) during the day and night for Col-0 and gi-2 is presented. Modeled data were generated using learned partition coefficients listed under simulation 1 (Table 2). Col-0 day time (open circles) and gi-2 day time (open squares) and during the night for Col-0 (dotted lines) and gi-2 (filled squares) is simulated from 1 to 90 DAS.
