# Supplementary material for: The relationship between leaf area growth and biomass accumulation in Arabidopsis thaliana
Source: Front Plant Sci. 2015 Apr 9;6:167. doi: 10.3389/fpls.2015.00167 (PMC4391269; doi:10.3389/fpls.2015.00167)
Supplement: Supplementary file 4 [file DataSheet1.ZIP › R_libs - parallization.zip!/R_libs/doParallel/doc/gettingstartedParallel.pdf]

Getting Started with doParallel and foreach

Steve Weston*and Rich Calaway
doc@revolutionanalytics.com

February 26, 2014

1 Introduction

The doParallel package is a "parallel backend" for the foreach package. It provides a mechanism needed to execute foreach loops in parallel. The foreach package must be used in conjunction with a package such as doParallel in order to execute code in parallel. The user must register a parallel backend to use, otherwise foreach will execute tasks sequentially, even when the %dopar% operator is used.[1]

The doParallel package acts as an interface between foreach and the parallel package of R 2.14.0 and later. The parallel package is essentially a merger of the multicore package, which was written by Simon Urbanek, and the snow package, which was written by Luke Tierney and others. The multicore functionality supports multiple workers only on those operating systems that support the fork system call; this excludes Windows. By default, doParallel uses multicore functionality on Unix-like systems and snow functionality on Windows. Note that the multicore functionality only runs tasks on a single computer, not a cluster of computers. However, you can use the snow functionality to execute on a cluster, using Unix-like operating systems, Windows, or even a combination. It is pointless to use doParallel and parallel on a machine with only one processor with a single core. To get a speed improvement, it must run on a machine with multiple processors, multiple cores, or both.

*Steve Weston wrote the original version of this vignette for the doMC package. Rich Calaway adapted the vignette for doParallel.

[1]foreach will issue a warning that it is running sequentially if no parallel backend has been registered. It will only issue this warning once, however.

2 A word of caution

Because the `parallel` package in `multicore` mode starts its workers using `fork` without doing a subsequent `exec`, it has some limitations. Some operations cannot be performed properly by forked processes. For example, connection objects very likely won't work. In some cases, this could cause an object to become corrupted, and the R session to crash.

3 Registering the `doParallel` parallel backend

To register `doParallel` to be used with `foreach`, you must call the `registerDoParallel` function. If you call this with no arguments, on Windows you will get three workers and on Unix-like systems you will get a number of workers equal to approximately half the number of cores on your system. You can also specify a cluster (as created by the `makeCluster` function) or a number of cores. The `cores` argument specifies the number of worker processes that `doParallel` will use to execute tasks, which will by default be equal to one-half the total number of cores on the machine. You don't need to specify a value for it, however. By default, `doParallel` will use the value of the "cores" option, as specified with the standard "options" function. If that isn't set, then `doParallel` will try to detect the number of cores, and use one-half that many workers.

Remember: unless `registerDoMC` is called, `foreach` will *not* run in parallel. Simply loading the `doParallel` package is not enough.

4 An example `doParallel` session

Before we go any further, let's load `doParallel`, register it, and use it with `foreach`. We will use `snow`-like functionality in this vignette, so we start by loading the package and starting a cluster:

```
> library(doParallel)
> cl <- makeCluster(2)
> registerDoParallel(cl)
> foreach(i=1:3) %dopar% sqrt(i)
```

```
[[1]]
[1] 1

[[2]]
[1] 1.414214
```

```
[[3]]
[1] 1.732051
```

To use `multicore`-like functionality, we would specify the number of cores to use instead (but note that on Windows, attempting to use more than one core with `parallel` results in an error):

```
library(doParallel}
registerDoParallel(cores=2)
foreach(i=1:3) %dopar% sqrt(i)
```

> Note well that this is *not* a practical use of `doParallel`. This is our "Hello, world" program for parallel computing. It tests that everything is installed and set up properly, but don't expect it to run faster than a sequential `for` loop, because it won't! `sqrt` executes far too quickly to be worth executing in parallel, even with a large number of iterations. With small tasks, the overhead of scheduling the task and returning the result can be greater than the time to execute the task itself, resulting in poor performance. In addition, this example doesn't make use of the vector capabilities of `sqrt`, which it must to get decent performance. This is just a test and a pedagogical example, *not* a benchmark.

But returning to the point of this example, you can see that it is very simple to load `doParallel` with all of its dependencies (`foreach`, `iterators`, `parallel`, etc), and to register it. For the rest of the R session, whenever you execute `foreach` with `%dopar%`, the tasks will be executed using `doParallel` and `parallel`. Note that you can register a different parallel backend later, or deregister `doParallel` by registering the sequential backend by calling the `registerDoSEQ` function.

5 A more serious example

Now that we've gotten our feet wet, let's do something a bit less trivial. One good example is bootstrapping. Let's see how long it takes to run 10,000 bootstrap iterations in parallel on 2 cores:

```
> x <- iris[which(iris[,5] != "setosa"), c(1,5)]
> trials <- 10000
> ptime <- system.time({
+   r <- foreach(icount(trials), .combine=cbind) %dopar% {
+     ind <- sample(100, 100, replace=TRUE)
+     result1 <- glm(x[ind,2]~x[ind,1], family=binomial(logit))
+     coefficients(result1)
+   }
+ })[3]
> ptime
```

```
elapsed
156.163
```

Using `doParallel` and `parallel` we were able to perform 10,000 bootstrap iterations in 156.163 seconds on 2 cores. By changing the `%dopar%` to `%do%`, we can run the same code sequentially to determine the performance improvement:

```
> stime <- system.time({
+   r <- foreach(icount(trials), .combine=cbind) %do% {
+     ind <- sample(100, 100, replace=TRUE)
+     result1 <- glm(x[ind,2]~x[ind,1], family=binomial(logit))
+     coefficients(result1)
+   }
+ })[3]
> stime
```

```
elapsed
213.528
```

The sequential version ran in 213.528 seconds, which means the speed up is about 1.4 on 2 workers.[2] Ideally, the speed up would be 2, but no multicore CPUs are ideal, and neither are the operating systems and software that run on them.

At any rate, this is a more realistic example that is worth executing in parallel. We do not explain what it's doing or how it works here. We just want to give you something more substantial than the `sqrt` example in case you want to run some benchmarks yourself. You can also run this example on a cluster by simply reregistering with a cluster object that specifies the nodes to use. (See the `makeCluster` help file for more details.)

6 Getting information about the parallel backend

To find out how many workers `foreach` is going to use, you can use the `getDoParWorkers` function:

```
> getDoParWorkers()
```

```
[1] 2
```

[2]If you build this vignette yourself, you can see how well this problem runs on your hardware. None of the times are hardcoded in this document. You can also run the same example which is in the examples directory of the `doParallel` distribution.

This is a useful sanity check that you're actually running in parallel. If you haven't registered a parallel backend, or if your machine only has one core, `getDoParWorkers` will return one. In either case, don't expect a speed improvement. `foreach` is clever, but it isn't magic.

The `getDoParWorkers` function is also useful when you want the number of tasks to be equal to the number of workers. You may want to pass this value to an iterator constructor, for example.

You can also get the name and version of the currently registered backend:

```
> getDoParName()
```

```
[1] "doParallelSNOW"
```

```
> getDoParVersion()
```

```
[1] "1.0.8"
```

This is mostly useful for documentation purposes, or for checking that you have the most recent version of `doParallel`.

7 Specifying multicore options

When using `multicore`-like functionality, the `doParallel` package allows you to specify various options when running `foreach` that are supported by the underlying `mclapply` function: "preschedule", "set.seed", "silent", and "cores". You can learn about these options from the `mclapply` man page. They are set using the `foreach .options.multicore` argument. Here's an example of how to do that:

```
mcoptions <- list(preschedule=FALSE, set.seed=FALSE)
foreach(i=1:3, .options.multicore=mcoptions) %dopar% sqrt(i)
```

The "cores" options allows you to temporarily override the number of workers to use for a single `foreach` operation. This is more convenient than having to re-register `doParallel`. Although if no value of "cores" was specified when `doParallel` was registered, you can also change this value dynamically using the `options` function:

```
options(cores=2)
getDoParWorkers()
options(cores=3)
getDoParWorkers()
```

If you did specify the number of cores when registering doParallel, the “cores” option is ignored:

```
registerDoParallel(4)
options(cores=2)
getDoParWorkers()
```

As you can see, there are a number of options for controlling the number of workers to use with parallel, but the default behaviour usually does what you want.

8 Stopping your cluster

If you are using snow-like functionality, you will want to stop your cluster when you are done using it. The doParallel package’s .onUnload function will do this automatically if the cluster was created automatically by registerDoParallel, but if you created the cluster manually you should stop it using the stopCluster function:

```
stopCluster(cl)
```

9 Conclusion

The doParallel and parallel packages provide a nice, efficient parallel programming platform for multiprocessor/multicore computers running operating systems such as Linux and Mac OS X. It is very easy to install, and very easy to use. In short order, an average R programmer can start executing parallel programs, without any previous experience in parallel computing.
