# Supplementary material for: The relationship between leaf area growth and biomass accumulation in Arabidopsis thaliana
Source: Front Plant Sci. 2015 Apr 9;6:167. doi: 10.3389/fpls.2015.00167 (PMC4391269; doi:10.3389/fpls.2015.00167)
Supplement: Supplementary file 4 [file DataSheet1.ZIP › R_libs - parallization.zip!/R_libs/doParallel/html/00Index.html]

R: Foreach parallel adaptor for the parallel package

# Foreach parallel adaptor for the parallel package

---

## Documentation for package ‘doParallel’ version 1.0.8

- DESCRIPTION file.
- User guides, package vignettes and other documentation.
- Code demos. Use demo() to run them.
- Package NEWS.

## Help Pages

|  |  |
| --- | --- |
| doParallel-package | The doParallel Package |
| doParallel | The doParallel Package |
| registerDoParallel | registerDoParallel |
| stopImplicitCluster | registerDoParallel |
