# Supplementary material for: The relationship between leaf area growth and biomass accumulation in Arabidopsis thaliana
Source: Front Plant Sci. 2015 Apr 9;6:167. doi: 10.3389/fpls.2015.00167 (PMC4391269; doi:10.3389/fpls.2015.00167)
Supplement: Supplementary file 4 [file DataSheet1.ZIP › R_libs - parallization.zip!/R_libs/mco/html/00Index.html]

R: Multiple criteria optimization algorithms and related functions

# Multiple criteria optimization algorithms and related functions

---

## Documentation for package ‘mco’ version 1.0-14

- DESCRIPTION file.

## Help Pages

|  |  |
| --- | --- |
| belegundu | MCO test problems |
| belegundu.constr | MCO test problems |
| binh1 | MCO test problems |
| binh2 | MCO test problems |
| binh2.constr | MCO test problems |
| binh3 | MCO test problems |
| deb3 | MCO test problems |
| dominatedHypervolume | Quality measures for MCO solutions |
| epsilonIndicator | Quality measures for MCO solutions |
| fonseca1 | MCO test problems |
| fonseca2 | MCO test problems |
| generalizedSpread | Quality measures for MCO solutions |
| generationalDistance | Quality measures for MCO solutions |
| gianna | MCO test problems |
| hanne1 | MCO test problems |
| hanne1.constr | MCO test problems |
| hanne2 | MCO test problems |
| hanne2.constr | MCO test problems |
| hanne3 | MCO test problems |
| hanne3.constr | MCO test problems |
| hanne4 | MCO test problems |
| hanne4.constr | MCO test problems |
| hanne5 | MCO test problems |
| hanne5.constr | MCO test problems |
| jimenez | MCO test problems |
| jimenez.constr | MCO test problems |
| normalizeFront | Normalize a pareto front |
| nsga2 | NSGA II MOEA |
| paretoFilter | Pareto Front and pareto set accessor |
| paretoFront | Pareto Front and pareto set accessor |
| paretoSet | Pareto Front and pareto set accessor |
| vnt | MCO test problems |
| zdt1 | MCO test problems |
| zdt2 | MCO test problems |
| zdt3 | MCO test problems |
