# Supplementary material for: The relationship between leaf area growth and biomass accumulation in Arabidopsis thaliana
Source: Front Plant Sci. 2015 Apr 9;6:167. doi: 10.3389/fpls.2015.00167 (PMC4391269; doi:10.3389/fpls.2015.00167)
Supplement: Supplementary file 4 [file DataSheet1.ZIP › R_libs - parallization.zip!/R_libs/randtoolbox/doc/fullpres.pdf]

# A note on random number generation

Christophe Dutang and Diethelm Wuertz

September 2009

*"Nothing in Nature is random...*
*a thing appears random only through*
*the incompleteness of our knowledge."*
Spinoza, Ethics I[1].

# 1 Introduction

Random simulation has long been a very popular and well studied field of mathematics. There exists a wide range of applications in biology, finance, insurance, physics and many others. So simulations of random numbers are crucial. In this note, we describe the most random number algorithms

Let us recall the only things, that are truly random, are the measurement of physical phenomena such as thermal noises of semiconductor chips or radioactive sources[2].

The only way to simulate some randomness on computers are carried out by deterministic algorithms. Excluding true randomness[3], there are two kinds random generation: pseudo and quasi random number generators.

The package **randtoolbox** provides R functions for pseudo and quasi random number generations, as well as statistical tests to quantify the quality of generated random numbers.

# 2 Overview of random generation algoritms

In this section, we present first the pseudo random number generation and second the quasi random number generation. By "random numbers", we mean random variates of the uniform $\mathcal{U}(0,1)$ distribution. More complex distributions can be generated with uniform variates and rejection or inversion methods. Pseudo random number generation aims to seem random whereas quasi random number generation aims to be deterministic but well equidistributed.

Those familiars with algorithms such as linear congruential generation, Mersenne-Twister type algorithms, and low discrepancy sequences should go directly to the next section.

## 2.1 Pseudo random generation

At the beginning of the nineties, there was no state-of-the-art algorithms to generate pseudo random numbers. And the article of Park & Miller (1988) entitled *Random generators: good ones are hard to find* is a clear proof.

Despite this fact, most users thought the `rand` function they used was good, because of a short period and a term to term dependence. But in 1998, Japenese mathematicians Matsumoto and Nishimura invents the first algorithm whose period ($2^{19937}-1$) exceeds the number of electron spin changes since the creation of the Universe ($10^{6000}$ against $10^{120}$). It was a **big** breakthrough.

As described in L'Ecuyer (1990), a (pseudo) random number generator (RNG) is defined by a structure $(S, \mu, f, U, g)$ where

- $S$ a finite set of *states*,
- $\mu$ a probability distribution on $S$, called the *initial distribution*,
- a *transition function* $f : S \mapsto S$,
- a finite set of *output* symbols $U$,
- an *output function* $g : S \mapsto U$.

Then the generation of random numbers is as follows:

1. generate the initial state (called the *seed*) $s_0$

[1] quote taken from Niederreiter (1978).
[2] for more details go to `http://www.random.org/randomness/`.
[3] For true random number generation on R, use the **random** package of Eddelbuettel (2007).

according to $\mu$ and compute $u_0 = g(s_0)$,
2. iterate for $i = 1, \dots,$ $s_i = f(s_{i-1})$ and $u_i = g(s_i)$.

Generally, the seed $s_0$ is determined using the clock machine, and so the random variates $u_0, \dots, u_n, \dots$ seems "real" i.i.d. uniform random variates. The period of a RNG, a key characteristic, is the smallest integer $p \in \mathbb{N}$, such that $\forall n \in \mathbb{N}, s_{p+n} = s_n$.

### 2.1.1 Linear congruential generators

There are many families of RNGs : linear congruential, multiple recursive,. . . and "computer operation" algorithms. Linear congruential generators have a *transfer function* of the following type

$$f(x) = (ax + c) \mod m^1,$$

where $a$ is the multiplier, $c$ the increment and $m$ the modulus and $x, a, c, m \in \mathbb{N}$ (i.e. $S$ is the set of (positive) integers). $f$ is such that

$$x_n = (ax_{n-1} + c) \mod m.$$

Typically, $c$ and $m$ are chosen to be relatively prime and $a$ such that $\forall x \in \mathbb{N}, ax \mod m \neq 0$. The cycle length of linear congruential generators will never exceed modulus $m$, but can maximised with the three following conditions

- increment $c$ is relatively prime to $m$,
- $a-1$ is a multiple of every prime dividing $m$,
- $a-1$ is a multiple of 4 when $m$ is a multiple of 4,

see Knuth (2002) for a proof.

When $c = 0$, we have the special case of Park-Miller algorithm or Lehmer algorithm (see Park & Miller (1988)). Let us note that the $n+j$th term can be easily derived from the $n$th term with $a$ puts to $a^j \mod m$ (still when $c = 0$).

[1]this representation could be easily generalized for matrix, see L'Ecuyer (1990).

Finally, we generally use one of the three types of *output function*:

- $g : \mathbb{N} \mapsto [0, 1[$, and $g(x) = \frac{x}{m}$,
- $g : \mathbb{N} \mapsto ]0, 1]$, and $g(x) = \frac{x}{m-1}$,
- $g : \mathbb{N} \mapsto ]0, 1[$, and $g(x) = \frac{x+1/2}{m}$.

Linear congruential generators are implemented in the R function `congruRand`.

### 2.1.2 Multiple recursive generators

A generalisation of linear congruential generators are multiple recursive generators. They are based on the following recurrences

$$x_n = (a_1 x_{n-1} + \dots + a_k x_{n-k} c) \mod m,$$

where $k$ is a fixed integer. Hence the $n$th term of the sequence depends on the $k$ previous one. A particular case of this type of generators is when

$$x_n = (x_{n-37} + x_{n-100}) \mod 2^{30},$$

which is a Fibonacci-lagged generator[2]. The period is around $2^{129}$. This generator has been invented by Knuth (2002) and is generally called "Knuth-TAOCP-2002" or simply "Knuth-TAOCP"[3].

An integer version of this generator is implemented in the R function `runif` (see `RNG`). We include in the package the latest double version, which corrects undesirable deficiency. As described on Knuth's webpage[4] , the previous version of Knuth-TAOCP fails randomness test if we generate few sequences with several seeds. The cures to this problem is to discard the first 2000 numbers.

[2]see L'Ecuyer (1990).

[3]TAOCP stands for The Art Of Computer Programming, Knuth's famous book.

[4]go to `http://www-cs-faculty.stanford.edu/~knuth/news02.html#rng`.

#### 2.1.3 Mersenne-Twister

These two types of generators are in the big family of matrix linear congruential generators (cf. L'Ecuyer (1990)). But until here, no algorithms exploit the binary structure of computers (i.e. use binary operations). In 1994, Matsumoto and Kurita invented the TT800 generator using binary operations. But Matsumoto & Nishimura (1998) greatly improved the use of binary operations and proposed a new random number generator called Mersenne-Twister.

Matsumoto & Nishimura (1998) work on the finite set $N_2 = \{0, 1\}$, so a variable $x$ is represented by a vectors of $\omega$ bits (e.g. 32 bits). They use the following linear recurrence for the $n+i$th term:

$$x_{i+n} = x_{i+m} \oplus (x_i^{upp} | x_{i+1}^{low}) A,$$

where $n > m$ are constant integers, $x_i^{upp}$ (respectively $x_i^{low}$) means the upper (lower) $\omega - r$ ($r$) bits of $x_i$ and $A$ a $\omega \times \omega$ matrix of $N_2$. $|$ is the operator of concatenation, so $x_i^{upp}|x_{i+1}^{low}$ appends the upper $\omega - r$ bits of $x_i$ with the lower $r$ bits of $x_{i+1}$. After a right multiplication with the matrix A[1], $\oplus$ adds the result with $x_{i+m}$ bit to bit modulo two (i.e. $\oplus$ denotes the exclusive-or called xor).

Once provided an initial seed $x_0, \dots, x_{n-1}$, Mersenne Twister produces random integers in $0, \dots, 2^\omega - 1$. All operations used in the recurrence are bitwise operations, thus it is a very fast computation compared to modulus operations used in previous algorithms.

To increase the equidistribution, Matsumoto & Nishimura (1998) added a tempering step:

$$\begin{aligned} y_i &\leftarrow x_{i+n} \oplus (x_{i+n} >> u), \\ y_i &\leftarrow y_i \oplus ((y_i << s) \oplus b), \\ y_i &\leftarrow y_i \oplus ((y_i << t) \oplus c), \\ y_i &\leftarrow y_i \oplus (y_i >> l), \end{aligned}$$

where $>> u$ (resp. $<< s$) denotes a rightshift (leftshift) of $u$ ($s$) bits. At last, we transform random integers to reals with one of output functions $g$ proposed above.

Details of the order of the successive operations used in the Mersenne-Twister (MT) algorithm can be found at the page 7 of Matsumoto & Nishimura (1998). However, the least, we need to learn and to retain, is all these (bitwise) operations can be easily done in many computer languages (e.g in C) ensuring a very fast algorithm.

The set of parameters used are

- $(\omega, n, m, r) = (32, 624, 397, 31)$,
- $a = 0 \times 9908B0DF, b = 0 \times 9D2C5680, c = 0 \times EFC60000$,
- $u = 11$, $l = 18$, $s = 7$ and $t = 15$.

These parameters ensure a good equidistribution and a period of $2^{n\omega - r} - 1 = 2^{19937} - 1$.

The great advantages of the MT algorithm are a far longer period than any previous generators (greater than the period of Park & Miller (1988) sequence of $2^{32} - 1$ or the period of Knuth (2002) around $2^{129}$), a far better equidistribution (since it passed the DieHard test) as well as an **very** good computation time (since it used binary operations and not the costly real operation modullus).

MT algorithm is already implemented in R (function `runif`). However the package `randtoolbox` provide functions to compute a new version of Mersenne-Twister (the SIMD-oriented Fast Mersenne Twister algorithm) as well as the WELL (Well Equidistributed Long-period Linear) generator.

[1]Matrix $A$ equals to $\begin{pmatrix} 0 & I_{\omega-1} \\ & a \end{pmatrix}$ whose right multiplication can be done with a bitwise rightshift operation and an addition with integer $a$. See the section 2 of Matsumoto & Nishimura (1998) for explanations.

#### 2.1.4 Well Equidistributed Long-period Linear generators

The MT recurrence can be rewritten as

$$x_i = Ax_{i-1},$$

where $x_k$ are vectors of $N_2$ and $A$ a transition matrix. The charateristic polynom of $A$ is

$$\chi_A(z) \stackrel{\triangle}{=} \det(A-zI) = z^k - \alpha_1 z^{k-1} - \cdots - \alpha_{k-1}z - \alpha_k,$$

with coefficients $\alpha_k$'s in $N_2$. Those coefficients are linked with output integers by

$$x_{i,j} = (\alpha_1 x_{i-1,j} + \cdots + \alpha_k x_{i-k,j}) \mod 2$$

for all component $j$.

From Panneton et al. (2006), we have the period length of the recurrence reaches the upper bound $2^k - 1$ if and only if the polynom $\chi_A$ is a primitive polynomial over $N_2$.

The more complex is the matrix $A$ the slower will be the associated generator. Thus, we compromise between speed and quality (of equidistribution). If we denote by $\psi_d$ the set of all $d$-dimensional vectors produced by the generator from all initial *states*[1].

If we divide each dimension into $2^l$[2] cells (i.e. the unit hypercube $[0, 1[^d$ is divided into $2^{ld}$ cells), the set $\psi_d$ is said to be $(d, l)$-equidistributed if and only if each cell contains exactly $2^{k-dl}$ of its points. The largest dimension for which the set $\psi_d$ is $(d, l)$-equidistributed is denoted by $d_l$.

The great advantage of using this definition is we are not forced to compute random points to know the uniformity of a generator. Indeed, thanks to the linear structure of the recurrence we can express the property of bits of the current state. From this we define a dimension gap for $l$ bits resolution as $\delta_l = \lfloor k/l \rfloor - d_l$.

[1] The cardinality of $\psi_d$ is $2^k$.

[2] with $l$ an integer such that $l \leq \lfloor k/d \rfloor$.

An usual measure of uniformity is the sum of dimension gaps

$$\Delta_1 = \sum_{l=1}^{\omega} \delta_l.$$

Panneton et al. (2006) tries to find generators with a dimension gap sum $\Delta_1$ around zero and a number $Z_1$ of non-zero coefficients in $\chi_A$ around $k/2$. Generators with these two characteristics are called Well Equidistributed Long-period Linear generators. As a benchmark, Mersenne Twister algorithm is characterized with $k = 19937$, $\Delta_1 = 6750$ and $Z_1 = 135$.

The WELL generator is characterized by the following $A$ matrix

$$\begin{pmatrix} T_{5,7,0} & 0 & \dots & & & \\ T_0 & 0 & \dots & & & \\ 0 & I & \ddots & & & \\ & \ddots & \ddots & & & \\ & & \ddots & I & 0 & \\ & & & 0 & L & 0 \end{pmatrix},$$

where $T_.$ are specific matrices, $I$ the identity matrix and $L$ has ones on its "top left" corner. The first two lines are not entirely sparse but "fill" with $T_.$ matrices. All $T_.$'s matrices are here to change the state in a very efficient way, while the subdiagonal (nearly full of ones) is used to shift the unmodified part of the current state to the next one. See Panneton et al. (2006) for details.

The MT generator can be obtained with special values of $T_.$'s matrices. Panneton et al. (2006) proposes a set of parameters, where they computed dimension gap number $\Delta_1$. The full table can be found in Panneton et al. (2006), we only sum up parameters for those implemented in this package in table 1.

Let us note that for the last two generators a tempering step is possible in order to have maximally equidistributed generator (i.e. $(d, l)$-equidistributed for all $d$ and $l$). These generators are implemented in this package thanks to the C code of L'Ecuyer and Panneton.

| name | $k$ | $N_1$ | $\Delta_1$ |
|---|---|---|---|
| WELL512a | 512 | 225 | 0 |
| WELL1024a | 1024 | 407 | 0 |
| WELL19937a | 19937 | 8585 | 4 |
| WELL44497a | 44497 | 16883 | 7 |

Table 1: Specific WELL generators

#### 2.1.5 SIMD-oriented Fast Mersenne Twister algorithms

A decade after the invention of MT, Matsumoto & Saito (2008) enhances their algorithm with the computer of today, which have Single Instruction Mutiple Data operations letting to work conceptually with 128 bits integers.

MT and its successor are part of the family of multiple-recursive matrix generators since they verify a multiple recursive equation with matrix constants. For MT, we have the following recurrence

$$x_{k+n} = \underbrace{x_k \begin{pmatrix} I_{\omega-r} & 0 \\ 0 & 0 \end{pmatrix} A \oplus x_{k+1} \begin{pmatrix} 0 & 0 \\ 0 & I_r \end{pmatrix} A \oplus x_{k+m}}_{h(x_k, x_{k+1}, \dots, x_m, \dots, x_{k+n-1})}.$$

for the $k+n$th term.

Thus the MT recursion is entirely characterized by

$$h(\omega_0, \dots, \omega_{n-1}) = (\omega_0|\omega_1)A \oplus \omega_m,$$

where $\omega_i$ denotes the $i$th word integer (i.e. horizontal vectors of $N_2$).

The general recurrence for the SFMT algorithm extends MT recursion to

$$h(\omega_0, \dots, \omega_{n-1}) = \omega_0 A \oplus \omega_m B \oplus \omega_{n-2} C \oplus \omega_{n-1} D,$$

where $A, B, C, D$ are sparse matrices over $N_2$, $\omega_i$ are 128-bit integers and the degree of recursion is $n = \lceil \frac{19937}{128} \rceil = 156$.

The matrices $A, B, C$ and $D$ for a word $w$ are defined as follows,

- $wA = \left( w \overset{128}{<<} 8 \right) \oplus w$,
- $wB = \left( w \overset{32}{>>} 11 \right) \otimes c$, where $c$ is a 128-bit constant and $\otimes$ the bitwise AND operator,
- $wC = w \overset{128}{>>} 8$,
- $wD = w \overset{32}{<<} 18$,

where $\overset{128}{<<}$ denotes a 128-bit operation while $\overset{32}{>>}$ a 32-bit operation, i.e. an operation on the four 32-bit parts of 128-bit word $w$.

Hence the *transition function* of SFMT is given by

$$\begin{aligned} f : (N_2^{\omega})^n &\mapsto (N_2^{\omega})^n \\ (\omega_0, \dots, \omega_{n-1}) &\mapsto (\omega_1, \dots, \omega_{n-1}, h(\omega_0, \dots, \omega_{n-1})), \end{aligned}$$

where $(N_2^{\omega})^n$ is the *state* space.

The selection of recursion and parameters was carried out to find a good dimension of equidistribution for a given a period. This step is done by studying the characteristic polynomial of $f$. SFMT allow periods of $2^p - 1$ with $p$ a (prime) Mersenne exponent[1]. Matsumoto & Saito (2008) proposes the following set of exponents 607, 1279, 2281, 4253, 11213, 19937, 44497, 86243, 132049 and 216091.

The advantage of SFMT over MT is the computation speed, SFMT is twice faster without SIMD operations and nearly fourt times faster with SIMD operations. SFMT has also a better equidistribution[2] and a better recovery time from zeros-excess states[3]. The function `SFMT` provides an interface to the `C` code of Matsumoto and Saito.

[1] a Mersenne exponent is a prime number $p$ such that $2^p - 1$ is prime. Prime numbers of the form $2^p - 1$ have the special designation Mersenne numbers.

[2] See linear algebra arguments of Matsumoto & Nishimura (1998).

[3] states with too many zeros.

## 2.2 Quasi random generation

Before explaining and detailing quasi random generation, we must (quickly) explain Monte-Carlo[1] methods, which have been introduced in the forties. In this section, we follow the approach of Niederreiter (1978).

Let us work on the $d$-dimensional unit cube $I^d = [0,1]^d$ and with a (multivariate) bounded (Lebesgues) integrable function $f$ on $I^d$. Then we define the Monte Carlo approximation of integral of $f$ over $I^d$ by

$$\int_{I^d} f(x)dx \approx \frac{1}{n}\sum_{i=1}^{n} f(X_i),$$

where $(X_i)_{1\leq i\leq n}$ are independent random points from $I^d$.

The strong law of large numbers ensures the almost surely convergence of the approximation. Furthermore, the expected integration error is bounded by $O(\frac{1}{\sqrt{n}})$, with the interesting fact it does not depend on dimension $d$. Thus Monte Carlo methods have a wide range of applications.

The main difference between (pseudo) Monte-Carlo methods and quasi Monte-Carlo methods is that we no longer use random points $(x_i)_{1\leq i\leq n}$ but deterministic points. Unlike statistical tests, numerical integration does not rely on true randomness. Let us note that quasi Monte-Carlo methods date from the fifties, and have also been used for interpolation problems and integral equations solving.

In the following, we consider a sequence "'Furthermore the convergence condition on the sequence $(u_i)_i$ is to be uniformly distributed in the unit cube $I^d$ with the following sense:

$$\forall J \subset I^d, \lim_{n\to+\infty} \frac{1}{n}\sum_{i=1}^{n} 1\!\!1_J(u_i) = \lambda_d(J),$$

where $\lambda_d$ stands for the $d$-dimensional volume (i.e. the $d$-dimensional Lebesgue measure) and $1\!\!1_J$ the indicator function of subset $J$. The problem is that our discrete sequence will never constitute a "fair" distribution in $I^d$, since there will always be a small subset with no points.

Therefore, we need to consider a more flexible definition of uniform distribution of a sequence. Before introducing the discrepancy, we need to define $\text{Card}_E(u_1,\dots,u_n)$ as $\sum_{i=1}^n 1\!\!1_E(u_i)$ the number of points in subset $E$. Then the discrepancy $D_n$ of the $n$ points $(u_i)_{1\leq i\leq n}$ in $I^d$ is given by

$$D_n = \sup_{J\in\mathcal{J}} \left| \frac{\text{Card}_J(u_1,\dots,u_n)}{n} - \lambda_d(J) \right|$$

where $\mathcal{J}$ denotes the family of all subintervals of $I^d$ of the form $\prod_{i=1}^d [a_i,b_i]$. If we took the family of all subintervals of $I^d$ of the form $\prod_{i=1}^d [0,b_i]$, $D_n$ is called the star discrepancy (cf. Niederreiter (1992)).

Let us note that the $D_n$ discrepancy is nothing else than the $L_\infty$-norm over the unit cube of the difference between the empirical ratio of points $(u_i)_{1\leq i\leq n}$ in a subset $J$ and the theoretical point number in $J$. A $L_2$-norm can be defined as well, see Niederreiter (1992) or Jäckel (2002).

The integral error is bounded by

$$\left| \frac{1}{n}\sum_{i=1}^{n} f(u_i) - \int_{I^d} f(x)dx \right| \leq V_d(f) D_n,$$

where $V_d(f)$ is the $d$-dimensional Hardy and Krause variation[2] of $f$ on $I^d$ (supposed to be finite).

Actually the integral error bound is the product of two independent quantities: the variability of function $f$ through $V_d(f)$ and the regularity of the sequence through $D_n$. So, we want to minimize the discrepancy $D_n$ since we generally do not have a choice in the "problem" function $f$.

[1] according to wikipedia the name comes from a famous casino in Monaco.

[2] Interested readers can find the definition page 966 of Niederreiter (1978). In a sentence, the Hardy and Krause variation of $f$ is the supremum of sums of $d$-dimensional delta operators applied to function $f$.

We will not explain it but this concept can be extented to subset $J$ of the unit cube $I^d$ in order to have a similar bound for $\int_J f(x)dx$.

In the literature, there were many ways to find sequences with small discrepancy, generally called low-discrepancy sequences or quasi-random points. A first approach tries to find bounds for these sequences and to search the good parameters to reach the lower bound or to decrease the upper bound. Another school tries to exploit regularity of function $f$ to decrease the discrepancy. Sequences coming from the first school are called quasi-random points while those of the second school are called good lattice points.

### 2.2.1 Quasi-random points and discrepancy

Until here, we do not give any example of quasi-random points. In the unidimensional case, an easy example of quasi-random points is the sequence of $n$ terms given by $(\frac{1}{2n}, \frac{3}{2n}, \dots, \frac{2n-1}{2n})$. This sequence has a discrepancy $\frac{1}{n}$, see Niederreiter (1978) for details.

The problem with this finite sequence is it depends on $n$. And if we want different points numbers, we need to recompute the whole sequence. In the following, we will on work the first $n$ points of an infinite sequence in order to use previous computation if we increase $n$.

Moreover we introduce the notion of discrepancy on a finite sequence $(u_i)_{1\leq i\leq n}$. In the above example, we are able to calculate exactly the discrepancy. With infinite sequence, this is no longer possible. Thus, we will only try to estimate asymptotic equivalents of discrepancy.

The discrepancy of the average sequence of points is governed by the law of the iterated logarithm :

$$\limsup_{n\to+\infty} \frac{\sqrt{n}D_n}{\sqrt{\log\log n}} = 1,$$

which leads to the following asymptotic equivalent for $D_n$:

$$D_n = O\left(\sqrt{\frac{\log\log n}{n}}\right).$$

### 2.2.2 Van der Corput sequences

An example of quasi-random points, which have a low discrepancy, is the (unidimensional) Van der Corput sequences.

Let $p$ be a prime number. Every integer $n$ can be decomposed in the $p$ basis, i.e. there exists some integer $k$ such that

$$n = \sum_{j=1}^{k} a_j p^j.$$

Then, we can define the radical-inverse function of integer $n$ as

$$\phi_p(n) = \sum_{j=1}^{k} \frac{a_j}{p^{j+1}}.$$

And finally, the Van der Corput sequence is given by $(\phi_p(0), \phi_p(1), \dots, \phi_p(n), \dots) \in [0, 1[$. First terms of those sequence for prime numbers 2 and 3 are given in table 2.

| | $n$ in $p$-basis | | | $\phi_p(n)$ | | |
|---|---|---|---|---|---|---|
| $n$ | $p=2$ | $p=3$ | $p=5$ | $p=2$ | $p=3$ | $p=5$ |
| 0 | 0 | 0 | 0 | 0 | 0 | 0 |
| 1 | 1 | 1 | 1 | 0.5 | 0.333 | 0.2 |
| 2 | 10 | 2 | 2 | 0.25 | 0.666 | 0.4 |
| 3 | 11 | 10 | 3 | 0.75 | 0.111 | 0.6 |
| 4 | 100 | 11 | 4 | 0.125 | 0.444 | 0.8 |
| 5 | 101 | 12 | 10 | 0.625 | 0.777 | 0.04 |
| 6 | 110 | 20 | 11 | 0.375 | 0.222 | 0.24 |
| 7 | 111 | 21 | 12 | 0.875 | 0.555 | 0.44 |
| 8 | 1000 | 22 | 13 | 0.0625 | 0.888 | 0.64 |

Table 2: Van der Corput first terms

The big advantage of Van der Corput sequence is that they use $p$-adic fractions easily computable on the binary structure of computers.

#### 2.2.3 Halton sequences

The $d$-dimensional version of the Van der Corput sequence is known as the Halton sequence. The $n$th term of the sequence is define as

$$(\phi_{p_1}(n), \dots, \phi_{p_d}(n)) \in I^d,$$

where $p_1, \dots, p_d$ are pairwise relatively prime bases. The discrepancy of the Halton sequence is asymptotically $O\left(\frac{\log(n)^d}{n}\right)$.

The following Halton theorem gives us better discrepancy estimate of finite sequences. For any dimension $d \geq 1$, there exists an finite sequence of points in $I^d$ such that the discrepancy

$$D_n = O\left(\frac{\log(n)^{d-1}}{n}\right)^1.$$

Therefore, we have a significant guarantee there exists quasi-random points which are outperforming than traditional Monte-Carlo methods.

#### 2.2.4 Faure sequences

The Faure sequences is also based on the decomposition of integers into prime-basis but they have two differences: it uses only one prime number for basis and it permutes vector elements from one dimension to another.

The basis prime number is chosen as the smallest prime number greater than the dimension $d$, i.e. 3 when $d = 2$, 5 when $d = 3$ or 4 etc... In the Van der Corput sequence, we decompose integer $n$ into the $p$-basis:

$$n = \sum_{j=1}^{k} a_j p^j.$$

Let $a_{1,j}$ be integer $a_j$ used for the decomposition of $n$. Now we define a recursive permutation of $a_j$:

$$\forall 2 \leq D \leq d, a_{D,j} = \sum_{j=i}^{k} C_j^i a_{D-1,j} \mod p,$$

where $C_j^i$ denotes standard combination $\frac{j!}{i!(j-i)!}$. Then we take the radical-inversion $\phi_p(a_{D,1}, \dots, a_{D,k})$ defined as

$$\phi_p(a_1, \dots, a_k) = \sum_{j=1}^{k} \frac{a_j}{p^{j+1}},$$

which is the same as above for $n$ defined by the $a_{D,i}$'s.

Finally the ($d$-dimensional) Faure sequence is defined by

$$(\phi_p(a_{1,1}, \dots, a_{1,k}), \dots, \phi_p(a_{d,1}, \dots, a_{d,k})) \in I^d.$$

In the bidimensional case, we work in 3-basis, first terms of the sequence are listed in table 3.

| $n$ | $a_{13}a_{12}a_{11}$[2] | $a_{23}a_{22}a_{21}$ | $\phi(a_{13}..)$ | $\phi(a_{23}..)$ |
|---|---|---|---|---|
| 0 | 000 | 000 | 0 | 0 |
| 1 | 001 | 001 | 1/3 | 1/3 |
| 2 | 002 | 002 | 2/3 | 2/3 |
| 3 | 010 | 012 | 1/9 | 7/9 |
| 4 | 011 | 010 | 4/9 | 1/9 |
| 5 | 012 | 011 | 7/9 | 4/9 |
| 6 | 020 | 021 | 2/9 | 5/9 |
| 7 | 021 | 022 | 5/9 | 8/9 |
| 8 | 022 | 020 | 8/9 | 2/9 |
| 9 | 100 | 100 | 1/27 | 1/27 |
| 10 | 101 | 101 | 10/27 | 10/27 |
| 11 | 102 | 102 | 19/27 | 19/27 |
| 12 | 110 | 112 | 4/27 | 22/27 |
| 13 | 111 | 110 | 12/27 | 4/27 |
| 14 | 112 | 111 | 22/27 | 12/27 |

Table 3: Faure first terms

#### 2.2.5 Sobol sequences

This sub-section is taken from unpublished work of Diethelm Wuertz.

The Sobol sequence $x_n = (x_{n,1}, \dots, x_{n,d})$ is generated from a set of binary functions of length $\omega$ bits ($v_{i,j}$ with $i = 1, \dots, \omega$ and $j =$

[1] if the sequence has at least two points, cf. Niederreiter (1978).

[2] we omit commas for simplicity.

$1, \ldots, d)$. $v_{i,j}$, generally called direction numbers are numbers related to primitive (irreducible) polynomials over the field $\{0, 1\}$.

In order to generate the $j$th dimension, we suppose that the primitive polynomial in dimension $j$ is

$$p_j(x) = x^q + a_1 x^{q-1} + \cdots + a_{q-1}x + 1.$$

Then we define the following $q$-term recurrence relation on integers $(M_{i,j})_i$

$$M_{i,j} = 2a_1 M_{i-1,j} \oplus 2^2 a_2 M_{i-2,j} \oplus \ldots \oplus 2^{q-1} a_{q-1} M_{i-q+1,j} \oplus 2^q a_q M_{i-q,j} \oplus M_{i-q}$$

where $i > q$.

This allow to compute direction numbers as

$$v_{i,j} = M_{i,j}/2^i.$$

This recurrence is initialized by the set of arbitrary odd integers $v_{1,j}2^{\omega}, \ldots, v_{,j}2^q\omega$, which are smaller than $2, \ldots, 2^q$ respectively. Finally the $j$th dimension of the $n$th term of the Sobol sequence is with

$$x_{n,j} = b_1 v_{1,j} \oplus b_2 v_{2,j} \oplus \cdots \oplus v_{\omega,j},$$

where $b_k$'s are the bits of integer $n = \sum_{k=0}^{\omega-1} b_k 2^k$. The requirement is to use a different primitive polynomial in each dimension. An e?cient variant to implement the generation of Sobol sequences was proposed by Antonov & Saleev (1979). The use of this approach is demonstrated in Bratley & Fox (1988) and Press et al. (1996).

#### 2.2.6 Scrambled Sobol sequences

Randomized QMC methods are the basis for error estimation. A generic recipe is the following: Let $A_1, \ldots, A_n$ be a QMC point set and $X_i$ a scrambled version of $A_i$. Then we are searching for randomizations which have the following properties:

- Uniformity: The $X_i$ makes the approximator $\hat{I} = \frac{1}{N}\sum_{i=1}^{N} f(X_i)$ an unbiased estimate of $I = \int_{[0,1]^d} f(x)dx$.
- Equidistribution: The $X_i$ form a point set with probability 1; i.e. the random- ization process has preserved whatever special properties the underlying point set had.

The Sobol sequences can be scrambled by the Owen's type of scrambling, by the Faure-Tezuka type of scrambling, and by a combination of both.

The program we have interfaced to R is based on the ACM Algorithm 659 described by Bratley & Fox (1988) and Bratley et al. (1992). Modifications by Hong & Hickernell (2001) allow for a randomization of the sequences. Furthermore, in the case of the Sobol sequence we followed the implementation of Joe & Kuo (1999) which can handle up to 1111 dimensions.

To interface the Fortran routines to the R environment some modifications had to be performed. One important point was to make possible to re-initialize a sequence and to recall a sequence without renitialization from R. This required to remove BLOCKDATA, COMMON and SAVE statements from the original code and to pass the initialization variables through the argument lists of the subroutines, so that these variables can be accessed from R.

#### 2.2.7 Kronecker sequences

Another kind of low-discrepancy sequence uses irrational number and fractional part. The fractional part of a real $x$ is denoted by $\{x\} = x - \lfloor x \rfloor$. The infinite sequence $(n\{\alpha\})_{n\leq 0}$ has a bound for its discrepancy

$$D_n \leq C \frac{1 + \log n}{n}.$$

This family of infinite sequence $(n\{\alpha\})_{n\leq 0}$ is called the Kronecker sequence.

A special case of the Kronecker sequence is the Torus algorithm where irrational number $\alpha$ is a square root of a prime number. The $n$th term of the $d$-dimensional Torus algorithm is defined by

$$(n\{\sqrt{p_1}\}, \ldots, n\{\sqrt{p_d}\}) \in I^d,$$

where $(p_1, \dots, p_d)$ are prime numbers, generally the first $d$ prime numbers. With the previous inequality, we can derive an estimate of the Torus algorithm discrepancy:

$$O\left(\frac{1+\log n}{n}\right).$$

#### 2.2.8 Mixed pseudo quasi random sequences

Sometimes we want to use quasi-random sequences as pseudo random ones, i.e. we want to keep the good equidistribution of quasi-random points but without the term-to-term dependence.

One way to solve this problem is to use pseudo random generation to mix outputs of a quasi-random sequence. For example in the case of the Torus sequence, we have repeat for $1 \leq i \leq n$

- draw an integer $n_i$ from Mersenne-Twister in $\{0, \dots, 2^\omega - 1\}$
- then $u_i = \{n_i \sqrt{p}\}$

#### 2.2.9 Good lattice points

In the above methods we do not take into account a better regularity of the integrand function $f$ than to be of bounded variation in the sense of Hardy and Krause. Good lattice point sequences try to use the eventual better regularity of $f$.

If $f$ is 1-periodic for each variable, then the approximation with good lattice points is

$$\int_{I^d} f(x)dx \approx \frac{1}{n}\sum_{i=1}^{n} f\left(\frac{i}{n}g\right),$$

where $g \in \mathbb{Z}^d$ is suitable $d$-dimensional lattice point. To impose $f$ to be 1-periodic may seem too brutal. But there exists a method to transform $f$ into a 1-periodic function while preserving regularity and value of the integrand (see Niederreiter 1978, page 983).

We have the following theorem for good lattice points. For every dimension $d \geq 2$ and integer $n \geq 2$, there exists a lattice points $g \in \mathbb{Z}^d$ which coordinates relatively prime to $n$ such that the discrepancy $D_n$ of points $\{\frac{1}{n}g\}, \dots, \{\frac{n}{n}g\}$ satisfies

$$D_s < \frac{d}{n} + \frac{1}{2n}\left(\frac{7}{5} + 2\log m\right)^d.$$

Numerous studies of good lattice points try to find point $g$ which minimizes the discrepancy. Korobov test $g$ of the following form $\left(1, m, \dots, m^{d-1}\right)$ with $m \in \mathbb{N}$. Bahvalov tries Fibonnaci numbers $(F_1, \dots, F_d)$. Other studies look directly for the point $\alpha = \frac{g}{n}$ e.g. $\alpha = \left(p^{\frac{1}{d+1}}, \dots, p^{\frac{d}{d+1}}\right)$ or some cosinus functions. We let interested readers to look for detailed information in Niederreiter (1978).

## 3 Examples of distinguishing from truly random numbers

For a good generator, it is not computationally easy to distinguish the output of the generator from truly random numbers, if the seed or the index in the sequence is not known. In this section, we present examples of generators, whose output may be easily distinguished from truly random numbers.

An example of such a generator is the older version of Wichmann-Hill from 1982. For this generator, we can even predict the next number in the sequence, if we know the last already generated one. Verifying such a predicition is easy and it is, of course, not valid for truly random numbers. Hence, we can easily distinguish the output of the generator from truly random numbers. An implementation of this test in R derived from McCullough (2008) is as follows.

```
> wh.predict <- function(x)
+ {
+       M1 <- 30269
```

```
+      M2 <- 30307
+      M3 <- 30323
+      y <- round(M1*M2*M3*x)
+      s1 <- y %% M1
+      s2 <- y %% M2
+      s3 <- y %% M3
+      s1 <- (171*26478*s1) %% M1
+      s2 <- (172*26070*s2) %% M2
+      s3 <- (170*8037*s3) %% M3
+      (s1/M1 + s2/M2 + s3/M3) %% 1
+ }
> RNGkind("Wichmann-Hill")
> xnew <- runif(1)
> maxerr <- 0
> for (i in 1:1000) {
+      xold <- xnew
+      xnew <- runif(1)
+           err <- abs(wh.predict(xold) - xnew)
+      maxerr <- max(err, maxerr)
+ }
> print(maxerr)
```

```
[1] 0
```

The printed error is 0 on some machines and less than $5 \cdot 10^{-16}$ on other machines. This is clearly different from the error obtained for truly random numbers, which is close to 1.

The requirement that the output of a random number generator should not be distinguishable from the truly random numbers by a simple computation, is directly related to the way, how a generator is used. Typically, we use the generated numbers as an input to a computation and we expect that the distribution of the output (for different seeds or for different starting indices in the sequence) is the same as if the input are truly random numbers. A failure of this assumption implies, besides of a wrong result of our simulation, that observing the output of the computation allows to distinguish the output from the generator from truly random numbers. Hence, we want to use a generator, for which we may expect that the calculations used in the intended application cannot distinguish its output from truly random numbers.

In this context, it has to be noted that many of the currently used generators for simulations can be distinguished from truly random numbers using the arithmetic mod 2 (XOR operation) applied to individual bits of the output numbers. This is true for Mersenne Twister, SFMT and also all WELL generators. The basis for tolerating this is based on two facts. First, the arithmetic mod 2 and extracting individual bits of real numbers is not directly used in typical simulation problems and real valued functions, which represent these operations, are extremely discontinuous and such functions also do not typically occur in simulation problems. Another reason is that we need to observe quite a long history of the output to detect the difference from true randomness. For example, for Mersenne Twister, we need 624 consecutive numbers.

On the other hand, if we use a cryptographically strong pseudorandom number generator, we may avoid distinguishing from truly random numbers under any known efficient procedure. Such generators are typically slower than Mersenne Twister type generators. The factor of slow down is, for example for AES, about 5. However, note that for simulation problems, which require intensive computation besides the generating random numbers, using slower, but better, generator implies only negligible slow down of the computation as a whole.

# 4 Description of the random generation functions

In this section, we detail the R functions implemented in `randtoolbox` and give examples.

## 4.1 Pseudo random generation

For pseudo random generation, R provides many algorithms through the function `runif` parametrized with `.Random.seed`. We encourage readers to look in the corresponding help

pages for examples and usage of those functions. Let us just say `runif` use the Mersenne-Twister algorithm by default and other generators such as Wichmann-Hill, Marsaglia-Multicarry or Knuth-TAOCP-2002[1].

### 4.1.1 congruRand

The `randtoolbox` package provides two pseudo-random generators functions : `congruRand` and `SFMT`. `congruRand` computes linear congruential generators, see sub-section 2.1.1. By default, it computes the Park & Miller (1988) sequence, so it needs only the observation number argument. If we want to generate 10 random numbers, we type

```
> congruRand(10)
```

```
 [1] 0.8053849 0.1034877 0.3170663
 [4] 0.9328638 0.6410619 0.3268605
 [7] 0.5446692 0.2560190 0.9116396
[10] 0.9272115
```

One will quickly note that two calls to `congruRand` will not produce the same output. This is due to the fact that we use the machine time to initiate the sequence at each call. But the user can set the *seed* with the function `setSeed`:

```
> setSeed(1)
> congruRand(10)
```

```
 [1] 7.826369e-06 1.315378e-01
 [3] 7.556053e-01 4.586501e-01
 [5] 5.327672e-01 2.189592e-01
 [7] 4.704462e-02 6.788647e-01
 [9] 6.792964e-01 9.346929e-01
```

One can follow the evolution of the $n$th integer generated with the option `echo=TRUE`.

[1] see Wichmann & Hill (1982), Marsaglia (1994) and Knuth (2002) for details.

```
> setSeed(1)
> congruRand(10, echo=TRUE)
```

```
1 th integer generated : 1
2 th integer generated : 16807
3 th integer generated : 282475249
4 th integer generated : 1622650073
5 th integer generated : 984943658
6 th integer generated : 1144108930
7 th integer generated : 470211272
8 th integer generated : 101027544
9 th integer generated : 1457850878
10 th integer generated : 1458777923
 [1] 7.826369e-06 1.315378e-01
 [3] 7.556053e-01 4.586501e-01
 [5] 5.327672e-01 2.189592e-01
 [7] 4.704462e-02 6.788647e-01
 [9] 6.792964e-01 9.346929e-01
```

We can check that those integers are the 10 first terms are listed in table 4, coming from `http://www.firstpr.com.au/dsp/rand31/`.

| $n$ | $x_n$ | $n$ | $x_n$ |
|---|---|---|---|
| 1 | 16807 | 6 | 470211272 |
| 2 | 282475249 | 7 | 101027544 |
| 3 | 1622650073 | 8 | 1457850878 |
| 4 | 984943658 | 9 | 1458777923 |
| 5 | 1144108930 | 10 | 2007237709 |

Table 4: 10 first integers of Park & Miller (1988) sequence

We can also check around the 10000th term. From the site `http://www.firstpr.com.au/dsp/rand31/`, we know that 9998th to 10002th terms of the Park-Miller sequence are 925166085, 1484786315, 1043618065, 1589873406, 2010798668. The `congruRand` generates

```
> setSeed(1614852353)
> congruRand(5, echo=TRUE)
```

```
1 th integer generated : 1614852353
2 th integer generated : 925166085
```

```
3 th integer generated : 1484786315
4 th integer generated : 1043618065
5 th integer generated : 1589873406
[1] 0.4308140 0.6914075 0.4859725
[4] 0.7403425 0.9363511
```

with 1614852353 being the 9997th term of Park-Miller sequence.

However, we are not limited to the Park-Miller sequence. If we change the modulus, the increment and the multiplier, we get other random sequences. For example,

```
> setSeed(12)
> congruRand(5, mod = 2^8, mult = 25, incr = 16, echo = TRUE)
```

```
1 th integer generated : 12
2 th integer generated : 60
3 th integer generated : 236
4 th integer generated : 28
5 th integer generated : 204
[1] 0.234375 0.921875 0.109375
[4] 0.796875 0.984375
```

Those values are correct according to Planchet et al. 2005, page 119.

Here is a example list of RNGs computable with `congruRand`:

| RNG | mod | mult | incr |
|---|---|---|---|
| Knuth - Lewis | $2^{32}$ | 1664525 | $1.01e9$[1] |
| Lavaux - Jenssens | $2^{48}$ | 31167285 | 1 |
| Haynes | $2^{64}$ | $6.36e17$[2] | 1 |
| Marsaglia | $2^{32}$ | 69069 | 0 |
| Park - Miller | $2^{31}-1$ | 16807 | 0 |

Table 5: some linear RNGs

One may wonder why we implement such a short-period algorithm since we know the Mersenne-Twister algorithm. It is provided to make comparisons with other algorithms. The Park-Miller RNG should **not** be viewed as a "good" random generator.

Finally, `congruRand` function has a `dim` argument to generate `dim`- dimensional vectors of random numbers. The $n$th vector is build with $d$ consecutive numbers of the RNG sequence (i.e. the $n$th term is the $(u_{n+1}, \dots, u_{n+d})$).

#### 4.1.2 SFMT

The SF- Mersenne Twister algorithm is described in sub-section 2.1.5. Usage of `SFMT` function implementing the SF-Mersenne Twister algorithm is the same. First argument `n` is the number of random variates, second argument `dim` the dimension.

```
> SFMT(10)
> SFMT(5, 2) #bi dimensional variates
```

```
 [1] 0.8459674 0.5347039 0.6465131
 [4] 0.1762214 0.3349911 0.3638026
 [7] 0.1907544 0.9033059 0.7597441
[10] 0.5604641
```

```
          [,1]      [,2]
[1,] 0.4192898 0.8838815
[2,] 0.4100001 0.3256978
[3,] 0.5865271 0.3881290
[4,] 0.6438645 0.1282196
[5,] 0.8871303 0.1700034
```

A third argument is `mexp` for Mersenne exponent with possible values (607, 1279, 2281, 4253, 11213, 19937, 44497, 86243, 132049 and 216091). Below an example with a period of $2^{607}-1$:

```
> SFMT(10, mexp = 607)
```

```
 [1] 0.284496770 0.014260865 0.453397471
 [4] 0.008893187 0.761834924 0.841244282
```

[1] 1013904223.
[2] 636412233846793005.

```
 [7] 0.571004426 0.031522958 0.445546979
[10] 0.056950098
```

Furthermore, following the advice of Matsumoto & Saito (2008) for each exponent below 19937, `SFMT` uses a different set of parameters[1] in order to increase the independence of random generated variates between two calls. Otherwise (for greater exponent than 19937) we use one set of parameters[2].

We must precise that we do **not** implement the SFMT algorithm, we "just" use the C code of Matsumoto & Saito (2008). For the moment, we do not fully use the strength of their code. For example, we do not use block generation and SSE2 SIMD operations.

## 4.2 Quasi-random generation

### 4.2.1 Halton sequences

The function `halton` implements both the Van Der Corput (unidimensional) and Halton sequences. The usage is similar to pseudo-RNG functions

```
> halton(10)
> halton(10, 2)
```

```
 [1] 0.5000 0.2500 0.7500 0.1250 0.6250
 [6] 0.3750 0.8750 0.0625 0.5625 0.3125
```

```
        [,1]       [,2]
 [1,] 0.5000 0.33333333
 [2,] 0.2500 0.66666667
 [3,] 0.7500 0.11111111
 [4,] 0.1250 0.44444444
 [5,] 0.6250 0.77777778
 [6,] 0.3750 0.22222222
 [7,] 0.8750 0.55555556
 [8,] 0.0625 0.88888889
 [9,] 0.5625 0.03703704
[10,] 0.3125 0.37037037
```

You can use the `init` argument set to FALSE (default is TRUE) if you want that two calls to `halton` functions do not produce the same sequence (but the second call continues the sequence from the first call.

```
> halton(5)
> halton(5, init=FALSE)
```

```
[1] 0.500 0.250 0.750 0.125 0.625
```

```
[1] 0.3750 0.8750 0.0625 0.5625 0.3125
```

`init` argument is also available for other quasi-RNG functions.

### 4.2.2 Sobol sequences

The function `sobol` implements the Sobol sequences with optional sampling (Owen, Faure-Tezuka or both type of sampling). This subsection also comes from an unpublished work of Diethelm Wuertz.

To use the different scrambling option, you just to use the `scrambling` argument: 0 for (the default) no scrambling, 1 for Owen, 2 for Faure-Tezuka and 3 for both types of scrambling.

```
> sobol(10)
> sobol(10, scramb=3)
```

```
 [1] 0.5000 0.7500 0.2500 0.3750 0.8750
 [6] 0.6250 0.1250 0.1875 0.6875 0.9375
```

[1] this can be avoided with `usepset` argument to `FALSE`.

[2] These parameter sets can be found in the C function `initSFMT` in `SFMT.c` source file.

```
 [1] 0.08301502 0.40333283 0.79155719
 [4] 0.90135312 0.29438373 0.22406116
 [7] 0.58105069 0.62985182 0.05026767
[10] 0.49559012
```

It is easier to see the impact of scrambling by plotting two-dimensional sequence in the unit square. Below we plot the default Sobol sequence and Sobol scrambled by Owen algorithm, see figure 1.

```
> par(mfrow = c(2,1))
> plot(sobol(1000, 2))
> plot(sobol(10^3, 2, scram=1))
```

#### 4.2.3 Faure sequences

In a near future, `randtoolbox` package will have an implementation of Faure sequences. For the moment, there is no function `faure`.

#### 4.2.4 Torus algorithm (or Kronecker sequence)

The function `torus` implements the Torus algorithm.

```
> torus(10)
```

```
 [1] 0.41421356 0.82842712 0.24264069
 [4] 0.65685425 0.07106781 0.48528137
 [7] 0.89949494 0.31370850 0.72792206
[10] 0.14213562
```

These numbers are fractional parts of $\sqrt{2}, 2\sqrt{2}, 3\sqrt{2}, \ldots$, see sub-section 2.2.1 for details.

```
> torus(5, use =TRUE)
```

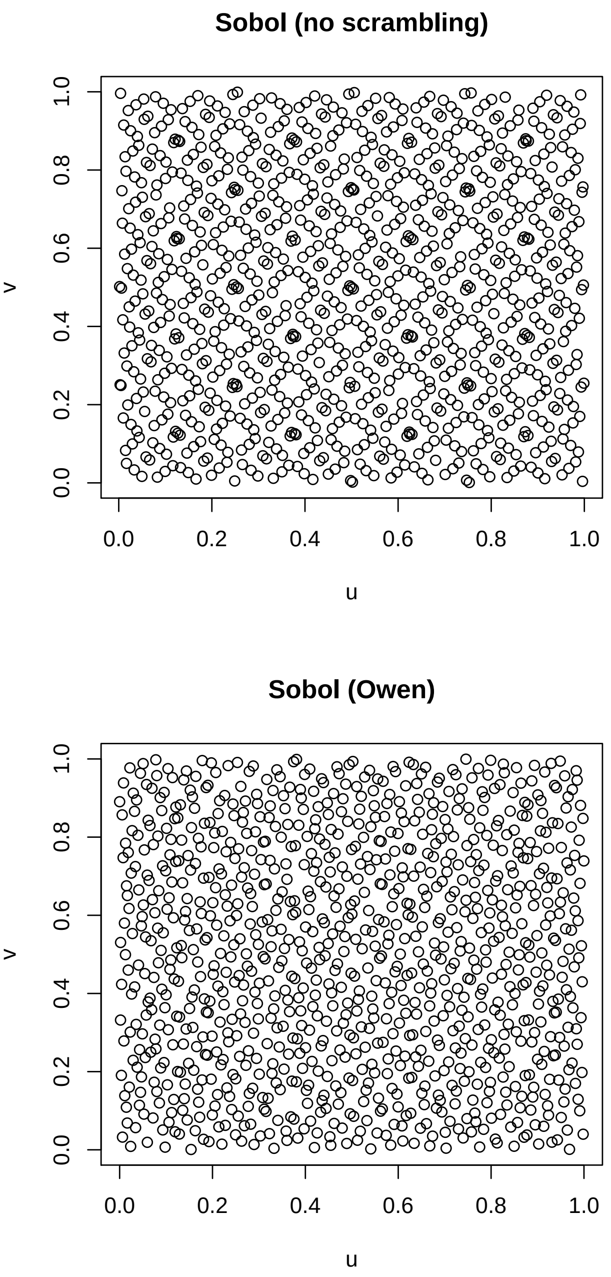


Figure 1: Sobol (two sampling types)

```
[1] 0.67480112 0.08901469 0.50322825
[4] 0.91744181 0.33165537
```

The optional argument `useTime` can be used to the machine time or not to initiate the seed. If we do not use the machine time, two calls of `torus` produces obviously the **same** output.

If we want the random sequence with prime number 7, we just type:

```
> torus(5, p =7)
```

```
[1] 0.6457513 0.2915026 0.9372539
[4] 0.5830052 0.2287566
```

The `dim` argument is exactly the same as `congruRand` or `SFMT`. By default, we use the first prime numbers, e.g. 2, 3 and 5 for a call like `torus(10, 3)`. But the user can specify a set of prime numbers, e.g. `torus(10, 3, c(7, 11, 13))`. The dimension argument is limited to 100 000[1].

As described in sub-section 2.2.8, one way to deal with serial dependence is to mix the Torus algorithm with a pseudo random generator. The `torus` function offers this operation thanks to argument `mixed` (the Torus algorithm is mixed with SFMT).

```
> torus(5,  mixed =TRUE)
```

```
[1] 0.93812275 0.48763180 0.09480286
[4] 0.71201205 0.67966747
```

In order to see the difference between, we can plot the empirical autocorrelation function (`acf` in R), see figure 2.

```
> par(mfrow = c(2,1))
> acf(torus(10^5))
> acf(torus(10^5, mix=TRUE))
```

## 4.3 Visual comparisons

To understand the difference between pseudo and quasi RNGs, we can make visual comparisons of how random numbers fill the unit square.

[1]the first 100 000 prime numbers are take from `http://primes.utm.edu/lists/small/millions/`.

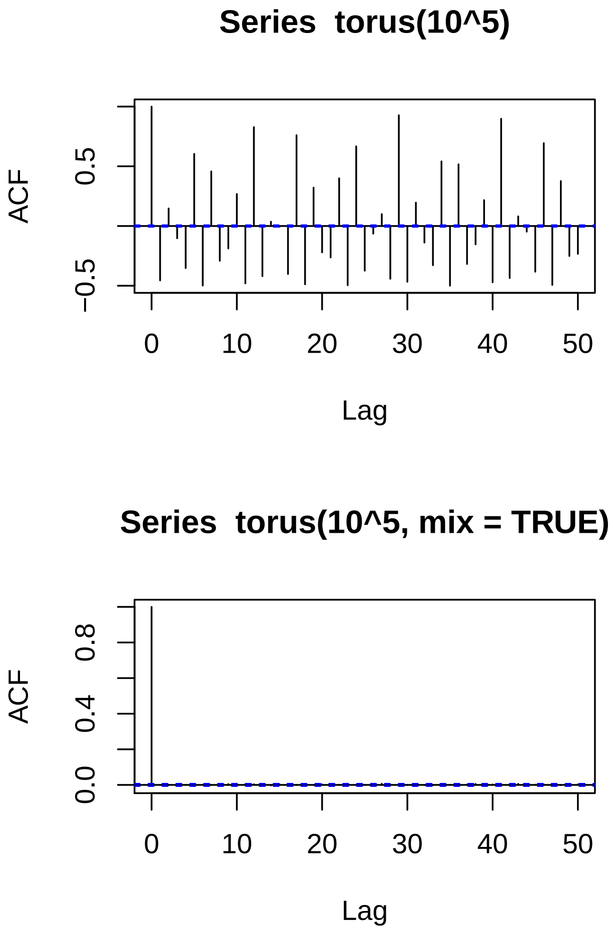


Figure 2: Auto-correlograms

First we compare SFMT algorithm with Torus algorithm on figure 3.

```
> par(mfrow = c(2,1))
> plot(SFMT(1000, 2))
> plot(torus(10^3, 2))
```

Secondly we compare WELL generator with Faure-Tezuka-scrambled Sobol sequences on figure 4.

```
> par(mfrow = c(2,1))
> plot(WELL(1000, 2))
> plot(sobol(10^3, 2, scram=2))
```

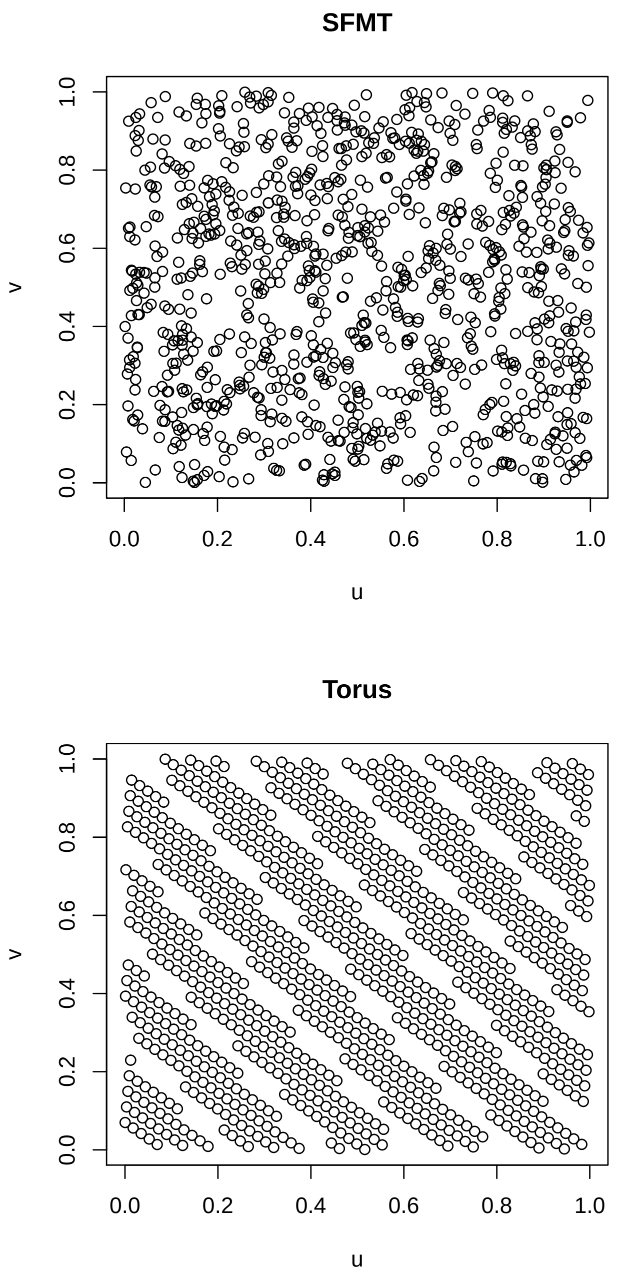


Figure 3: SFMT vs. Torus algorithm

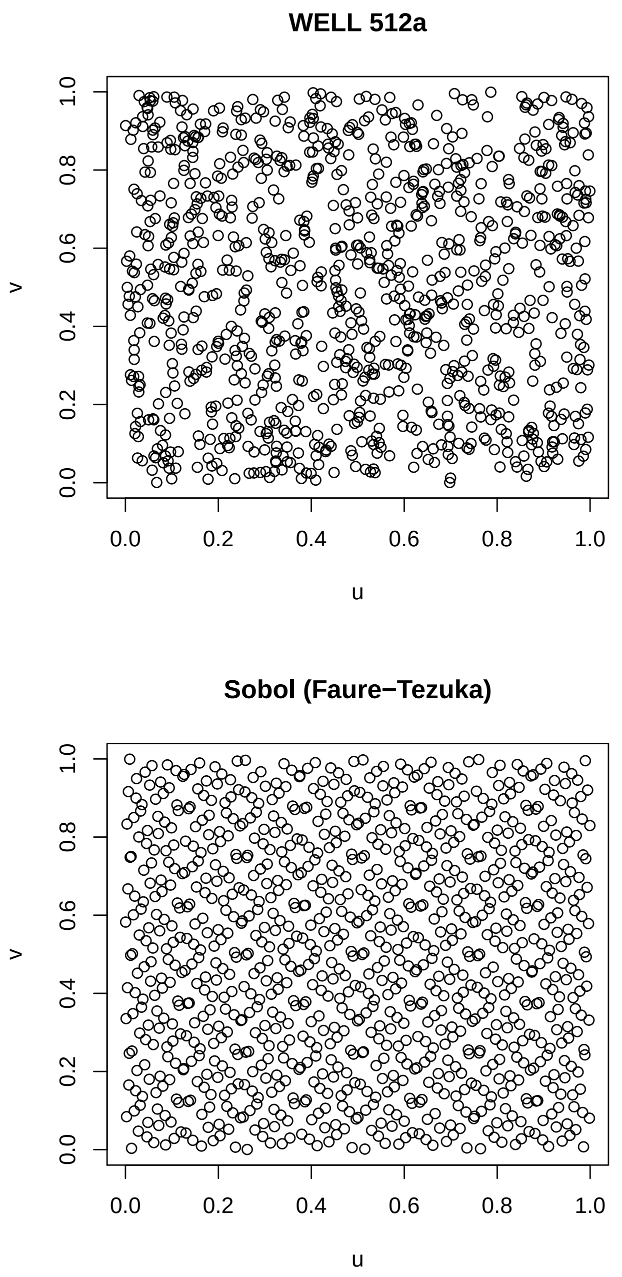


Figure 4: WELL vs. Sobol

## 4.4 Applications of QMC methods

### 4.4.1 $d$ dimensional integration

Now we will show how to use low-discrepancy sequences to compute a $d$-dimensional integral defined on the unit hypercube. We want compute

$$
\begin{aligned}
I_{cos}(d) &= \int_{\mathbb{R}^d} cos(||x||) e^{||x||^2} dx \\
&\approx \frac{\pi^{d/2}}{n} \sum_{i=1}^{n} cos\left( \sqrt{\sum_{j=1}^{d} \left(\Phi^{-1}\right)^2 (t_{ij})} \right)
\end{aligned}
$$

where $\Phi^{-1}$ denotes the quantile function of the standard normal distribution.

We simply use the following code to compute the $I_{cos}(25)$ integral whose exact value is $-1356914$.

```
> I25 <- -1356914
> nb <- c(1200, 14500, 214000)
> ans <- NULL
> for(i in 1:3)
+ {
+         tij <- sobol(nb[i], dim=25, scramb=2, norm=TRUE )
+         Icos <- mean(cos(sqrt( apply( tij^2/2, 1, sum ) ))) * pi^(25/2)
+         ans <- rbind(ans, c(n=nb[i], I25=Icos, Delta=(Icos-I25)/I25 ))
+ }
> data.frame(ans)
```

```
       n      I25         Delta
1   1200 -1355379 -1.131576e-03
2  14500 -1357216  2.222451e-04
3 214000 -1356909 -3.810502e-06
```

The results obtained from the Sobol Monte Carlo method in comparison to those obtained by Papageorgiou & Traub (2000) with a generalized Faure sequence and in comparison with the quadrature rules of Namee & Stenger (1967), Genz (1982) and Patterson (1968) are listed in the following table.

| n | 1200 | 14500 | 214000 |
|---|---|---|---|
| Faure (P&T) | 0.001 | 0.0005 | 0.00005 |
| Sobol (s=0) | 0.02 | 0.003 | 0.00006 |
| s=1 | 0.004 | 0.0002 | 0.00005 |
| s=2 | 0.001 | 0.0002 | 0.000002 |
| s=3 | 0.002 | 0.0009 | 0.00003 |
| Quadrature (McN&S) | 2 | 0.75 | 0.07 |
| G&P | 2 | 0.4 | 0.06 |

Table 6: list of errors

#### 4.4.2 Pricing of a Vanilla Call

In this sub-section, we will present one financial application of QMC methods. We want to price a vanilla European call in the framework of a geometric Brownian motion for the underlying asset. Those options are already implemented in the package `fOptions` of `Rmetrics` bundle[1].

The payoff of this classical option is

$$f(S_T) = (S_T - K)_+,$$

where $K$ is the strike price. A closed formula for this call was derived by Black & Scholes (1973).

The Monte Carlo method to price this option is quite simple

1. simulate $s_{T,i}$ for $i = 1 \dots n$ from starting point $s_0$,
2. compute the mean of the discounted payoff $\frac{1}{n}\sum_{i=1}^{n} e^{-rT}(s_{T,i} - K)_+$.

With parameters ($S_0 = 100$, $T = 1$, $r = 5\%$, $K = 60$, $\sigma = 20\%$), we plot the relative error as a function of number of simulation $n$ on figure 5.

We test two pseudo-random generators (namely Park Miller and SF-Mersenne Twister) and one quasi-random generator (Torus algorithm). No code will be shown, see the file `qmc.R` in the package source. But we use a step-by-step simulation for the Brownian motion simulation and the inversion function method for Gaussian distribution simulation (default in R).

As showed on figure 5, the convergence of Monte Carlo price for the Torus algorithm is extremely fast. Whereas for SF-Mersenne Twister and Park Miller prices, the convergence is very slow.

#### 4.4.3 Pricing of a DOC

Now, we want to price a barrier option: a down-out call i.e. an Downward knock-Out

[1] created by Wuertz et al. (2007*b*).

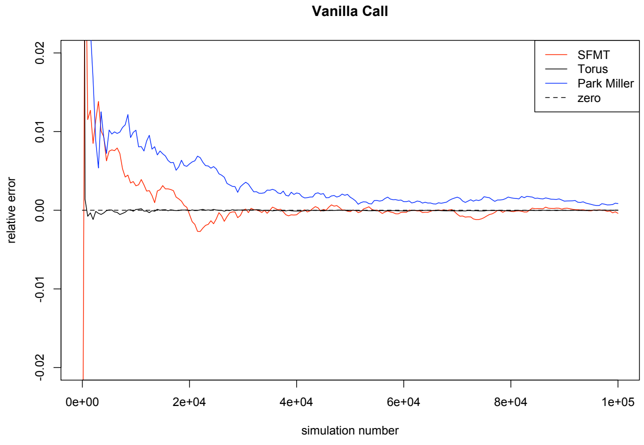


Figure 5: Error function for Vanilla call

Call[1]. These kind of options belongs to the path-dependent option family, i.e. we need to simulate whole trajectories of the underlying asset $S$ on $[0, T]$.

In the same framework of a geometric Brownian motion, there exists a closed formula for DOCs (see Rubinstein & Reiner (1991)). Those options are already implemented in the package `fExoticOptions` of `Rmetrics` bundle[2].

The payoff of a DOC option is

$$f(S_T) = (S_T - K)_+ \mathbb{1}_{(\tau_H > T)},$$

where $K$ is the strike price, $T$ the maturity, $\tau_H$ the stopping time associated with the barrier $H$ and $S_t$ the underlying asset at time $t$.

As the price is needed on the whole period $[0, T]$, we produc as follows:

1. start from point $s_{t_0}$,
2. for simulation $i = 1 \dots n$ and time index $j = 1 \dots d$
   - simulate $s_{t_j,i}$ ,
   - update disactivation boolean $D_i$
3. compute the mean of the discounted payoff $\frac{1}{n} \sum_{i=1}^{n} e^{-rT} (s_{T,i} - K)_+ \overline{D}_i$,

where $n$ is the simulation number, $d$ the point number for the grid of time and $\overline{D}_i$ the opposite of boolean $D_i$.

In the following, we set $T = 1$, $r = 5\%$, $s_{t_0} = 100$, $H = K = 50$, $d = 250$ and $\sigma = 20\%$. We test crude Monte Carlo methods with Park Miller and SF-Mersenne Twister generators and a quasi-Monte Carlo method with (multidimensional) Torus algoritm on the figure 6.

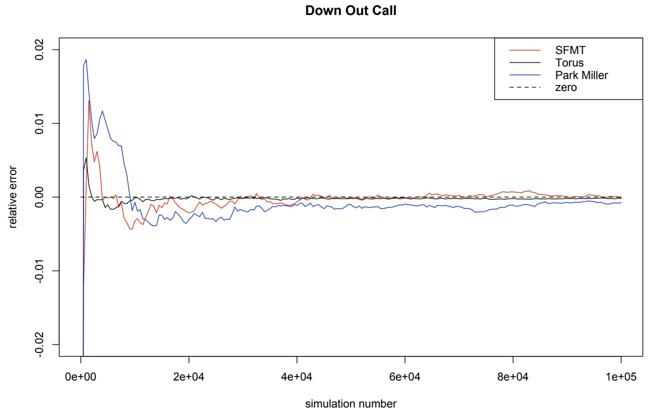


Figure 6: Error function for Down Out Call

One may wonder why the Torus algorithm is still the best (on this example). We use the $d$-dimensional Torus sequence. Thus for time $t_j$, the simulated underlying assets $(s_{t_j,i})_i$ are computed with the sequence $(i\{\sqrt{p_j}\})_i$. Thanks to the linear independence of the Torus sequence over the rationals[3], we guarantee a non-correlation of Torus quasi-random numbers.

However, these results do **not** prove the Torus algorithm is always better than traditional Monte Carlo. The results are sensitive to the barrier level $H$, the strike price $X$ (being in or out the money has a strong impact), the asset volatility $\sigma$ and the time point number $d$.

[1]DOC is disactived when the underlying asset hits the barrier.

[2]created by Wuertz et al. (2007*a*).

[3]i.e. for $k \neq j, \forall i, (i\{\sqrt{p_j}\})_i$ and $(i\{\sqrt{p_k}\})_i$ are linearly independent over $\mathbb{Q}$.

Actuaries or readers with actuarial background can find an example of actuarial applications of QMC methods in Albrecher et al. (2003). This article focuses on simulation methods in ruin models with non-linear dividend barriers.

# 5 Random generation tests

Tests of random generators aim to check if the output $u_1, \dots, u_n, \dots$ could be considered as independent and identically distributed (i.i.d.) uniform variates for a given confidence level. There are two kinds of tests of the uniform distribution: first on the interval $]0,1[$, second on the binary set $\{0,1\}$. In this note, we only describe tests for $]0,1[$ outputs (see L'Ecuyer & Simard (2007) for details about these two kind of tests).

Some RNG tests can be two-level tests, i.e. we do not work directly on the RNG output $u_i$'s but on a function of the output such as the spacings (coordinate difference of the sorted sample).

## 5.1 Test on one sequence of $n$ numbers

### 5.1.1 Goodness of Fit

Goodness of Fit tests compare the empirical cumulative distribution function (cdf) $\mathbb{F}_n$ of $u_i$'s with a specific distribution ($\mathcal{U}(0,1)$ here). The most known test are Kolmogorov-Smirnov, Crámer-von Mises and Anderson-Darling tests. They use different norms to quantify the difference between the empirical cdf $\mathbb{F}_n$ and the true cdf $F_{\mathcal{U}_{(0,1)}}$.

- Kolmogorov-Smirnov statistic is
$$K_n = \sqrt{n} \sup_{x\in\mathbb{R}} \left| \mathbb{F}_n(x) - F_{\mathcal{U}_{(0,1)}}(x) \right|,$$
- Crámer-von Mises statistic is
$$W_n^2 = n \int_{-\infty}^{+\infty} \left( \mathbb{F}_n(x) - F_{\mathcal{U}_{(0,1)}}(x) \right)^2 dF_{\mathcal{U}_{(0,1)}}(x),$$
- and Anderson-Darling statistic is
$$A_n^2 = n \int_{-\infty}^{+\infty} \frac{\left( \mathbb{F}_n(x) - F_{\mathcal{U}_{(0,1)}}(x) \right)^2 dF_{\mathcal{U}_{(0,1)}}(x)}{F_{\mathcal{U}_{(0,1)}}(x)(1 - F_{\mathcal{U}_{(0,1)}}(x))}.$$

Those statistics can be evaluated empirically thanks to the sorted sequence of $u_i$'s. But we will not detail any further those tests, since according to L'Ecuyer & Simard (2007) they are not powerful for random generation testing.

### 5.1.2 The gap test

The gap test investigates for special patterns in the sequence $(u_i)_{1\leq i\leq n}$. We take a subset $[l,u] \subset [0,1]$ and compute the 'gap' variables with

$$G_i = \begin{cases} 1 & \text{if } l \leq U_i \leq u \\ 0 & \text{otherwise.} \end{cases}$$

The probability $p$ that $G_i$ equals to 1 is just the $u - l$ (the Lebesgue measure of the subset). The test computes the length of zero gaps. If we denote by $n_j$ the number of zero gaps of length $j$.

The chi-squared statistic of a such test is given by
$$S = \sum_{j=1}^{m} \frac{(n_j - np_j)^2}{np_j},$$
where $p_j = (1-p)^2 p^j$ is the probability that the length of gaps equals to $j$; and $m$ the max number of lengths. In theory $m$ equals to $+\infty$, but in pratice, it is a large integer. We fix $m$ to be at least
$$\left\lfloor \frac{\log(10^{-1}) - 2\log(1-p) - log(n)}{\log(p)} \right\rfloor,$$
in order to have lengths whose appearance probabilitie is at least 0.1.

### 5.1.3 The order test

The order test looks for another kind of patterns. We test a $d$-tuple, if its components are ordered

equiprobably. For example with $d = 3$, we should have an equal number of vectors $(u_i, u_{i+1}, u_{i+2})_i$ such that

- $u_i < u_{i+1} < u_{i+2}$,
- $u_i < u_{i+2} < u_{i+1}$,
- $u_{i+1} < u_i < u_{i+2}$,
- $u_{i+1} < u_{i+2} < u_i$,
- $u_{i+2} < u_i < u_{i+1}$
- and $u_{i+1} < u_{i+2} < u_i$.

For some $d$, we have $d!$ possible orderings of coordinates, which have the same probability to appear $\frac{1}{d!}$. The chi-squared statistic for the order test for a sequence $(u_i)_{1\leq i\leq n}$ is just

$$S = \sum_{j=1}^{d!} \frac{(n_j - m\frac{1}{d!})^2}{m\frac{1}{d!}},$$

where $n_j$'s are the counts for different orders and $m = \frac{n}{d}$. Computing $d!$ possible orderings has an exponential cost, so in practive $d$ is small.

#### 5.1.4 The frequency test

The frequency test works on a serie of ordered contiguous integers ($J = [i_1, \dots, i_l] \cap \mathbb{Z}$). If we denote by $(n_i)_{1\leq i\leq n}$ the sample number of the set $I$, the expected number of integers equals to $j \in J$ is

$$\frac{1}{i_l - i_1 + 1} \times n,$$

which is independent of $j$. From this, we can compute a chi-squared statistic

$$S = \sum_{j=1}^{l} \frac{(\mathrm{Card}(n_i = i_j) - m)^2}{m},$$

where $m = \frac{n}{d}$.

### 5.2 Tests based on multiple sequences

Under the i.i.d. hypothesis, a vector of output values $u_i, \dots, u_{i+t-1}$ is uniformly distributed over the unit hypercube $[0, 1]^t$. Tests based on multiple sequences partition the unit hypercube into cells and compare the number of points in each cell with the expected number.

#### 5.2.1 The serial test

The most intuitive way to split the unit hypercube $[0, 1]^t$ into $k = d^t$ subcubes. It is achieved by splitting each dimension into $d > 1$ pieces. The volume (i.e. a probability) of each cell is just $\frac{1}{k}$.

The associated chi-square statistic is defined as

$$S = \sum_{j=1}^{m} \frac{(N_j - \lambda)^2}{\lambda},$$

where $N_j$ denotes the counts and $\lambda = \frac{n}{k}$ their expectation.

#### 5.2.2 The collision test

The philosophy is still the same: we want to detect some pathological behavior on the unit hypercube $[0, 1]^t$. A collision is defined as when a point $v_i = (u_i, \dots, u_{i+t-1})$ falls in a cell where there are already points $v_j$'s. Let us note $C$ the number of collisions

The distribution of collision number $C$ is given by

$$P(C = c) = \prod_{i=0}^{n-c-1} \frac{k-i}{k} \frac{1}{k^c}\, {}_2S_n^{n-c},$$

where ${}_2S_n^k$ denotes the Stirling number of the second kind[1] and $c = 0, \dots, n-1$.

But we cannot use this formula for large $n$ since the Stirling number need $O(n \log(n))$ time to be computed. As L'Ecuyer et al. (2002) we use a Gaussian approximation if $\lambda = \frac{n}{k} > \frac{1}{32}$ and $n \geq 2^8$, a Poisson approximation if $\lambda < \frac{1}{32}$ and the exact formula otherwise.

[1] they are defined by ${}_2S_n^k = k \times {}_2S_{n-1}^k + {}_2S_{n-1}^{k-1}$ and ${}_2S_n^1 = {}_2S_n^n = 1$. For example go to wikipedia.

The normal approximation assumes $C$ follows a normal distribution with mean $m = n - k + k\left(\frac{k-1}{k}\right)^n$ and variance very complex (see L'Ecuyer & Simard (2007)). Whereas the Poisson approximation assumes $C$ follows a Poisson distribution of parameter $\frac{n^2}{2k}$.

#### 5.2.3 The $\phi$-divergence test

There exist generalizations of these tests where we take a function of counts $N_j$, which we called $\phi$-divergence test. Let $f$ be a real valued function. The test statistic is given by

$$\sum_{j=0}^{k-1} f(N_j).$$

We retrieve the collision test with $f(x) = (x-1)_+$ and the serial test with $f(x) = \frac{(x-\lambda)^2}{\lambda}$. Plenty of statistics can be derived, for example if we want to test the number of cells with at least $b$ points, $f(x) = \mathbb{1}_{(x=b)}$. For other statistics, see L'Ecuyer et al. (2002).

#### 5.2.4 The poker test

The poker test is a test where cells of the unit cube $[0,1]^t$ do not have the same volume. If we split the unit cube into $d^t$ cells, then by regrouping cells with left hand corner having the same number of distinct coordinates we get the poker test. In a more intuitive way, let us consider a hand of $k$ cards from $k$ different cards. The probability to have exactly $c$ different cards is

$$P(C = c) = \frac{1}{k^k} \frac{k!}{(k-c)!} \, {}_2S_k^c,$$

where $C$ is the random number of different cards and ${}_2S_n^d$ the second-kind Stirling numbers. For a demonstration, go to Knuth (2002).

## 6 Description of RNG test functions

In this section, we will give usage examples of RNG test functions, in a similar way as section 4 illustrates section 2 - two first sub-sections. The last sub-section focuses on detecting a particular RNG.

```
> par(mfrow = c(2,1))
> hist(SFMT(10^3), 100)
> hist(torus(10^3), 100)
```

### 6.1 Test on one sequence of $n$ numbers

Goodness of Fit tests are already implemented in R with the function `ks.test` for Kolmogorov-Smirnov test and in package `adk` for Anderson-Darling test. In the following, we will focus on one-sequence test implemented in `randtoolbox`.

#### 6.1.1 The gap test

The function `gap.test` implements the gap test as described in sub-section 5.1.2. By default, lower and upper bound are $l = 0$ and $u = 0.5$, just as below.

```
> gap.test(runif(1000))

          Gap test

chisq stat = 15, df = 10
, p-value = 0.13

 (sample size : 1000)

length observed freq theoretical freq
```

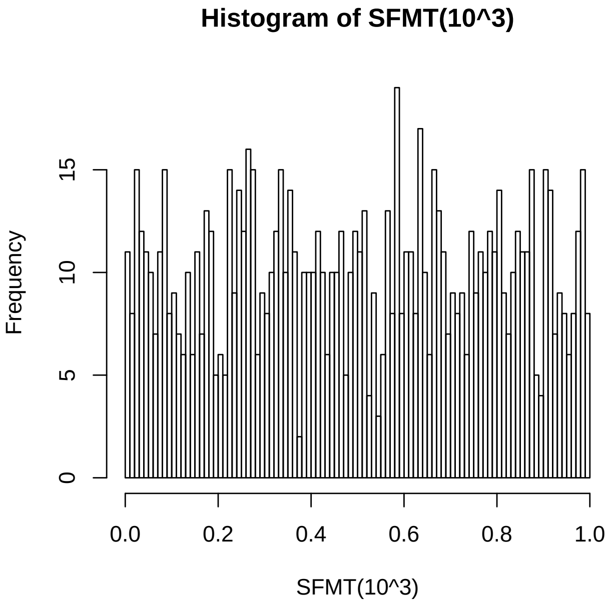


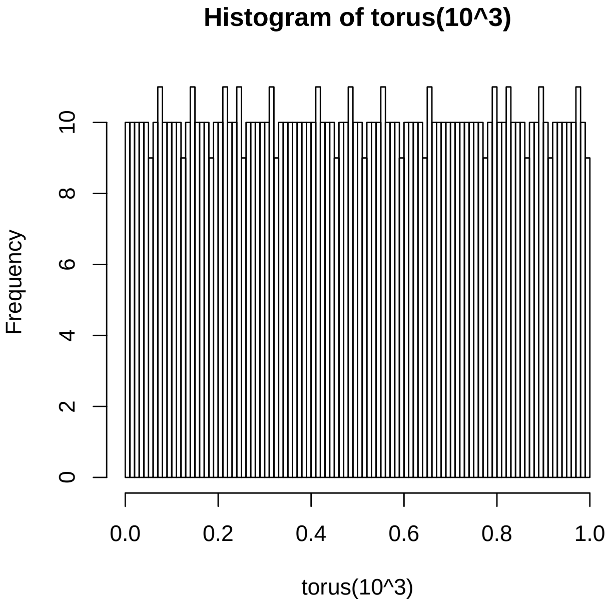


```
1           115           125
2           58           62
3           26           31
4           15           16
5           8           7.8
6           10           3.9
7           3           2
8           1           0.98
9           0           0.49
10           1           0.24
11           0           0.12
```

If you want $l = 1/3$ and $u = 2/3$ with a SFMT sequence, you just type

```
> gap.test(SFMT(1000), 1/3, 2/3)
```

### 6.1.2 The order test

The Order test is implemented in function `order.test` for $d$-uples when $d = 2, 3, 4, 5$. A typical call is as following

```
> order.test(runif(4000), d=4)

         Order test

chisq stat = 25, df = 23
, p-value = 0.33

 (sample size : 1000)

observed number  54 41 38 41 42 50
 26 55 49 39 40 37 46 41 43 39 32 43
 39 41 32 44 50 38

expected number  42
```

Let us notice that the sample length must be a multiple of dimension $d$, see sub-section 5.1.3.

### 6.1.3 The frequency test

The frequency test described in sub-section 5.1.4 is just a basic equi-distribution test in $[0, 1]$ of the generator. We use a sequence integer to partition the unit interval and test counts in each sub-interval.

```
> freq.test(runif(1000), 1:4)

         Frequency test

chisq stat = 1.3, df = 3
, p-value = 0.73

 (sample size : 1000)

observed number  251 252 261 236

expected number  250
```

## 6.2 Tests based on multiple sequences

Let us study the serial test, the collision test and the poker test.

### 6.2.1 The serial test

Defined in sub-section 5.2.1, the serial test focuses on the equidistribution of random numbers in the unit hypercube $[0,1]^t$. We split each dimension of the unit cube in $d$ equal pieces. Currently in function `serial.test`, we implement $t = 2$ and $d$ fixed by the user.

```
> serial.test(runif(3000), 3)

         Serial test

chisq stat = 4.6, df = 8
, p-value = 0.8

 (sample size : 3000)

observed number  158 151 183 165
 162 164 174 176 167

expected number  167
```

In newer version, we will add an argument $t$ for the dimension.

### 6.2.2 The collision test

The exact distribution of collision number costs a lot of time when sample size and cell number are large (see sub-section 5.2.2). With function `coll.test`, we do not yet implement the normal approximation.

The following example tests Mersenne-Twister algorithm (default in R) and parameters implying the use of the exact distribution (i.e. $n < 2^8$ and $\lambda > 1/32$).

```
> coll.test(runif, 2^7, 2^10, 1)

         Collision test

chisq stat = 20, df = 17
, p-value = 0.27

 exact distribution
(sample number : 1000/sample size : 128
 / cell number : 1024)

collision observed expected
number    count    count

  1      1      2.3
  2      10      10
  3      30      29
  4      54      62
  5      106      102
  6      128      138
  7      162      156
  8      130      151
  9      157      126
```

```
  10      87      93
  11      64      61
  12      38      36
  13      21      19
  14      8      8.9
  15      3      3.9
  16      0      1.5
  17      0      0.56
  18      1      0.19
```

When the cell number is far greater than the sample length, we use the Poisson approximation (i.e. $\lambda < 1/32$). For example with `congruRand` generator we have

```
> coll.test(congruRand, 2^8, 2^14, 1)

        Collision test

chisq stat = 33, df = 10
, p-value = 0.00028

 Poisson approximation
(sample number : 1000/sample size : 256
 / cell number : 16384)

collision observed expected
number    count    count

  0      136      135
  1      284      271
  2      261      271
  3      185      180
  4      83      90
  5      33      36
  6      8      12
  7      7      3.4
  8      2      0.86
  9      0      0.19
  10      1      0.038
```

Note that the Normal approximation is not yet implemented and those two approximations are not valid when some expected collision numbers are below 5.

### 6.2.3 The poker test

Finally the function `poker.test` implements the poker test as described in sub-section 5.2.4. We implement for any "card number" denoted by $k$. A typical example follows

```
> poker.test(SFMT(10000))

        Poker test

chisq stat = 1.8, df = 4
, p-value = 0.78

 (sample size : 10000)

observed number  1 187 968 765 79

expected number  3.2 192 960 768 77
```

## 6.3 Hardness of detecting a difference from truly random numbers

Random number generators typically have an internal memory of fixed size, whose content is called the internal state. Since the number of possible internal states is finite, the output sequence is periodic. The length of this period is an important parametr of the random number generator. For example, Mersenne-Twister generator, which is the default in R, has its internal state stored in 624 unsigned integers of 32 bits each. So, the internal state consists of 19968 bits, but only 19937 are used. The period length is $2^{19937} - 1$, which is a Mersenne prime.

Large period is not the only important parameter of a generator. For a good generator, it is not computationally easy to distinguish the output of the generator from truly random numbers, if the seed or the index in the sequence is not known.

Generators, which are good from this point of view, are used for cryptographic purposes. These generators are chosen so that there is no known procedure, which could distinguish their output from truly random numbers within practically available computation time. For simulations, this requirement is usually relaxed.

However, even for simulation purposes, considering the hardness of distinguishing the generated numbers from truly random ones is a good measure of the quality of the generator. In particular, the well-known empirical tests of random number generators such as Diehard[1] or TestU01 L'Ecuyer & Simard (2007) are based on comparing statistics computed for the generator with their values expected for truly random numbers. Consequently, if a generator fails an empirical test, then the output of the test provides a way to distinguish the generator from the truly random numbers.

Besides of general purpose empirical tests constructed without the knowledge of a concrete generator, there are tests specific to a given generator, which allow to distinguish this particular generator from truly random numbers.

An example of a generator, whose output may easily be distinguished from truly random numbers, is the older version of Wichmann-Hill from 1982. For this generator, we can even predict the next number in the sequence, if we know the last already generated one. Verifying such a predicition is easy and it is, of course, not valid for truly random numbers. Hence, we can easily distinguish the output of the generator from truly random numbers. An implementation of this test in R derived from McCullough (2008) is as follows.

```
> wh.predict <- function(x)
+ {
+     M1 <- 30269
+     M2 <- 30307
+     M3 <- 30323
+     y <- round(M1*M2*M3*x)
+     s1 <- y %% M1
+     s2 <- y %% M2
+     s3 <- y %% M3
+     s1 <- (171*26478*s1) %% M1
+     s2 <- (172*26070*s2) %% M2
+     s3 <- (170*8037*s3) %% M3
+     (s1/M1 + s2/M2 + s3/M3) %% 1
+ }
> RNGkind("Wichmann-Hill")
> xnew <- runif(1)
> err <- 0
> for (i in 1:1000) {
+     xold <- xnew
+     xnew <- runif(1)
+     err <- max(err, abs(wh.predict(xold) - x
+ }
> print(err)

[1] 0
```

[1]The Marsaglia Random Number CDROM including the Diehard Battery of Tests of Randomness, Research Sponsored by the National Science Foundation (Grants DMS-8807976 and DMS-9206972), copyright 1995 George Marsaglia.

The printed error is 0 on some machines and less than $5 \cdot 10^{-16}$ on other machines. This is clearly different from the error obtained for truly random numbers, which is close to 1.

The requirement that the output of a random number generator should not be distinguishable from the truly random numbers by a simple computation, is also directly related to the way, how a generator is used. Typically, we use the generated numbers as an input to a computation and we expect that the distribution of the output (for different seeds or for different starting indices in the sequence) is the same as if the input are truly random numbers. A failure of this assumption implies that observing the output of the computation allows to distinguish the output from the generator from truly random numbers. Hence, we want to use a generator, for which we may expect that the calculations used in the intended application cannot distinguish its output from truly random numbers.

In this context, it has to be noted that many

of the currently used generators for simulations can be distinguished from truly random numbers using the arithmetic mod 2 applied to individual bits of the output numbers. This is true for Mersenne Twister, SFMT and also all WELL generators. The basis for tolerating this is based on two facts.

First, the arithmetic mod 2 and extracting individual bits of real numbers is not directly used in typical simulation problems and real valued functions, which represent these operations are extremely discontinuous and such functions also do not typically occur in simulation problems. Another reason is that we need to observe quite a long history of the output to detect the difference from true randomness. For example, for Mersenne Twister, we need 624 consecutive numbers.

On the other hand, if we use a cryptographically strong pseudorandom number generator, we may avoid a difference from truly random numbers under any known efficient procedure. Such generators are typically slower than Mersenne Twister type generators. The factor of slow down may be, for example, 5. If the simulation problem requires intensive computation besides the generating random numbers, using slower, but better, generator may imply only negligible slow down of the computation as a whole.

# 7 Calling the functions from other packages

In this section, we briefly present what to do if you want to use this package in your package. This section is mainly taken from package `expm` available on R-forge.

Package authors can use facilities from **randtoolbox** in two ways:

- call the R level functions (e.g. `torus`) in R code;
- if random number generators are needed in C, call the routine `torus`,...

Using R level functions in a package simply requires the following two import directives:

```
Imports: randtoolbox
```

in file `DESCRIPTION` and

```
import(randtoolbox)
```

in file `NAMESPACE`.

Accessing C level routines further requires to prototype the function name and to retrieve its pointer in the package initialization function `R_init_`*`pkg`*, where *`pkg`* is the name of the package.

For example if you want to use `torus` C function, you need

```
void (*torus)(double *u, int nb, int dim,
int *prime, int ismixed, int usetime);

void R_init_pkg(DllInfo *dll)
{
torus = (void (*) (double, int, int,
int, int, int)) \
R_GetCCallable("randtoolbox", "torus");
}
```

See file `randtoolbox.h` to find headers of RNGs. Examples of *C* calls to other functions can be found in this package with the WELL RNG functions.

The definitive reference for these matters remains the *Writing R Extensions* manual, page 20 in sub-section "specifying imports exports" and page 64 in sub-section "registering native routines".

# References


Albrecher, H., Kainhofer, R. & Tichy, R. E. (2003), 'Simulation methods in ruin models with non-linear dividend barriers', *Mathematics and Computers in Simulation* **62**, 277–287. 21

Antonov, I. & Saleev, V. (1979), 'An economic method of computing lp sequences', *USSR Comp. Mathematics and Mathematical Physics 19* pp. 252–256. 10

Black, F. & Scholes, M. (1973), 'The pricing of options and corporate liabilities', *Journal of Political Economy* **81**(3). 19

Bratley, P. & Fox, B. (1988), 'Algorithm 659: Implementing sobol's quasi-random sequence generators', *ACM Transactions on Mathematical Software* **14**(88-100). 10

Bratley, P., Fox, B. & Niederreiter, H. (1992), 'Implementation and tests of low discrepancy sequences', *ACM Transactions Mode; Comput. Simul.* **2**(195-213). 10

Eddelbuettel, D. (2007), *random: True random numbers using random.org.*
**URL:** *http://www.random.org* 2

Genz, A. (1982), 'A lagrange extrapolation algorithm for sequences of approximations to multiple integrals', *SIAM Journal on scientific computing* **3**, 160–172. 19

Hong, H. & Hickernell, F. (2001), Implementing scrambled digital sequences. preprint. 10

Jäckel, P. (2002), *Monte Carlo methods in finace*, John Wiley & Sons. 7

Joe, S. & Kuo, F. (1999), Remark on algorithm 659: Implementing sobol's quasi-random sequence generator. Preprint. 10

Knuth, D. E. (2002), *The Art of Computer Programming: seminumerical algorithms*, Vol. 2, 3rd edition edn, Massachusetts: Addison-Wesley. 3, 4, 13, 23

L'Ecuyer, P. (1990), 'Random numbers for simulation', *Communications of the ACM* **33**, 85–98. 2, 3, 4

L'Ecuyer, P. & Simard, R. (2007), 'Testu01: A c library for empirical testing of random number generators', *ACM Trans. on Mathematical Software* **33**(4), 22. 21, 23, 27

L'Ecuyer, P., Simard, R. & Wegenkittl, S. (2002), 'Sparse serial tests of uniformity for random number generations', *SIAM Journal on scientific computing* **24**(2), 652–668. 22, 23

Marsaglia, G. (1994), 'Some portable very-long-period random number generators', *Computers in Physics* **8**, 117–121. 13

Matsumoto, M. & Nishimura, T. (1998), 'Mersenne twister: A 623-dimensionnally equidistributed uniform pseudorandom number generator', *ACM Trans. on Modelling and Computer Simulation* **8**(1), 3–30. 4, 6

Matsumoto, M. & Saito, M. (2008), *SIMD-oriented Fast Mersenne Twister: a 128-bit pseudorandom number generator*, Monte Carlo and Quasi-Monte Carlo Methods 2006, Springer. 6, 15

McCullough, B. D. (2008), 'Microsoft excel's 'not the wichmann–hill' random number generators', *Computational Statistics and Data Analysis* **52**, 4587–4593. 11, 27

Namee, J. M. & Stenger, F. (1967), 'Construction of ful ly symmetric numerical integration formulas', *Numerical Mathatematics* **10**, 327–344. 19

Niederreiter, H. (1978), 'Quasi-monte carlo methods and pseudo-random numbers', *Bulletin of the American Mathematical Society* **84**(6). 2, 7, 8, 9, 11

Niederreiter, H. (1992), *Random Number Generation and Quasi-Monte Carlo Methods*, SIAM, Philadelphia. 7

Panneton, F., L'Ecuyer, P. & Matsumoto, M. (2006), 'Improved long-period generators based on linear recurrences modulo 2', *ACM Trans. on Mathematical Software* **32**(1), 1–16. 5

Papageorgiou, A. & Traub, J. (2000), 'Faster evaluation of multidimensional integrals', *arXiv:physics/0011053v1* p. 10. 19

Park, S. K. & Miller, K. W. (1988), 'Random number generators: good ones are hard to find.', *Association for Computing Machinery* **31**(10), 1192–2001. 2, 3, 4, 13

Patterson, T. (1968), 'The optimum addition of points to quadrature formulae', *Mathematics of Computation* pp. 847–856. 19

Planchet, F., Thérond, P. & Jacquemin, J. (2005), *Modèles Financiers En Assurance*, Economica. 14

Press, W., Teukolsky, W., William, T. & Brian, P. (1996), *Numerical Recipes in Fortran*, Cambridge University Press. 10

Rubinstein, M. & Reiner, E. (1991), 'Unscrambling the binary code', *Risk Magazine* **4**(9). 20

Wichmann, B. A. & Hill, I. D. (1982), 'Algorithm as 183: An efficient and portable pseudo-random number generator', *Applied Statistics* **31**, 188–190. 13

Wuertz, D., many others & see the SOURCE file (2007*a*), *fExoticOptions: Rmetrics - Exotic Option Valuation.*
**URL:** *http://www.rmetrics.org* 20

Wuertz, D., many others & see the SOURCE file (2007*b*), *fOptions: Rmetrics - Basics of Option Valuation.*
**URL:** *http://www.rmetrics.org* 19
