# Supplementary material for: The relationship between leaf area growth and biomass accumulation in Arabidopsis thaliana
Source: Front Plant Sci. 2015 Apr 9;6:167. doi: 10.3389/fpls.2015.00167 (PMC4391269; doi:10.3389/fpls.2015.00167)
Supplement: Supplementary file 4 [file DataSheet1.ZIP › R_libs - parallization.zip!/R_libs/randtoolbox/doc/index.html]

R: Vignettes and other documentation

# Vignettes and other documentation

---

## Vignettes from package 'randtoolbox'

|  |  |  |  |  |  |
| --- | --- | --- | --- | --- | --- |
| randtoolbox::fullpres |  | A note on random number generation | PDF | source | R code |
| randtoolbox::shortintro |  | Quick introduction of randtoolbox | PDF | source | R code |
