# Supplementary material for: The relationship between leaf area growth and biomass accumulation in Arabidopsis thaliana
Source: Front Plant Sci. 2015 Apr 9;6:167. doi: 10.3389/fpls.2015.00167 (PMC4391269; doi:10.3389/fpls.2015.00167)
Supplement: Supplementary file 4 [file DataSheet1.ZIP › R_libs - parallization.zip!/R_libs/randtoolbox/doc/shortintro.pdf]

Quick introduction of **randtoolbox**

Christophe Dutang and Petr Savicky

September 2009

Random simulation or Monte-Carlo methods rely on the fact we have access to random numbers. Even if nowadays having random sequence is no longer a problem, for many years producing random numbers was a big challenge. According to Ripley (1990), simulation started in 1940s with physical devices. Using physical phenomena to get random numbers is referred in the literature as true randomness.

However, in our computers, we use more frequently pseudo-random numbers. These are defined as deterministic sequences, which mimic a sequence of i.i.d. random numbers chosen from the uniform distribution on the interval $[0, 1]$. Random number generators used for this purpose receive as input an initial information, which is called a user specified seed, and allow to obtain different output sequences of numbers from $[0, 1]$ depending on the seed. If no seed is supplied by the user, we use the machine time to initiate the sequence.

Since we use pseudo-random numbers as a proxy for random numbers, an important question is, which properties the RNG should have to work as a good replacement of the truly random numbers. Essentially, we need that the applications, which we have, produce the same results, or results from the same distribution, no matter, whether we use pseudo-random numbers or truly random numbers. Hence, the required properties may be formulated in terms of computational indistinguishability of the output of the generator from the truly random numbers, if the seed is not known. The corresponding mathematical theory is developed in complexity theory, see `http://www.wisdom.weizmann.ac.il/~oded/c-indist.html`.

The best known random number generators are used for cryptographic purposes. These generators are chosen so that there is no known procedure, which could distinguish their output from truly random numbers within practically available computation time, if the seed is not known. For simulations, this requirement is usually relaxed. However, even for simulation purposes, considering the hardness of detecting the difference between the generated numbers and truly random ones is a good measure of the quality of the generator. In particular, the well-known empirical tests of random number generators such as Diehard[1] or TestU01 L'Ecuyer & Simard (2007) are based on relatively easy to compute statistics, which allow to distinguish the output of bad generators from truly random numbers. More about this may be found in section Examples of distinguishing from truly random numbers.

A simple parameter of a generator is its period. Recent generators have huge periods, which cannot be exhausted by any practical computation. Another parameter, suitable mainly for linear generators, is so called equidistribution. This parameter measures the uniformity of several most significant bits of several consecutive numbers in the sequence over the whole period. If a generator has good equidistribution, then we have a reasonable guarantee of practical independence of several consecutive numbers in the sequence. For linear generators, determining equidistribution properties may be done by efficient algebraic algorithms and does not need to really generate the whole period.

Ripley (1990) lists the following properties

- output numbers are almost uniformly distributed,
- output numbers are independent,
- the period between two identical numbers is sufficiently long,
- unless a seed is given, output numbers should be unpredictable.

[1]The Marsaglia Random Number CDROM including the Diehard Battery of Tests of Randomness, Research Sponsored by the National Science Foundation (Grants DMS-8807976 and DMS-9206972), copyright 1995 George Marsaglia.

The statistical software R provides several random number generators described in '?RNGkind()'. The default generator is called Mersenne-Twister and achieves high quality, although it fails some tests based on XOR operation. Still, there are reasons to provide better and more recent RNGs as well as classic statistical tests to quantify their properties. The rest of this chapter is two-folded: first we present the use of RNGs through the `runif()` interface, second we present the same use with dedicated functions (not modifying base R default RNGs). See the overall man page with the command `?randtoolbox`.

1 The `runif` interface

In R, the default setting for random generation are (i) uniform numbers are produced by the Mersenne-Twister algorithm and (ii) normal numbers are computing through the numerical inversion of the standard normal distribution function. This can be checked by the following code

```
> RNGkind()
```

```
[1] "Wichmann-Hill" "Inversion"
```

The function `RNGkind()` can also be used to set other RNGs, such as Wichmann-Hill, Marsaglia-Multicarry, Super-Duper, Knuth-TAOCP or Knuth-TAOCP-2002 plus a user-supplied RNG. See the help page for details.

Random number generators provided by R extension packages are set using `RNGkind("user-supplied")`. The package **randtoolbox** assumes that this function is not called by the user directly. Instead, it is called from the functions `set.generator()` and `put.description()` used for setting some of a larger collection of the supported generators.

The function `set.generator()` eases the process to set a new RNG in R. Here is one short example on how to use `set.generator()` (see the man page for detailed explanations).

```
> RNGkind()
```

```
[1] "Wichmann-Hill" "Inversion"
```

```
> library(randtoolbox)
> paramParkMiller <- c(mod=2^31-1, mult=16807, incr=0)
> set.generator(name="congruRand", parameters=paramParkMiller, seed=1)
> get.description()
```

```
18446744073709551616184467440737095516161844674407370955161618446744073709551616$name
[1] "congruRand"
```

```
$parameters
                  mod
"18446744073709551616"
                 mult
"18446744073709551616"
                 incr
"18446744073709551616"

$state
                 seed
"18446744073709551616"

$authors
[1] "Unknown"
```

```
> RNGkind()
```

```
[1] "user-supplied" "Inversion"
```

```
> runif(10)
```

```
 [1] 7.8e-06 1.3e-01 7.6e-01 4.6e-01
 [5] 5.3e-01 2.2e-01 4.7e-02 6.8e-01
 [9] 6.8e-01 9.3e-01
```

Random number generators in **randtoolbox** are represented at the R level by a list containing mandatory components `name, parameters, state` and possibly an optional component `authors`. The function `set.generator()` internally creates this list from the user supplied information and then runs `put.description()` on this list in order to really initialize the generator for the functions `runif()` and `set.seed()`. If `set.generator()` is called with the parameter `only.dsc=TRUE`, then the generator is not initialized and only its description is created. If the generator is initialized, then the function `get.description()` may be used to get the actual state of the generator, which may be stored and used later in `put.description()` to continue the sequence of the random numbers from the point, where `get.description()` was called. This may be used, for example, to alternate between the streams of random numbers generated by different generators.

From the `runif()` interface, you can use any other linear congruential generator with modulus at most 2^{64} and multiplier, which is either a power of 2 or the product of the modulus and the multiplier is at most 2^{64}. The current version of the package also allows to use Well-Equidistributed Long-period Linear generators (WELL).

To get back to the original setting of RNGs in R, we just need to call `set.generator` with `default` RNG.

```
> set.generator("default")
> RNGkind()
```

```
[1] "Mersenne-Twister"
[2] "Inversion"
```

2 Dedicated functions

The other way to use RNGs is to directly use dedicated functions. For instance to get the previous example, we can simply use

```
> setSeed(1)
> congruRand(10, mod = 2^31-1, mult = 16807, incr = 0)
```

```
 [1] 7.8e-06 1.3e-01 7.6e-01 4.6e-01
 [5] 5.3e-01 2.2e-01 4.7e-02 6.8e-01
 [9] 6.8e-01 9.3e-01
```

where `setSeed` function initiates the seed for RNGs implemented in **randtoolbox** and `congruRand` calls the congruential generator.

They are many other RNGs provided by RNGs in addition to linear congruential generator, WELL generators, SFMersenne-Twister generators and Knuth-TAOCP double version. See `?pseudo.randtoolbox` for details.

This package also implements usual quasi random generators such as Sobol or Halton sequences (see `?quasi.randtoolbox`). See the second chapter for an explanation on quasi RNGs.

References

L'Ecuyer, P. & Simard, R. (2007), 'Testu01: A c library for empirical testing of random number generators', *ACM Trans. on Mathematical Software* **33**(4), 22. 2

Ripley, B. D. (1990), *Stochastic Simulation*, John Wiley & Sons. 2
