# Supplementary material for: The relationship between leaf area growth and biomass accumulation in Arabidopsis thaliana
Source: Front Plant Sci. 2015 Apr 9;6:167. doi: 10.3389/fpls.2015.00167 (PMC4391269; doi:10.3389/fpls.2015.00167)
Supplement: Supplementary file 4 [file DataSheet1.ZIP › R_libs - parallization.zip!/R_libs/randtoolbox/html/00Index.html]

R: toolbox for pseudo and quasi random number generation and RNG
tests.

# toolbox for pseudo and quasi random number generation and RNG tests.

---

## Documentation for package ‘randtoolbox’ version 1.15

- DESCRIPTION file.
- User guides, package vignettes and other documentation.
- Package NEWS.

## Help Pages

|  |  |
| --- | --- |
| randtoolbox-package | General remarks on toolbox for pseudo and quasi random number generation |
| auxiliary | Auxiliary functions for 'randtoolbox' package. |
| coll.test | the Collision test |
| coll.test.sparse | the Collision test |
| congruRand | Toolbox for pseudo and quasi random number generation |
| freq.test | the Frequency test |
| gap.test | the Gap test |
| get.description | Functions for using runif() and rnorm() with randtoolbox generators |
| get.primes | Get primes for quasi random number generation |
| getWELLState | Get the state of a WELL generator implemented in randtoolbox package. |
| halton | Toolbox for quasi random number generation |
| knuthTAOCP | Toolbox for pseudo and quasi random number generation |
| order.test | the Order test |
| permut | Auxiliary functions for 'randtoolbox' package. |
| poker.test | the Poker test |
| pseudo.randtoolbox | Toolbox for pseudo and quasi random number generation |
| pseudoRNG | Toolbox for pseudo and quasi random number generation |
| put.description | Functions for using runif() and rnorm() with randtoolbox generators |
| quasi.randtoolbox | Toolbox for quasi random number generation |
| quasiRNG | Toolbox for quasi random number generation |
| randtoolbox | General remarks on toolbox for pseudo and quasi random number generation |
| rngWELLScriptR | An implementation of the recurrence of WELL generators in R language |
| runif.halton | Toolbox for quasi random number generation |
| runif.sobol | Toolbox for quasi random number generation |
| runifInterface | Functions for using runif() and rnorm() with randtoolbox generators |
| serial.test | the Serial test |
| set.generator | Functions for using runif() and rnorm() with randtoolbox generators |
| setSeed | Toolbox for pseudo and quasi random number generation |
| SFMT | Toolbox for pseudo and quasi random number generation |
| sobol | Toolbox for quasi random number generation |
| stirling | Auxiliary functions for 'randtoolbox' package. |
| torus | Toolbox for quasi random number generation |
| WELL | Toolbox for pseudo and quasi random number generation |
