# Supplementary material for: The relationship between leaf area growth and biomass accumulation in Arabidopsis thaliana
Source: Front Plant Sci. 2015 Apr 9;6:167. doi: 10.3389/fpls.2015.00167 (PMC4391269; doi:10.3389/fpls.2015.00167)
Supplement: Supplementary file 4 [file DataSheet1.ZIP › R_libs - parallization.zip!/R_libs/Rcpp/doc/index.html]

R: Vignettes and other documentation

# Vignettes and other documentation

---

## Vignettes from package 'Rcpp'

|  |  |  |  |  |  |
| --- | --- | --- | --- | --- | --- |
| Rcpp::Rcpp-FAQ |  | Rcpp-FAQ | PDF | source | R code |
| Rcpp::Rcpp-attributes |  | Rcpp-attributes | PDF | source | R code |
| Rcpp::Rcpp-extending |  | Rcpp-extending | PDF | source | R code |
| Rcpp::Rcpp-modules |  | Rcpp-modules | PDF | source | R code |
| Rcpp::Rcpp-package |  | Rcpp-package | PDF | source | R code |
| Rcpp::Rcpp-quickref |  | Rcpp-quickref | PDF | source | R code |
| Rcpp::Rcpp-sugar |  | Rcpp-sugar | PDF | source | R code |
| Rcpp::Rcpp-introduction |  | Rcpp-introduction | PDF | source | R code |
| Rcpp::Rcpp-unitTests |  | Rcpp-unitTests | PDF | source | R code |

## Other files in the doc directory

|  |  |
| --- | --- |
|  | Rcpp.bib |
