# Supplementary material for: The relationship between leaf area growth and biomass accumulation in Arabidopsis thaliana
Source: Front Plant Sci. 2015 Apr 9;6:167. doi: 10.3389/fpls.2015.00167 (PMC4391269; doi:10.3389/fpls.2015.00167)
Supplement: Supplementary file 4 [file DataSheet1.ZIP › R_libs - parallization.zip!/R_libs/Rcpp/doc/Rcpp-FAQ.pdf]

Frequently Asked Questions about **Rcpp**

Dirk Eddelbuettel   Romain François

Rcpp version 0.11.3 as of September 27, 2014

Abstract

This document attempts to answer the most Frequently Asked Questions (FAQ) regarding the **Rcpp** (Eddelbuettel, François, Allaire, Chambers, Bates, and Ushey, 2014; Eddelbuettel and François, 2011; Eddelbuettel, 2013) package.

1 Getting started

1.1 How do I get started ?

If you have **Rcpp** installed, please execute the following command in R to access the introductory vignette (which is a variant of the Eddelbuettel and François (2011) paper) for a detailed introduction:

```
> vignette("Rcpp-introduction")
```

If you do not have **Rcpp** installed, the document should also be available whereever you found this document, *i.e.,* on every mirror of CRAN site.

1.2 What do I need ?

Obviously, R must be installed. **Rcpp** provides a C++ API as an extension to the R system. As such, it is bound by the choices made by R and is also influenced by how R is configured.

In general, the standard environment for building a CRAN package from source (particularly when it contains C or C++ code) is required. This means one needs:

- a development environment with a suitable compiler (see below), header files and required libraries;
- R should be built in a way that permits linking and possibly embedding of R; this is typically ensured by the `-enable-shared-lib` option;
- standard development tools such as `make` etc.

Also see the RStudio documentation on pre-requisites for R package development.

1.3 What compiler can I use ?

On almost all platforms, the GNU Compiler Collection (or `gcc`, which is also the name of its C language compiler) has to be used along with the corresponding `g++` compiler for the C++ language. A minimal suitable version is a final 4.2.* release; earlier 4.2.* were lacking some C++ features (and even 4.2.1, still used on OS X as the last gcc release), has issues).

Generally speaking, the default compilers on all the common platforms are suitable.

Specific per-platform notes:

Windows users need the `Rtools` package from the site maintained by Duncan Murdoch which contains all the required tools in a single package; complete instructions specific to Windows are in the 'R Administration' manual (R Development Core Team, 2013, Appendix D). As of August 2014, it still installs the `gcc/g++` 4.6.* compiler which limits the ability to use modern C++ standards so only `s-std=c++0x` is supported. R 3.1.0 and above detect this and set appropriate flags.

OS X users, as noted in the 'R Administration' manual (R Development Core Team, 2013, Appendix C.4), need to install the Apple Developer Tools (*e.g.*, `Xcode`) (as well as `gfortran` if R or Fortran-using packages are to be built); also see FAQ 2.10 and FAQ 2.16 below. Depending on whether on OS X release before or after Mavericks is used, different additional installation may be needed. Consult the `r-sig-mac` list (and its archives) for (current) details.

Linux user need to install the standard developement packages. Some distributions provide helper packages which pull in all the required packages; the `r-base-dev` package on Debian and Ubuntu is an example.

The `clang` and `clang++` compilers from the LLVM project can also be used. On Linux, they are inter-operable with `gcc` et al. On OS X, they are unfortunately not ABI compatible. The `clang++` compiler is interesting as it emits much more comprehensible error messages than `g++` (though `g++` 4.8 and 4.9 have caught up).

The Intel `icc` family has also been used successfully as its output files can also be combined with those from `gcc`.

1.4 What other packages are useful ?

Additional packages that we have found useful are

inline which is invaluable for direct compilation, linking and loading of short code snippets—but now effectively superseded by the Rcpp Attributes (see FAQ 2.2.2 and FAQ 2.14) feature provided by **Rcpp**;

RUnit is used for unit testing; the package is recommended and will be needed to re-run some of our tests but it is not strictly required during use of **Rcpp**;

rbenchmark to run simple timing comparisons and benchmarks; it is also recommended but not required.

microbenchmark is an alternative for benchmarking.

devtools can help the process of building, compiling and testing a package but it too is entirely optional.

1.5 What licenses can I choose for my code?

The **Rcpp** package is licensed under the terms of the GNU GPL 2 or later, just like R itself. A key goal of the **Rcpp** package is to make extending R more seamless. But by *linking* your code against R (as well as **Rcpp**), the combination is bound by the GPL as well. This is very clearly stated at the FSF website:

> Linking a GPL covered work statically or dynamically with other modules is making a combined work based on the GPL covered work. Thus, the terms and conditions of the GNU General Public License cover the whole combination.

So you are free to license your work under whichever terms you find suitable (provided they are GPL-compatible, see the FSF site for details). However, the combined work will remain under the terms and conditions of the GNU General Public License. This restriction comes from both R which is GPL-licensed as well as from **Rcpp** and whichever other GPL-licensed components you may be linking against.

2 Compiling and Linking

2.1 How do I use Rcpp in my package ?

Rcpp has been specifically designed to be used by other packages. Making a package that uses **Rcpp** depends on the same mechanics that are involved in making any R package that use compiled code — so reading the *Writing R Extensions* manual (R Development Core Team, 2012) is a required first step.

Further steps, specific to **Rcpp**, are described in a separate vignette.

```
> vignette("Rcpp-package")
```

2.2 How do I quickly prototype my code?

There are two toolchains which can help with this:

- The older one is provided by the **inline** package and described in Section 2.2.1.
- Starting with **Rcpp** 0.10.0, the Rcpp Attributes feature (described in Section 2.2.2) offered an even easier alternative via the function `evalCpp`, `cppFunction` and `sourceCpp`.

The next two subsections show an example each.

2.2.1 Using inline

The **inline** package (Sklyar, Murdoch, Smith, Eddelbuettel, and François, 2013) provides the functions `cfunction` and `cxxfunction`. Below is a simple function that uses `accumulate` from the (C++) Standard Template Library to sum the elements of a numeric vector.

```
> fx <- cxxfunction(signature(x = "numeric"),
+     'NumericVector xx(x);
     return wrap(std::accumulate(xx.begin(), xx.end(), 0.0));',
+     plugin = "Rcpp")
> res <- fx(seq(1, 10, by=0.5))
> res
[1] 104.5
```

One might want to use code that lives in a C++ file instead of writing the code in a character string in R. This is easily achieved by using `readLines`:

```
> fx <- cxxfunction(signature(), paste(readLines("myfile.cpp"), collapse="\n"),
+                   plugin = "Rcpp")
```

The `verbose` argument of `cxxfunction` is very useful as it shows how **inline** runs the show.

2.2.2 Using Rcpp Attributes

Rcpp Attributes (Allaire, Eddelbuettel, and François, 2013), and also discussed in FAQ 2.14 below, permits an even easier route to integrating R and C++. It provides three key functions. First, `evalCpp` provide a means to evaluate simple C++ expression which is often useful for small tests, or to simply check if the toolchain is set up correctly. Second, `cppFunction` can be used to create C++ functions for R use on the fly. Third, `Rcpp`sourceCpp can integrate entire files in order to define multiple functions.

The example above can now be rewritten as:

```
> cppFunction('double accu(NumericVector x) {
   return(std::accumulate(x.begin(), x.end(), 0.0));
}')
> res <- accu(seq(1, 10, by=0.5))
> res
[1] 104.5
```

The `cppFunction` parses the supplied text, extracts the desired function names, creates the required scaffolding, compiles, links and loads the supplied code and makes it available under the selected identifier.

Similarly, `sourceCpp` can read in a file and compile, link and load the code therein.

2.3 How do I convert my prototyped code to a package ?

Since release 0.3.5 of **inline**, one can combine FAQ 2.2.1 and FAQ 2.1. See `help("package.skeleton-methods")` once **inline** is loaded and use the skeleton-generating functionality to transform a prototyped function into the minimal structure of a package. After that you can proceed with working on the package in the spirit of FAQ 2.1.

Rcpp Attributes (Allaire et al., 2013) also offers a means to convert functions written using Rcpp Attributes into a function via the `compileAttributes` function; see the vignette for details.

2.4 How do I quickly prototype my code in a package?

The simplest way may be to work directly with a package. Changes to both the R and C++ code can be compiled and tested from the command line via:

```
$ R CMD INSTALL mypkg && Rscript --default-packages=mypkg -e 'someFunction-
ToTickle(3.14)'
```

This first installs the packages, and then uses the command-line tool `Rscript` (which ships with `R`) to load the package, and execute the R expression following the `-e` switch. Such an expression can contain multiple statements separated by semicolons. `Rscript` is available on all three core operating systems.

On Linux, one can also use `r` from the `littler` package by Horner and Eddelbuettel which is an alternative front end to R designed for both `#!` (hashbang) scripting and command-line use. It has slightly faster start-up times than `Rscript`; and both give a guaranteed clean slate as a new session is created.

The example then becomes

```
$ R CMD INSTALL mypkg && r -l mypkg -e 'someFunctionToTickle(3.14)'
```

The `-l` option calls 'suppressMessages(library(mypkg))' before executing the R expression. Several packages can be listed, separated by a comma.

More choice are provide by the **devtools** package, and by using RStudio. See the respective documentation for details.

2.5 But I want to compile my code with R CMD SHLIB !

The recommended way is to create a package and follow FAQ 2.1. The alternate recommendation is to use **inline** and follow FAQ 2.2.1 because it takes care of all the details.

However, some people have shown that they prefer not to follow recommended guidelines and compile their code using the traditional `R CMD SHLIB`. To do so, we need to help `SHLIB` and let it know about the header files that **Rcpp** provides and the C++ library the code must link against.

On the Linux command-line, you can do the following:

```
$ export PKG_LIBS=`Rscript -e "Rcpp:::LdFlags()"`   # if Rcpp older than 0.11.0
$ export PKG_CXXFLAGS=`Rscript -e "Rcpp:::CxxFlags()"`
$ R CMD SHLIB myfile.cpp
```

which first defines and exports two relevant environment variables which `R CMD SHLIB` then relies on. On other operating systems, appropriate settings may have to be used to define the environment variables.

This approach corresponds to the very earliest ways of building programs and can still be found in some deprecated documents (as *e.g.* some of Dirk's older 'Intro to HPC with R' tutorial slides). It is still not recommended as there are tools and automation mechanisms that can do the work for you.

Rcpp versions 0.11.0 or later can do with the definition of `PKG_LIBS` as a user-facing library is no longer needed (and hence no longer shipped with the package). One still needs to set `PKG_CXXFLAGS` to tell R where the **Rcpp** headers files are located.

Once `R CMD SHLIB` has created the dyanmically-loadable file (with extension `.so` on Linux, `.dylib` on OS X or `.dll` on Windows), it can be loaded in an R session via `dyn.load`, and the function can be executed via `.Call`. Needless to say, we *strongly* recommend using a package, or at least Rcpp Attributes as either approach takes care of a lot of these tedious and error-prone manual steps.

2.6 But R CMD SHLIB still does not work !

We have had reports in the past where build failures occurred when users had non-standard code in their `~/.Rprofile` or `Rprofile.site` (or equivalent) files.

If such code emits text on `stdout`, the frequent and implicit invocation of `Rscript -e "..."` (as in FAQ 2.5 above) to retrieve settings directly from **Rcpp** will fail.

You may need to uncomment such non-standard code, or protect it by wrapping it inside `if (interactive())`, or possibly try to use `Rscript -vanilla` instead of plain `Rscript`.

2.7 What about `LinkingTo` ?

R has only limited support for cross-package linkage.

We now employ the `LinkingTo` field of the `DESCRIPTION` file of packages using **Rcpp**. But this only helps in having R compute the location of the header files for us.

The actual library location and argument still needs to be provided by the user. How to do so has been shown above, and we recommned you use either FAQ 2.1 or FAQ 2.2.1 both which use the **Rcpp** function `Rcpp:::LdFlags()`.

If and when `LinkingTo` changes and lives up to its name, we will be sure to adapt **Rcpp** as well.

An important change arrive with **Rcpp** release 0.11.0 and concern the automatic registration of functions; see Section 2.15 below.

2.8 Does Rcpp work on windows ?

Yes of course. See the Windows binaries provided by CRAN.

2.9 Can I use Rcpp with Visual Studio ?

Not a chance.

And that is not because we are meanies but because R and Visual Studio simply do not get along. As **Rcpp** is all about extending R with C++ interfaces, we are bound by the available toolchain. And R simply does not compile with Visual Studio. Go complain to its vendor if you are still upset.

2.10 I am having problems building Rcpp on OS X, any help ?

OS X used to be a little more conservative with compiler versions as Apple stuck with gcc-4.2. Following the 'Mavericks' release, it is the opposite as only llvm is provide in Xcode. Moreover, OS X currently has two incompatible binary modes for the pre- and post-Mavericks world, and CRAN provides releases for both variants. Users have to ensure they are not mixing releases between packages, and packages and the corresponding R binary package.

A helpful post was provided by Brian Ripley in April 2014 on the `r-sig-mac` list, which is generally recommended for OS X-specific questions. and further consultation.

Another helpful write-up for installation / compilation on OS X Mavericks is provided by the BioConductor project.

Also see FAQ 2.16 below.

2.11 Does Rcpp work on solaris/suncc ?

Yes, it generally does. But as we do not have access to such systems, some issues persist on the CRAN test systems.

2.12 Does Rcpp work with Revolution R ?

We have not tested it yet. **Rcpp** might need a few tweaks to work with the compilers used by Revolution R (if those differ from the defaults).

2.13 Is it related to CXXR ?

CXXR is an ambitious project that aims to totally refactor the R interpreter in C++. There are a few similaritites with **Rcpp** but the projects are unrelated.

CXXR and **Rcpp** both want R to make more use of C++ but they do it in very different ways.

2.14 How do I quickly prototype my code using Attributes?

Rcpp version 0.10.0 and later offer a new feature 'Rcpp Attributes' which is described in detail in its own vignette (Allaire et al., 2013). In short, it offers functions `evalCpp`, `cppFunction` and `sourceCpp` which extend the functionality of the `cxxfunction` function.

2.15 What about the new 'no-linking' feature??

Starting with **Rcpp** 0.11.0, functionality provided by **Rcpp** and used by packages built with **Rcpp** accessed via the registration facility offered by R (and which is used by **lme4** and **Matrix**, as well as by **xts** and **zoo**). This requires no effort from the user / programmer, and even frees us from explicit linking instruction. In most cases, the files `src/Makevars` and `src/Makevars.win` can now be removed. Exceptions are the use of **RcppArmadillo** (which needs an entry `PKG_LIBS=$(LAPACK_LIBS) $(BLAS_LIBS) $(FLIBS)`) and packages linking to external libraries they use.

But for most packages using **Rcpp**, only two things are required:

- an entry in `DESCRIPTION` such as `Imports: Rcpp` (which may be versioned as in `Imports: Rcpp (>= 0.11.0)`), and
- an entry in `NAMESPACE` to ensure **Rcpp** is correctly instantiated, for example `importFrom(Rcpp, evalCpp)`.

The name of the symbol does really matter; once one symbol is important all should be available.

2.16 I am having problems building RcppArmadillo on OS X, any help ?

For recent OS X versions Mavericks and beyond, you need to install the additional gfortran 4.8.2 for Darwin 13 package from the `r.research.att.com` site maintained by Simon. See this post for details.

Also see FAQ 2.10 above, and the links provided in that answer.

3 Examples

The following questions were asked on the Rcpp-devel mailing list, which is our preferred place to ask questions as it guarantees exposure to a number of advanced Rcpp users. The StackOverflow tag for rcpp is an alternative; that site is also easily searchable.

Several dozen fully documented examples are provided at the Rcpp Gallery – which is also open for new contributions.

3.1 Can I use templates with Rcpp ?

> *I'm curious whether one can provide a class definition inline in an R script and then initialize an instance of the class and call a method on the class, all inline in R.*

This question was initially about using templates with **inline**, and we show that (older) answer first. It is also easy with Rcpp Attributes which is what we show below.

3.1.1 Using inline

Most certainly, consider this simple example of a templated class which squares its argument:

```
inc <- 'template <typename T>
        class square : public std::unary_function<T,T> {
        public:
            T operator()( T t) const { return t*t ;}
        };
       '

src <- '
       double x = Rcpp::as<double>(xs);
       int i = Rcpp::as<int>(is);
       square<double> sqdbl;
       square<int> sqint;
       return Rcpp::DataFrame::create(Rcpp::Named("x", sqdbl(x)),
                                      Rcpp::Named("i", sqint(i)));
       '
fun <- cxxfunction(signature(xs="numeric", is="integer"),
                   body=src, include=inc, plugin="Rcpp")

fun(2.2, 3L)
```

3.1.2 Using Rcpp Attributes

We can also use 'Rcpp Attributes' (Allaire et al., 2013)—as described in FAQ 2.2.2 and FAQ 2.14 above. Simply place the following code into a file and use `sourceCpp` on it. It will even run the R part at the end.

```
#include <Rcpp.h>

template <typename T> class square : public std::unary_function<T,T> {
public:
    T operator()( T t) const { return t*t ;}
};

// [[Rcpp::export]]
Rcpp::DataFrame fun(double x, int i) {
       square<double> sqdbl;
       square<int> sqint;
       return Rcpp::DataFrame::create(Rcpp::Named("x", sqdbl(x)),
                                      Rcpp::Named("i", sqint(i)));
}

/*** R
fun(2.2, 3L)
*/
```

3.2 Can I do matrix algebra with Rcpp ?

> ***Rcpp** allows element-wise operations on vector and matrices through operator overloading and STL interface, but what if I want to multiply a matrix by a vector, etc ...*

Currently, **Rcpp** does not provide binary operators to allow operations involving entire objects. Adding operators to **Rcpp** would be a major project (if done right) involving advanced techniques such as expression templates. We currently do not plan to go in this direction, but we would welcome external help. Please send us a design document.

However, we have developed the **RcppArmadillo** package (François, Eddelbuettel, and Bates, 2014; Eddelbuettel and Sanderson, 2014) that provides a bridge between **Rcpp** and **Armadillo** (Sanderson, 2010). **Armadillo** supports binary operators on its types in a way that takes full advantage of expression templates to remove temporaries and allow chaining of operations. That is a mouthful of words meaning that it makes the code go faster by using fiendishly clever ways available via the so-called template meta programming, an advanced C++ technique. Also, the **RcppEigen** package (Bates and Eddelbuettel, 2013) provides an alternative using the Eigen template library.

3.2.1 Using inline

The following example is adapted from the examples available at the project page of Armadillo. It calculates $x' \times Y^{-1} \times z$

```
// copy the data to armadillo structures
arma::colvec x = Rcpp::as<arma::colvec> (x_);
arma::mat Y = Rcpp::as<arma::mat>(Y_) ;
arma::colvec z = Rcpp::as<arma::colvec>(z_) ;

// calculate the result
double result = arma::as_scalar(arma::trans(x) * arma::inv(Y) * z);

// return it to R
return Rcpp::wrap( result );
```

If stored in a file `myfile.cpp`, we can use it via **inline**:

```
> fx <- cxxfunction(signature(x_="numeric", Y_="matrix", z_="numeric" ),
+                   paste(readLines("myfile.cpp"), collapse="\n"),
+                   plugin="RcppArmadillo" )
> fx(1:4, diag(4), 1:4)
```

The focus is on the code `arma::trans(x) * arma::inv(Y) * z`, which performs the same operation as the R code `t(x) %*% solve(Y) %*% z`, although Armadillo turns it into only one operation, which makes it quite fast. Armadillo benchmarks against other C++ matrix algebra libraries are provided on the Armadillo website.

It should be noted that code below depends on the version `0.3.5` of **inline** and the version `0.2.2` of **RcppArmadillo**

3.2.2 Using Rcpp Attributes

We can also write the same example for use with Rcpp Attributes:

```
#include <RcppArmadillo.h>

// [[Rcpp::depends(RcppArmadillo)]]

// [[Rcpp::export]]
double fx(arma::colvec x, arma::mat Y, arma::colvec z) {
    // calculate the result
    double result = arma::as_scalar(arma::trans(x) * arma::inv(Y) * z);
    return result;
}

/*** R
fx(1:4, diag(4), 1:4)
*/
```

Here, the additional `Rcpp::depends(RcppArmadillo)` ensures that code can be compiled against the **RcppArmadillo** header, and that the correct libraries are linked to the function built from the supplied code example.

Note how we do not have to concern ourselves with conversion; R object automatically become (Rcpp)Armadillo objects and we can focus on the single computing a (scalar) result.

3.3 Can I use code from the Rmath header and library with Rcpp ?

> *Can I call functions defined in the Rmath header file and the standalone math library for R–as for example the random number generators?*

Yes, of course. This math library exports a subset of R, but **Rcpp** has access to much more. Here is another simple example. Note how we have to use and instance of the `RNGScope` class to set and re-set the random-number generator. This also illustrates Rcpp sugar as we are using a vectorised call to `rnorm`. Moreover, because the RNG is reset, the two calls result in the same random draws. If we wanted to control the draws, we could explicitly set the seed after the `RNGScope` object has been instantiated.

```
> fx <- cxxfunction(signature(),
+                    'RNGScope();
                    return rnorm(5, 0, 100);',
+                    plugin="Rcpp")
> set.seed(42)
> fx()
[1] 137.09584 -56.46982  36.31284  63.28626  40.42683
> fx()
[1] -10.612452 151.152200  -9.465904 201.842371  -6.271410
```

Newer versions of Rcpp also provide the actual Rmath function in the `R` namespace, *i.e.* as `R::rnorm(m,s)` to obtain a scalar random variable distributed as $N(m,s)$.

Using Rcpp Attributes, this can be as simple as

```
> cppFunction('Rcpp::NumericVector ff(int n) { return rnorm(n, 0, 100); }')
> set.seed(42)
> ff(5)
[1] 137.09584 -56.46982  36.31284  63.28626  40.42683
> ff(5)
[1] -10.612452 151.152200  -9.465904 201.842371  -6.271410
> set.seed(42)
> rnorm(5, 0, 100)
[1] 137.09584 -56.46982  36.31284  63.28626  40.42683
> rnorm(5, 0, 100)
[1] -10.612452 151.152200  -9.465904 201.842371  -6.271410
```

This illustrates the Rcpp Attributes adds the required `RNGScope` object for us. It also shows how setting the seed from R affects draws done via C++ as well as R, and that identical random number draws are obtained.

3.4 Can I use NA and Inf with Rcpp ?

R knows about NA and Inf. How do I use them from C++?

Yes, see the following example:

```
> src <- 'Rcpp::NumericVector v(4);
        v[0] = R_NegInf;  // -Inf
        v[1] = NA_REAL;   // NA
        v[2] = R_PosInf;  // Inf
        v[3] = 42;        // see the Hitchhiker Guide
        return Rcpp::wrap(v);'
> fun <- cxxfunction(signature(), src, plugin="Rcpp")
> fun()
[1] -Inf   NA  Inf   42
```

Similarly, for Rcpp Attributes:

```
#include <Rcpp.h>

// [[Rcpp::export]]
Rcpp::NumericVector fun(void) {
    Rcpp::NumericVector v(4);
    v[0] = R_NegInf;  // -Inf
    v[1] = NA_REAL;   // NA
    v[2] = R_PosInf;  // Inf
    v[3] = 42;        // see the Hitchhiker Guide
    return v;
}
```

3.5 Can I easily multiply matrices ?

Can I multiply matrices easily?

Yes, via the **RcppArmadillo** package which builds upon **Rcpp** and the wonderful Armadillo library described above in FAQ 3.2:

```
> txt <- 'arma::mat Am = Rcpp::as< arma::mat >(A);
        arma::mat Bm = Rcpp::as< arma::mat >(B);
        return Rcpp::wrap( Am * Bm );'
> mmult <- cxxfunction(signature(A="numeric", B="numeric"),
+                       body=txt, plugin="RcppArmadillo")
> A <- matrix(1:9, 3, 3)
> B <- matrix(9:1, 3, 3)
> C <- mmult(A, B)
```

Armadillo supports a full range of common linear algebra operations.

The **RcppEigen** package provides an alternative using the Eigen template library.

Rcpp Attributes, once again, makes this even easier:

```
#include <RcppArmadillo.h>

// [[Rcpp::depends(RcppArmadillo)]]

// [[Rcpp::export]]
arma::mat mult(arma::mat A, arma::mat B) {
    return A*B;
}

/*** R
A <- matrix(1:9, 3, 3)
B <- matrix(9:1, 3, 3)
mult(A,B)
*/
```

which can be built, and run, from R via a simple `sourceCpp` call—and will also run the small R example at the end.

3.6 How do I write a plugin for inline and/or Rcpp Attributes?

*How can I create my own plugin for use by the **inline** package?*

Here is an example which shows how to it using GSL libraries as an example. This is merely for demonstration, it is also not perfectly general as we do not detect locations first—but it serves as an example:

```
> gslrng <- '
int seed = Rcpp::as<int>(par) ;
gsl_rng_env_setup();
gsl_rng *r = gsl_rng_alloc (gsl_rng_default);
gsl_rng_set (r, (unsigned long) seed);
double v = gsl_rng_get (r);
gsl_rng_free(r);
return Rcpp::wrap(v);'
> plug <- Rcpp:::Rcpp.plugin.maker(
+     include.before = "#include <gsl/gsl_rng.h>",
+     libs = paste("-L/usr/local/lib/R/site-library/Rcpp/lib -lRcpp",
+                  "-Wl,-rpath,/usr/local/lib/R/site-library/Rcpp/lib",
+                  "-L/usr/lib -lgsl -lgslcblas -lm"))
> registerPlugin("gslDemo", plug )
> fun <- cxxfunction(signature(par="numeric"), gslrng, plugin="gslDemo")
> fun(0)
```

Here the **Rcpp** function `Rcpp.plugin.maker` is used to create a plugin 'plug' which is then registered, and subsequently used by **inline**.

The same plugins can be used by Rcpp Attributes as well.

3.7 How can I pass one additional flag to the compiler?

How can I pass another flag to the g++ compiler without writing a new plugin?

The quickest way is to modify the return value from an existing plugin. Here we use the default one from **Rcpp** itself in order to pass the new flag `-std=c++0x`. As it does not set the `PKG_CXXFLAGS` variable, we simply assign this. For other plugins, one may need to append to the existing values instead.

```
> myplugin <- getPlugin("Rcpp")
> myplugin$env$PKG_CXXFLAGS <- "-std=c++11"
> f <- cxxfunction(signature(), settings=myplugin, body='
+    std::vector<double> x = { 1.0, 2.0, 3.0 };  // fails without -std=c++0x
+    return Rcpp::wrap(x);
+ ')
> f()
```

For Rcpp Attributes, the attributes `Rcpp::plugin()` can be used. Currently supported plugins are for C++11 as well as for OpenMP.

3.8 How can I set matrix row and column names ?

Ok, I can create a matrix, but how do I set its row and columns names?

Pretty much the same way as in R itself: We define a list with two character vectors, one each for row and column names, and assign this to the `dimnames` attribute:

```
> src <- '
  Rcpp::NumericMatrix x(2,2);
  x.fill(42);                          // or more interesting values
  Rcpp::List dimnms =                  // two vec. with static names
      Rcpp::List::create(Rcpp::CharacterVector::create("cc", "dd"),
                         Rcpp::CharacterVector::create("ee", "ff"));
  // and assign it
  x.attr("dimnames") = dimnms;
  return(x);
'
> fun <- cxxfunction(signature(), body=src, plugin="Rcpp")
> fun()
```

The same logic, but used with Rcpp Attributes:

```
#include <Rcpp.h>

// [[Rcpp::export]]
Rcpp::List fun(void) {
    Rcpp::NumericMatrix x(2,2);
    x.fill(42);                          // or more interesting values
    Rcpp::List dimnms =                  // two vec. with static names
        Rcpp::List::create(Rcpp::CharacterVector::create("cc", "dd"),
                           Rcpp::CharacterVector::create("ee", "ff"));
    // and assign it
    x.attr("dimnames") = dimnms;
    return(x);
}
```

3.9 Why can long long types not be cast correctly?

That is a good and open question. We rely on the basic R types, notably `integer` and `numeric`. These can be cast to and from C++ types without problems. But there are corner cases. The following example, contributed by a user, shows that we cannot reliably cast `long` types (on a 64-bit machines).

```
> BigInts <- cxxfunction(signature(),
+   'std::vector<long> bigints;
   bigints.push_back(12345678901234567LL);
   bigints.push_back(12345678901234568LL);
   Rprintf("Difference of %ld\\n", 12345678901234568LL - 12345678901234567LL);
   return wrap(bigints);', plugin="Rcpp", includes="#include <vector>")
> retval<-BigInts()
> stopifnot(length(unique(retval)) == 2)
```

While the difference of one is evident at the C++ level, it is no longer present once cast to R. The 64-bit integer values get cast to a floating point types with a 53-bit mantissa. We do not have a good suggestion or fix for casting 64-bit integer values: 32-bit integer values fit into `integer` types, up to 53 bit precision fits into `numeric` and beyond that truly large integers may have to converted (rather crudely) to text and re-parsed. Using a different representation as for example from the GNU Multiple Precision Arithmetic Library may be an alternative.

4 Support

4.1 Is the API documented ?

You bet. We use doxygen to generate html, latex and man page documentation from the source. The html documentation is available for browsing, as a very large pdf file, and all three formats are also available a zip-archives: html, latex, and man.

4.2 Does it really work ?

We take quality seriously and have developped an extensive unit test suite to cover many possible uses of the **Rcpp** API.

We are always on the look for more coverage in our testing. Please let us know if something has not been tested enough.

4.3 Where can I ask further questions ?

The Rcpp-devel mailing list hosted at R-forge is by far the best place. You may also want to look at the list archives to see if your question has been asked before.

You can also use Stack Overflow via its 'rcpp' tag.

4.4 Where can I read old questions and answers ?

The normal Rcpp-devel mailing list hosting at R-forge contains an archive, which can be searched via swish.

Alternatively, one can also use Gmane on Rcpp-devel as well as Mail-Archive on Rcpp-devel both of which offer web-based interfaces, including searching.

4.5 I like it. How can I help ?

We maintain a list of open issues in the Github repository. We welcome pull requests and suggest that code submissions come corresponding unit tests and, if applicable, documentation.

If you are willing to donate time and have skills in C++, let us know. If you are willing to donate money to sponsor improvements, let us know too.

You can also spread the word about **Rcpp**. There are many packages on CRAN that use C++, yet are not using **Rcpp**. You could blog about it, or get the word out otherwise.

Last but not least the Rcpp Gallery is open for user contributions.

4.6 I don't like it. How can I help ?

It is very generous of you to still want to help. Perhaps you can tell us what it is that you dislike. We are very open to *constructive* criticism.

4.7 Can I have commercial support for Rcpp ?

Sure you can. Just send us an email, and we will be happy to discuss the request.

4.8 I want to learn quickly. Do you provide training courses ?

Yes. Just send us an email.

4.9 Where is the code repository ?

From late 2008 to late 2013, we used the Subversion repository at R-Forge which contained Rcpp and a number of related packages. It still has the full history as well as number of support files.

We have since switched to a Git repository at Github for Rcpp (as well as RcppArmadillo and RcppEigen).

References

J. J. Allaire, Dirk Eddelbuettel, and Romain François. *Rcpp Attributes*, 2013. URL http://CRAN.R-Project.org/package=Rcpp. Vignette included in R package Rcpp.

Douglas Bates and Dirk Eddelbuettel. Fast and elegant numerical linear algebra using the RcppEigen package. *Journal of Statistical Software*, 52(5):1–24, 2013. URL http://www.jstatsoft.org/v52/i05/.

Dirk Eddelbuettel. *Seamless R and C++ Integration with Rcpp*. Use R! Springer, New York, 2013. ISBN 978-1-4614-6867-7.

Dirk Eddelbuettel and Romain François. Rcpp: Seamless R and C++ integration. *Journal of Statistical Software*, 40(8): 1–18, 2011. URL http://www.jstatsoft.org/v40/i08/.

Dirk Eddelbuettel and Conrad Sanderson. RcppArmadillo: Accelerating R with high-performance C++ linear algebra. *Computational Statistics and Data Analysis*, 71:1054–1063, March 2014. doi: 10.1016/j.csda.2013.02.005. URL http://dx.doi.org/10.1016/j.csda.2013.02.005.

Dirk Eddelbuettel, Romain François, JJ Allaire, John Chambers, Douglas Bates, and Kevin Ushey. *Rcpp: Seamless R and C++ Integration*, 2014. URL http://CRAN.R-Project.org/package=Rcpp. R package version 0.11.0.

Romain François, Dirk Eddelbuettel, and Douglas Bates. *RcppArmadillo: Rcpp integration for Armadillo templated linear algebra library*, 2014. URL http://CRAN.R-Project.org/package=RcppArmadillo. R package version 0.4.000.2.

R Development Core Team. *Writing R extensions*. R Foundation for Statistical Computing, Vienna, Austria, 2012. URL http://CRAN.R-Project.org/doc/manuals/R-exts.html.

R Development Core Team. *R Installation and Administration*. R Foundation for Statistical Computing, Vienna, Austria, 2013. URL http://CRAN.R-Project.org/doc/manuals/R-admin.html. ISBN 3-900051-09-7.

Conrad Sanderson. Armadillo: An open source C++ algebra library for fast prototyping and computationally intensive experiments. Technical report, NICTA, 2010. URL http://arma.sf.net.

Oleg Sklyar, Duncan Murdoch, Mike Smith, Dirk Eddelbuettel, and Romain François. *inline: Inline C, C++, Fortran function calls from R*, 2013. URL http://CRAN.R-Project.org/package=inline. R package version 0.3.13.
