# Supplementary material for: The relationship between leaf area growth and biomass accumulation in Arabidopsis thaliana
Source: Front Plant Sci. 2015 Apr 9;6:167. doi: 10.3389/fpls.2015.00167 (PMC4391269; doi:10.3389/fpls.2015.00167)
Supplement: Supplementary file 4 [file DataSheet1.ZIP › R_libs - parallization.zip!/R_libs/Rcpp/doc/Rcpp-package.pdf]

Writing a package that uses **Rcpp**

Dirk Eddelbuettel Romain François

Rcpp version 0.11.3 as of September 27, 2014

Abstract

This document provides a short overview of how to use **Rcpp** (Eddelbuettel, François, Allaire, Chambers, Bates, and Ushey, 2014; Eddelbuettel and François, 2011; Eddelbuettel, 2013) when writing an R package. It shows how usage of the function `Rcpp.package.skeleton` which creates a complete and self-sufficient example package using **Rcpp**. All components of the directory tree created by `Rcpp.package.skeleton` are discussed in detail. This document thereby complements the *Writing R Extensions* manual (R Development Core Team, 2012) which is the authoritative source on how to extend R in general.

1 Introduction

Rcpp (Eddelbuettel et al., 2014; Eddelbuettel and François, 2011; Eddelbuettel, 2013) is an extension package for R which offers an easy-to-use yet featureful interface between C++ and R. However, it is somewhat different from a traditional R package because its key component is a C++ library. A client package that wants to make use of the **Rcpp** features must link against the library provided by **Rcpp**.

It should be noted that R has only limited support for C(++)-level dependencies between packages (R Development Core Team, 2012). The `LinkingTo` declaration in the package `DESCRIPTION` file allows the client package to retrieve the headers of the target package (here **Rcpp**), but support for linking against a library is not provided by R and has to be added manually.

This document follows the steps of the `Rcpp.package.skeleton` function to illustrate a recommended way of using **Rcpp** from a client package. We illustrate this using a simple C++ function which will be called by an R function.

We strongly encourage the reader to become familiar with the material in the *Writing R Extensions* manual (R Development Core Team, 2012), as well as with other documents on R package creation such as Leisch (2008). Given a basic understanding of how to create R package, the present document aims to provide the additional information on how to use **Rcpp** in such add-on packages.

2 Using `Rcpp.package.skeleton`

2.1 Overview

Rcpp provides a function `Rcpp.package.skeleton`, modeled after the base R function `package.skeleton`, which facilitates creation of a skeleton package using **Rcpp**.

`Rcpp.package.skeleton` has a number of arguments documented on its help page (and similar to those of `package.skeleton`). The main argument is the first one which provides the name of the package one aims to create by invoking the function. An illustration of a call using an argument `mypackage` is provided below.

```
> Rcpp.package.skeleton("mypackage")
```

```
$ ls -1R mypackage/
DESCRIPTION
NAMESPACE
R
Read-and-delete-me
man
src

mypackage/R:
RcppExports.R

mypackage/man:
mypackage-package.Rd
rcpp_hello_world.Rd

mypackage/src:
Makevars            ## up until Rcpp 0.10.6, see below
Makevars.win        ## up until Rcpp 0.10.6, see below
RcppExports.cpp
rcpp_hello_world.cpp
$
```

Using `Rcpp.package.skeleton` is by far the simplest approach as it fulfills two roles. It creates the complete set of files needed for a package, and it also includes the different components needed for using **Rcpp** that we discuss in the following sections.

2.2 C++ code

If the `attributes` argument is set to `TRUE`[1], the following C++ file is included in the `src/` directory:

```cpp
#include <Rcpp.h>
using namespace Rcpp;

// [[Rcpp::export]]
List rcpp_hello_world() {

    CharacterVector x = CharacterVector::create( "foo", "bar" )  ;
    NumericVector y   = NumericVector::create( 0.0, 1.0 ) ;
    List z            = List::create( x, y ) ;

    return z ;
}
```

The file defines the simple `rcpp_hello_world` function that uses a few **Rcpp** classes and returns a `List`.

This function is preceded by the `Rcpp::export` attribute to automatically handle argument conversion because R has to be taught how to e.g. handle the `List` class.

`Rcpp.package.skeleton` then invokes `compileAttributes` on the package, which generates the `RcppExports.cpp` file:

[1] Setting `attributes` to `TRUE` is the default. This document does not cover the behavior of `Rcpp.package.skeleton` when `attributes` is set to `FALSE` as we try to encourage package developpers to use attributes.

```
// This file was generated by Rcpp::compileAttributes
// Generator token: 10BE3573-1514-4C36-9D1C-5A225CD40393

#include <Rcpp.h>

using namespace Rcpp;

// rcpp_hello_world
List rcpp_hello_world();
RcppExport SEXP mypackage_rcpp_hello_world() {
BEGIN_RCPP
    SEXP __sexp_result;
    {
        Rcpp::RNGScope __rngScope;
        List __result = rcpp_hello_world();
        PROTECT(__sexp_result = Rcpp::wrap(__result));
    }
    UNPROTECT(1);
    return __sexp_result;
END_RCPP
}
```

This file defines a function with the appropriate calling convention, suitable for `.Call`. It needs to be regenerated each time functions exposed by attributes are modified. This is the task of the `compileAttributes` function. A discussion on attributes is beyond the scope of this document and more information is available in the attributes vignette (Allaire, Eddelbuettel, and François, 2013).

2.3 R code

The `compileAttributes` also generates R code that uses the C++ function.

```
# This file was generated by Rcpp::compileAttributes
# Generator token: 10BE3573-1514-4C36-9D1C-5A225CD40393

rcpp_hello_world <- function() {
    .Call('mypackage_rcpp_hello_world', PACKAGE = 'mypackage')
}
```

This is also a generated file so it should not be modified manually, rather regenerated as needed by `compileAttributes`.

2.4 `DESCRIPTION`

The skeleton generates an appropriate `DESCRIPTION` file, using both `Imports:` and `LinkingTo` for **Rcpp**:

```
Package: mypackage
Type: Package
Title: What the package does (short line)
Version: 1.0
Date: 2013-09-17
Author: Who wrote it
Maintainer: Who to complain to <yourfault@somewhere.net>
Description: More about what it does (maybe more than one line)
License: What Licence is it under ?
Imports: Rcpp (>= 0.11.0)
LinkingTo: Rcpp
```

`Rcpp.package.skeleton` adds the three last lines to the `DESCRIPTION` file generated by `package.skeleton`.

The `Imports` declaration indicates R-level dependency between the client package and **Rcpp**; code from the latter is being imported into the package described here. The `LinkingTo` declaration indicates that the client package needs to use header files exposed by **Rcpp**.

2.5 Now optional: `Makevars` and `Makevars.win`

This behaviour changed with **Rcpp** release 0.11.0. These files used to be mandatory, now they are merely optional.

We will describe the old setting first as it was in use for a few years. The new standard, however, is much easier and is described below.

2.5.1 Releases up until 0.10.6

Unfortunately, the `LinkingTo` declaration in itself was not enough to link to the user C++ library of **Rcpp**. Until more explicit support for libraries is added to R, ones needes to manually add the **Rcpp** library to the `PKG_LIBS` variable in the `Makevars` and `Makevars.win` files. (This has now changed with release 0.11.0; see below). **Rcpp** provides the unexported function `Rcpp:::LdFlags()` to ease the process:

```
## Use the R_HOME indirection to support installations of multiple R version
PKG_LIBS = `$(R_HOME)/bin/Rscript -e "Rcpp:::LdFlags()"`

## As an alternative, one can also add this code in a file 'configure'
##
##    PKG_LIBS=`${R_HOME}/bin/Rscript -e "Rcpp:::LdFlags()"`
##
##    sed -e "s|@PKG_LIBS@|${PKG_LIBS}|" \
##        src/Makevars.in > src/Makevars
##
## which together with the following file 'src/Makevars.in'
##
##    PKG_LIBS = @PKG_LIBS@
##
## can be used to create src/Makevars dynamically. This scheme is more
## powerful and can be expanded to also check for and link with other
## libraries.  It should be complemented by a file 'cleanup'
##
##    rm src/Makevars
##
## which removes the autogenerated file src/Makevars.
##
## Of course, autoconf can also be used to write configure files. This is
## done by a number of packages, but recommended only for more advanced users
## comfortable with autoconf and its related tools.
```

The `Makevars.win` is the equivalent, targeting windows.

```
## Use the R_HOME indirection to support installations of multiple R version
PKG_LIBS = $(shell "${R_HOME}/bin${R_ARCH_BIN}/Rscript.exe" -e "Rcpp:::LdFlags()")
```

2.5.2 Releases since 0.11.0

As of release 0.11.0, this is no longer needed as client packages obtain the required code from **Rcpp** via explicit function registration. The user does not have to do anything.

This means that `PKG_LIBS` can now be empty—unless some client libraries are needed. For example, **RcppCNPy** needs compression support and hence uses `PKG_LIBS= -lz`. Similarly, when a third-party library is required, it can and should be set here.

2.6 `NAMESPACE`

The `Rcpp.package.skeleton` function also creates a file `NAMESPACE`.

```
useDynLib(mypackage)
exportPattern("^[[:alpha:]]+")
importFrom(Rcpp, evalCpp)
```

This file serves three purposes. First, it ensure that the dynamic library contained in the package we are creating via `Rcpp.package.skeleton` will be loaded and thereby made available to the newly created R package.

Second, it declares which functions should be globally visible from the namespace of this package. As a reasonable default, we export all functions.

Third, it instructs R to import a symbol from **Rcpp**. This sets up the import of all registered function and, together with the `Imports:` statement in `DESCRIPTION`, provides what is needed for client packages to access **Rcpp** functionality.

2.7 Help files

Also created is a directory `man` containing two help files. One is for the package itself, the other for the (single) R function being provided and exported.

The *Writing R Extensions* manual (R Development Core Team, 2012) provides the complete documentation on how to create suitable content for help files.

2.7.1 `mypackage-package.Rd`

The help file `mypackage-package.Rd` can be used to describe the new package.

```
\name{mypackage-package}
\alias{mypackage-package}
\alias{mypackage}
\docType{package}
\title{
What the package does (short line)
}
\description{
More about what it does (maybe more than one line)
~~ A concise (1-5 lines) description of the package ~~
}
\details{
\tabular{ll}{
Package: \tab mypackage\cr
Type: \tab Package\cr
Version: \tab 1.0\cr
Date: \tab 2013-09-17\cr
License: \tab What license is it under?\cr
}
~~ An overview of how to use the package, including the most important functions ~~
}
\author{
Who wrote it

Maintainer: Who to complain to <yourfault@somewhere.net>
}
\references{
~~ Literature or other references for background information ~~
}
~~ Optionally other standard keywords, one per line, from file KEYWORDS in the R documentation directory ~~
\keyword{ package }
\seealso{
~~ Optional links to other man pages, e.g. ~~
~~ \code{\link[<pkg>:<pkg>-package]{<pkg>}} ~~
}
\examples{
%% ~~ simple examples of the most important functions ~~
}
```

2.7.2 rcpp_hello_world.Rd

The help file rcpp_hello_world.Rd serves as documentation for the example R function.

```
\name{rcpp_hello_world}
\alias{rcpp_hello_world}
\docType{package}
\title{
Simple function using Rcpp
}
\description{
Simple function using Rcpp
}
\usage{
rcpp_hello_world()
}
\examples{
\dontrun{
rcpp_hello_world()
}
}
```

3 Using modules

This document does not cover the use of the `module` argument of `Rcpp.package.skeleton`. It is covered in the modules vignette (Eddelbuettel and François, 2014).

4 Further examples

The canonical example of a package that uses **Rcpp** is the **RcppExamples** (Eddelbuettel and François, 2013) package. **RcppExamples** contains various examples of using **Rcpp**. Hence, the **RcppExamples** package is provided as a template for employing **Rcpp** in packages.

Other CRAN packages using the **Rcpp** package are **RcppArmadillo** (François, Eddelbuettel, and Bates, 2014), and **minqa** (Bates, Mullen, Nash, and Varadhan, 2012). Several other packages follow older (but still supported and appropriate) instructions. They can serve examples on how to get data to and from C++ routines, but should not be considered templates for how to connect to **Rcpp**. The full list of packages using **Rcpp** can be found at the CRAN page of **Rcpp**.

5 Other compilers

Less experienced R users on the Windows platform frequently ask about using **Rcpp** with the Visual Studio toolchain. That is simply not possible as R is built with the **gcc** compiler. Different compilers have different linking conventions. These conventions are particularly hairy when it comes to using C++. In short, it is not possible to simply drop sources (or header files) from **Rcpp** into a C++ project built with Visual Studio, and this note makes no attempt at claiming otherwise.

Rcpp is fully usable on Windows provided the standard Windows toolchain for R is used. See the *Writing R Extensions* manual (R Development Core Team, 2012) for details.

6 Summary

This document described how to use the **Rcpp** package for R and C++ integration when writing an R extension package. The use of the `Rcpp.package.skeleton` was shown in detail, and references to further examples were provided.

References

J. J. Allaire, Dirk Eddelbuettel, and Romain François. *Rcpp Attributes*, 2013. URL http://CRAN.R-Project.org/package=Rcpp. Vignette included in R package Rcpp.

Douglas Bates, Katharine M. Mullen, John C. Nash, and Ravi Varadhan. *minqa: Derivative-free optimization algorithms by quadratic approximation*, 2012. URL `http://CRAN.R-Project.org/package=minqa`. R package version 1.2.2.

Dirk Eddelbuettel. *Seamless R and C++ Integration with Rcpp*. Use R! Springer, New York, 2013. ISBN 978-1-4614-6867-7.

Dirk Eddelbuettel and Romain François. Rcpp: Seamless R and C++ integration. *Journal of Statistical Software*, 40(8): 1–18, 2011. URL `http://www.jstatsoft.org/v40/i08/`.

Dirk Eddelbuettel and Romain François. *RcppExamples: Examples using Rcpp to interface R and C++*, 2013. URL `http://CRAN.R-Project.org/package=RcppExamples`. R package version 0.1.6.

Dirk Eddelbuettel and Romain François. *Exposing C++ functions and classes with Rcpp modules*, 2014. URL `http://CRAN.R-Project.org/package=Rcpp`. Vignette included in R package Rcpp.

Dirk Eddelbuettel, Romain François, JJ Allaire, John Chambers, Douglas Bates, and Kevin Ushey. *Rcpp: Seamless R and C++ Integration*, 2014. URL `http://CRAN.R-Project.org/package=Rcpp`. R package version 0.11.0.

Romain François, Dirk Eddelbuettel, and Douglas Bates. *RcppArmadillo: Rcpp integration for Armadillo templated linear algebra library*, 2014. URL `http://CRAN.R-Project.org/package=RcppArmadillo`. R package version 0.4.000.2.

Friedrich Leisch. Tutorial on Creating R Packages. In Paula Brito, editor, *COMPSTAT 2008 – Proceedings in Computational Statistics*, Heidelberg, 2008. Physica Verlag. URL `http://CRAN.R-Project.org/doc/contrib/Leisch-CreatingPackages.pdf`.

R Development Core Team. *Writing R extensions*. R Foundation for Statistical Computing, Vienna, Austria, 2012. URL `http://CRAN.R-Project.org/doc/manuals/R-exts.html`.
