# Supplementary material for: The relationship between leaf area growth and biomass accumulation in Arabidopsis thaliana
Source: Front Plant Sci. 2015 Apr 9;6:167. doi: 10.3389/fpls.2015.00167 (PMC4391269; doi:10.3389/fpls.2015.00167)
Supplement: Supplementary file 4 [file DataSheet1.ZIP › R_libs - parallization.zip!/R_libs/Rcpp/doc/Rcpp-quickref.pdf]

Rcpp Quick Reference Guide

Romain François　　Dirk Eddelbuettel

Rcpp version 0.11.3 as of September 27, 2014

Important Notes

```
// If you experience compiler errors, please check that you
have an appropriate version of g++. See 'Rcpp-FAQ' for more
information.

// Many of the examples here imply the following:
using namespace Rcpp;
// The inline package adds this for you. Alternately, use e.g.:
Rcpp::NumericVector xx(10);
```

Create simple vectors

```
SEXP x; std::vector<double> y(10);

// from SEXP
NumericVector xx(x);

// of a given size (filled with 0)
NumericVector xx(10);
// ... with a default for all values
NumericVector xx(10, 2.0);

// range constructor
NumericVector xx( y.begin(), y.end() );

// using create
NumericVector xx = NumericVector::create(
    1.0, 2.0, 3.0, 4.0 );
NumericVector yy = NumericVector::create(
    Named["foo"] = 1.0,
    _["bar"]     = 2.0 );   // short for Named
```

Extract and set single elements

```
// extract single values
double x0 = xx[0];
double x1 = xx(1);

double y0 = yy["foo"];
double y1 = yy["bar"];

// set single values
xx[0] = 2.1;
xx(1) = 4.2;

yy["foo"] = 3.0;

// grow the vector
yy["foobar"] = 10.0;
```

Using matrices

```
// Initializing from SEXP,
// dimensions handled automatically
SEXP x;
NumericMatrix xx(x);

// Matrix of 4 rows & 5 columns (filled with 0)
NumericMatrix xx(4, 5);

// Fill with value
int xsize = xx.nrow() * xx.ncol();
for (int i = 0; i < xsize; i++) {
    xx[i] = 7;
}
// Same as above, using STL fill
std::fill(xx.begin(), xx.end(), 8);

// Assign this value to single element
// (1st row, 2nd col)
xx(0,1) = 4;

// Reference the second column
// Changes propagate to xx (same applies for Row)
NumericMatrix::Column zzcol = xx( _, 1);
zzcol = zzcol * 2;

// Copy the second column into new object
NumericVector zz1 = xx( _, 1);
// Copy the submatrix (top left 3x3) into new object
NumericMatrix zz2 = xx( Range(0,2),
Range(0,2));
```

Inline

```
## Note - this is R code. inline allows rapid
testing.
require(inline)
testfun = cxxfunction(
            signature(x="numeric",
i="integer"),
            body = '
                NumericVector xx(x);
                int ii = as<int>(i);
                xx = xx * ii;
                return( xx );
            ', plugin="Rcpp")
testfun(1:5, 3)
```

Interface with R

```
## In R, create a package shell. For details,
see the "Writing R Extensions" manual.

Rcpp.package.skeleton("myPackage")

## Add R code to pkg R/ directory. Call C++
function. Do type-checking in R.

myfunR = function(Rx, Ry) {
    ret = .Call("myCfun", Rx, Ry,
            package="myPackage")
    return(ret)
}

// Add C++ code to pkg src/ directory.
using namespace Rcpp;
// Define function as extern with RcppExport
RcppExport SEXP myCfun( SEXP x, SEXP y) {
    // If R/C++ types match, use pointer to x.   Pointer is
faster, but changes to xx propagate to R ( xx -> x == Rx).
    NumericVector xx(x);
    // clone is slower and uses extra memory. Safe, R-like.
    NumericVector yy(clone(y));
    xx[0] = yy[0] = -1.5;
    int zz = xx[0];
    // use wrap() to return non-SEXP objects, e.g:
    // return(wrap(zz));
    // Build and return a list
    List ret; ret["x"] = xx; ret["y"] = yy;
    return(ret);
}

## From shell, above package directory
R CMD check myPackage  ## Optional
R CMD INSTALL myPackage

## In R:
require(myPackage)
aa = 1.5; bb = 1.5; cc =  myfunR(aa, bb)
aa == bb ## FALSE, C++ modifies aa
aa = 1:2; bb = 1:2; cc =  myfunR(aa, bb)
identical(aa, bb)
## TRUE, R/C++ types don't match
```

STL interface

```
// sum a vector from beginning to end
double s = std::accumulate(x.begin(),
   x.end(), 0.0);
// prod of elements from beginning to end
int p = std::accumulate(vec.begin(),
    vec.end(), 1, std::multiplies<int>());
// inner_product to compute sum of squares
double s2 = std::inner_product(res.begin(),
    res.end(), res.begin(), 0.0);
```

Function

```
Function rnorm("rnorm");
rnorm(100, _["mean"] = 10.2, _["sd"] = 3.2 );
```

Environment

```
Environment stats("package:stats");
Environment env( 2 ); // by position

// special environments
Environment::Rcpp_namespace();
Environment::base_env();
Environment::base_namespace();
Environment::global_env();
Environment::empty_env();

Function rnorm = stats["rnorm"];
glob["x"] = "foo";
glob["y"] = 3;
std::string x = glob["x"];

glob.assign( "foo" , 3 );
int foo = glob.get( "foo" );
int foo = glob.find( "foo" );
CharacterVector names = glob.ls()
bool b = glob.exists( "foo" );
glob.remove( "foo" );

glob.lockBinding("foo");
glob.unlockBinding("foo");
bool b = glob.bindingIsLocked("foo");
bool b = glob.bindingIsActive("foo");

Environment e = stats.parent();
Environment e = glob.new_child();
```

Modules

```
// Warning -- At present, module-based objects do not persist across quit(save="yes")/reload cycles. To be safe, save results to R objects and remove module objects before exiting R.

// To create a module-containing package from R, use:
Rcpp.package.skeleton("mypackage",module=TRUE)
// You will need to edit the RcppModules: line of the DESCRIPTION file to match your module name (in this example, from yada to mod_bar).

class Bar {
  public:
    Bar(double x_) :
      x(x_), nread(0), nwrite(0) {}

    double get_x( ) {
      nread++;    return x;
    }

    void set_x( double x_) {
      nwrite++;   x = x_;
    }

    IntegerVector stats() const {
      return IntegerVector::create(
        _["read"] = nread,
        _["write"] = nwrite);
    }
  private:
    double x; int nread, nwrite;
};

RCPP_MODULE(mod_bar) {
  class_<Bar>( "Bar" )
  .constructor<double>()
  .property( "x", &Bar::get_x, &Bar::set_x,
    "Docstring for x" )
  .method( "stats", &Bar::stats,
    "Docstring for stats")
;}

## The following is R code.
require(mypackage); show(Bar)
b <- new(Bar, 10);  b$x <- 10
b_persist <- list(stats=b$stats(), x=b$x)
rm(b)
```

Rcpp sugar

```
NumericVector x = NumericVector::create(
  -2.0, -1.0, 0.0, 1.0, 2.0 );
IntegerVector y = IntegerVector::create(
  -2, -1, 0, 1, 2 );

NumericVector xx = abs( x );
IntegerVector yy = abs( y );

bool b = all( x < 3.0 ).is_true() ;
bool b = any( y > 2 ).is_true();

NumericVector xx = ceil( x );
NumericVector xx = ceiling( x );
NumericVector yy = floor( y );
NumericVector yy = floor( y );

NumericVector xx = exp( x );
NumericVector yy = exp( y );

NumericVector xx = head( x, 2 );
IntegerVector yy = head( y, 2 );

IntegerVector xx = seq_len( 10 );
IntegerVector yy = seq_along( y );

NumericVector xx = rep( x, 3 );
NumericVector xx = rep_len( x, 10 );
NumericVector xx = rep_each( x, 3 );

IntegerVector yy = rev( y );
```

Random functions

```
// Set seed
RNGScope scope;

// For details see Section 6.7.1--Distribution functions of the 'Writing R Extensions' manual. In some cases (e.g. rnorm), distribution-specific arguments can be omitted; when in doubt, specify all dist-specific arguments. The use of doubles rather than integers for dist-specific arguments is recommended. Unless explicitly specified, log=FALSE.

// Equivalent to R calls
NumericVector xx = runif(20);
NumericVector xx1 = rnorm(20);
NumericVector xx1 = rnorm(20, 0);
NumericVector xx1 = rnorm(20, 0, 1);

// Example vector of quantiles
NumericVector quants(5);
for (int i = 0; i < 5; i++) {
    quants[i] = (i-2);
}

// in R, dnorm(-2:2)
NumericVector yy = dnorm(quants) ;
NumericVector yy = dnorm(quants, 0.0, 1.0) ;

// in R, dnorm(-2:2, mean=2, log=TRUE)
NumericVector yy = dnorm(quants, 2.0, true) ;

// Note - cannot specify sd without mean
// in R, dnorm(-2:2, mean=0, sd=2, log=TRUE)
NumericVector yy = dnorm(quants, 0.0, 2.0,
true) ;

// To get original R api, use Rf_*
double zz = Rf_rnorm(0, 2);
```
