# Supplementary material for: The relationship between leaf area growth and biomass accumulation in Arabidopsis thaliana
Source: Front Plant Sci. 2015 Apr 9;6:167. doi: 10.3389/fpls.2015.00167 (PMC4391269; doi:10.3389/fpls.2015.00167)
Supplement: Supplementary file 4 [file DataSheet1.ZIP › R_libs - parallization.zip!/R_libs/Rcpp/doc/Rcpp-unitTests.pdf]

Rcpp: Unit testing results

Dirk Eddelbuettel Romain François

Rcpp version 0.11.3 as of September 27, 2014

Test Execution

```
The environment variable 'RunAllRcppTests' was not set to 'yes', so skipping some tests.
```

Test Results

```
RUNIT TEST PROTOCOL -- Sat Sep 27 13:09:30 2014
***********************************************
Number of test functions: 0
Number of errors: 0
Number of failures: 0


1 Test Suite :
Rcpp unit testing - 0 test functions, 0 errors, 0 failures



Details
***************************
Test Suite: Rcpp unit testing
Test function regexp: ^test.+
Test file regexp: ^runit.+\.[rR]$
Involved directory:
/tmp/RtmpQUYA94/Rinst52cb8be567/Rcpp/unitTests
```
