# Supplementary material for: The relationship between leaf area growth and biomass accumulation in Arabidopsis thaliana
Source: Front Plant Sci. 2015 Apr 9;6:167. doi: 10.3389/fpls.2015.00167 (PMC4391269; doi:10.3389/fpls.2015.00167)
Supplement: Supplementary file 4 [file DataSheet1.ZIP › R_libs - parallization.zip!/R_libs/Rcpp/html/00Index.html]

R: Seamless R and C++ Integration

# Seamless R and C++ Integration

---

## Documentation for package ‘Rcpp’ version 0.11.3

- DESCRIPTION file.
- User guides, package vignettes and other documentation.
- Package NEWS.

## Help Pages

|  |  |
| --- | --- |
| Rcpp-package | R / C++ interface |
| $-method | Reflection information for an internal c++ class |
| $-method | c++ internal objects |
| $-method | Rcpp modules |
| $<--method | c++ internal objects |
| .DollarNames-method | completion |
| .DollarNames-methods | completion |
| areMacrosDefined | Evaluate a C++ Expression |
| C++Class-class | Reflection information for an internal c++ class |
| C++Constructor-class | Class "C++Constructor" |
| C++Field-class | Class "C++Field" |
| C++Function-class | Class "C++Function" |
| C++Object-class | c++ internal objects |
| C++OverloadedMethods-class | Class "C++OverloadedMethods" |
| compileAttributes | Compile Rcpp Attributes for a Package |
| cppFunction | Define an R Function with a C++ Implementation |
| demangle | c++ type information |
| dependsAttribute | Rcpp::depends Attribute |
| evalCpp | Evaluate a C++ Expression |
| exportAttribute | Rcpp::export Attribute |
| exposeClass | Create an Rcpp Module to Expose a C++ Class in R |
| formals<--method | Set the formal arguments of a C++ function |
| formals<--methods | Set the formal arguments of a C++ function |
| initialize-method | Rcpp modules |
| interfacesAttribute | Rcpp::interfaces Attribute |
| LdFlags | (Deprecated) Rcpp Linker Flags |
| loadModule | Load an Rcpp Module into a Package |
| loadRcppClass | Create a Class Extending a C++ Class |
| loadRcppModules | Loads Rcpp modules on package startup |
| Module | Retrieves an Rcpp module |
| Module-class | Rcpp modules |
| pluginsAttribute | Rcpp::plugins Attribute |
| populate | Populates a namespace or an environment with the content of a module |
| print.bytes | c++ type information |
| prompt-method | Rcpp modules |
| Rcpp | R / C++ interface |
| Rcpp.package.skeleton | Create a skeleton for a new package depending on Rcpp |
| Rcpp.plugin.maker | Facilitating making package plugins |
| RcppClass-class | Create a Class Extending a C++ Class |
| RcppLdFlags | (Deprecated) Rcpp Linker Flags |
| RcppUnitTests | Rcpp : unit tests results |
| registerPlugin | Register an inline plugin |
| setRcppClass | Create a Class Extending a C++ Class |
| show-method | Reflection information for an internal c++ class |
| show-method | Class "C++Function" |
| show-method | c++ internal objects |
| show-method | Rcpp modules |
| sizeof | c++ type information |
| sourceCpp | Source C++ Code from a File or String |
