# Supplementary material for: The relationship between leaf area growth and biomass accumulation in Arabidopsis thaliana
Source: Front Plant Sci. 2015 Apr 9;6:167. doi: 10.3389/fpls.2015.00167 (PMC4391269; doi:10.3389/fpls.2015.00167)
Supplement: Supplementary file 4 [file DataSheet1.ZIP › R_libs - parallization.zip!/R_libs/RcppEigen/doc/RcppEigen-Introduction.pdf]

Fast and Elegant Numerical Linear Algebra Using the RcppEigen Package

Douglas Bates
University of Wisconsin-Madison

Dirk Eddelbuettel
Debian Project

Abstract

The **RcppEigen** package provides access from R (R Development Core Team 2013) to the **Eigen** (Guennebaud, Jacob *et al.* 2012) C++ template library for numerical linear algebra. **Rcpp** (Eddelbuettel *et al.* 2014; Eddelbuettel 2013) classes and specializations of the C++ templated functions `as` and `wrap` from **Rcpp** provide the "glue" for passing objects from R to C++ and back. Several introductory examples are presented. This is followed by an in-depth discussion of various available approaches for solving least-squares problems, including rank-revealing methods, concluding with an empirical run-time comparison. Last but not least, sparse matrix methods are discussed.

Keywords: linear algebra, template programming, R, C++, **Rcpp**.

This vignette corresponds to a paper published in the *Journal of Statistical Software*. Currently still identical to the paper, this vignette version may over time receive minor updates. For citations, please use Bates and Eddelbuettel (2013) as provided by `citation("RcppEigen")`.

This version corresponds to **RcppEigen** version 0.3.2.2.0 and was typeset on August 21, 2014.

1. Introduction

Linear algebra is an essential building block of statistical computing. Operations such as matrix decompositions, linear program solvers, and eigenvalue/eigenvector computations are used in many estimation and analysis routines. As such, libraries supporting linear algebra have long been provided by statistical programmers for different programming languages and environments. Because it is object-oriented, C++, one of the central modern languages for numerical and statistical computing, is particularly effective at representing matrices, vectors and decompositions, and numerous class libraries providing linear algebra routines have been written over the years.

As both the C++ language and standards have evolved (Meyers 2005, 1995; ISO/IEC 2011), so have the compilers implementing the language. Relatively modern language constructs such as template meta-programming are particularly useful because they provide overloading of operations (allowing expressive code in the compiled language similar to what can be done in scripting languages) and can shift some of the computational burden from the run-time to the compile-time. (A more detailed discussion of template meta-programming is, however, beyond the scope of this paper). Veldhuizen (1998) provided an early and influential implementation of numerical linear algebra classes that already demonstrated key features of this approach.

Its usage was held back at the time by the limited availability of compilers implementing all the necessary features of the C++ language.

This situation has greatly improved over the last decade, and many more libraries have been made available. One such C++ library is **Eigen** by Guennebaud, Jacob *et al.* (2012) which started as a sub-project to KDE (a popular Linux desktop environment), initially focussing on fixed-size matrices to represent projections in a visualization application. **Eigen** grew from there and has over the course of about a decade produced three major releases with **Eigen**3 being the current major version. To check the minor and patch version numbers, load the **RcppEigen** package and call

```
R> .Call("eigen_version", FALSE)

major minor patch
    3     1     1
```

Eigen is of interest as the R system for statistical computation and graphics (R Development Core Team 2013) is itself easily extensible. This is particular true via the C language that most of R's compiled core parts are written in, but also for the C++ language which can interface with C-based systems rather easily. The manual "Writing R Extensions" (R Development Core Team 2012) is the basic reference for extending R with either C or C++.

The **Rcpp** package by Eddelbuettel and François (2011); Eddelbuettel *et al.* (2014) facilitates extending R with C++ code by providing seamless object mapping between both languages. As stated in the **Rcpp** (Eddelbuettel *et al.* 2014) vignette, "Extending **Rcpp**" as well as in Eddelbuettel (2013)

> **Rcpp** facilitates data interchange between R and C++ through the templated functions `Rcpp::as` (for conversion of objects from R to C++) and `Rcpp::wrap` (for conversion from C++ to R).

The **RcppEigen** package provides the header files composing the **Eigen** C++ template library, along with implementations of `Rcpp::as` and `Rcpp::wrap` for the C++ classes defined in **Eigen**.

The **Eigen** classes themselves provide high-performance, versatile and comprehensive representations of dense and sparse matrices and vectors, as well as decompositions and other functions to be applied to these objects. The next section introduces some of these classes and shows how to interface to them from R.

2. Eigen classes

Eigen is a C++ template library providing classes for many forms of matrices, vectors, arrays and decompositions. These classes are flexible and comprehensive allowing for both high performance and well structured code representing high-level operations. C++ code based on **Eigen** is often more like R code, working on the "whole object", than like compiled code in other languages where operations often must be coded in loops.

As in many C++ template libraries using template meta-programming (Abrahams and Gurtovoy 2004), the templates themselves can be very complicated. However, **Eigen** provides

R object type	**Eigen** class typedef
numeric matrix	`MatrixXd`
integer matrix	`MatrixXi`
complex matrix	`MatrixXcd`
numeric vector	`VectorXd`
integer vector	`VectorXi`
complex vector	`VectorXcd`
`Matrix::dgCMatrix`	`SparseMatrix<double>`

Table 1: Correspondence between R matrix and vector types and classes in the **Eigen** namespace.

`typedef`s for common classes that correspond to R matrices and vectors, as shown in Table 1, and this paper will use these `typedef`s.

Here, `Vector` and `Matrix` describe the dimension of the object. The `X` signals that these are dynamically-sized objects (as opposed to fixed-size matrices such as 3×3 matrices also available in **Eigen**). Lastly, the trailing characters `i`, `d` and `cd` denote, respectively, storage types `integer`, `double` and `complex double`.

The C++ classes shown in Table 1 are in the **Eigen** namespace, which means that they must be written as `Eigen::MatrixXd`. However, if one prefaces the use of these class names with a declaration like

```
using Eigen::MatrixXd;
```

then one can use these names without the namespace qualifier.

2.1. Mapped matrices in Eigen

Storage for the contents of matrices from the classes shown in Table 1 is allocated and controlled by the class constructors and destructors. Creating an instance of such a class from an R object involves copying its contents. An alternative is to have the contents of the R matrix or vector mapped to the contents of the object from the **Eigen** class. For dense matrices one can use the **Eigen** templated class `Map`, and for sparse matrices one can deploy the **Eigen** templated class `MappedSparseMatrix`.

One must, of course, be careful not to modify the contents of the R object in the C++ code. A recommended practice is always to declare mapped objects as `const`.

2.2. Arrays in Eigen

For matrix and vector classes **Eigen** overloads the '`*`' operator as matrix multiplication. Occasionally component-wise operations instead of matrix operations are desired, for which the `Array` templated classes are used in **Eigen**. Switching back and forth between matrices or vectors and arrays is usually done via the `array()` method for matrix or vector objects and the `matrix()` method for arrays.

2.3. Structured matrices in Eigen

Eigen provides classes for matrices with special structure such as symmetric matrices, triangular matrices and banded matrices. For dense matrices, these special structures are described as "views", meaning that the full dense matrix is stored but only part of the matrix is used in operations. For a symmetric matrix one must specify whether the lower triangle or the upper triangle is to be used as the contents, with the other triangle defined by the implicit symmetry.

3. Some simple examples

C++ functions to perform simple operations on matrices or vectors can follow a pattern of:

1. Map the R objects passed as arguments into **Eigen** objects.
2. Create the result.
3. Return `Rcpp::wrap` applied to the result.

An idiom for the first step is

```
using Eigen::Map;
using Eigen::MatrixXd;
using Rcpp::as;

const Map<MatrixXd>  A(as<Map<MatrixXd> >(AA));
```

where `AA` is the name of the R object (of type `SEXP` in C and C++) passed to the C++ function.

An alternative to the `using` declarations is to declare a `typedef` as in

```
typedef Eigen::Map<Eigen::MatrixXd>   MapMatd;
const MapMatd          A(Rcpp::as<MapMatd>(AA));
```

The `cxxfunction` function from the **inline** package (Sklyar, Murdoch, Smith, Eddelbuettel, and François 2013) for R and its **RcppEigen** plugin provide a convenient method of developing and debugging the C++ code. For actual production code one generally incorporates the C++ source code files in a package and includes the line `LinkingTo: Rcpp, RcppEigen` in the package's `DESCRIPTION` file. The `RcppEigen.package.skeleton` function provides a quick way of generating the skeleton of a package that will use **RcppEigen**.

The `cxxfunction` with the `"Rcpp"` or `"RcppEigen"` plugins has the `as` and `wrap` functions already defined as `Rcpp::as` and `Rcpp::wrap`. In the examples below these declarations are omitted. It is important to remember that they are needed in actual C++ source code for a package.

The first few examples are simply for illustration as the operations shown could be more effectively performed directly in R. Finally, the results from **Eigen** are compared to those from the direct R results.

3.1. Transpose of an integer matrix

The next R code snippet creates a simple matrix of integers

```
using Eigen::Map;
using Eigen::MatrixXi;
                         // Map the integer matrix AA from R
const Map<MatrixXi>  A(as<Map<MatrixXi> >(AA));
                         // evaluate and return the transpose of A
const MatrixXi      At(A.transpose());
return wrap(At);
```

Figure 1: `transCpp`: Transpose a matrix of integers.

```
R> (A <- matrix(1:6, ncol = 2))

     [,1] [,2]
[1,]    1    4
[2,]    2    5
[3,]    3    6

R> str(A)

 int [1:3, 1:2] 1 2 3 4 5 6
```

and, in Figure 1, the `transpose()` method for the `Eigen::MatrixXi` class is used to return the transpose of the supplied matrix. The R matrix in the `SEXP AA` is first mapped to an `Eigen::MatrixXi` object, and then the matrix `At` is constructed from its transpose and returned to R.

The R snippet below compiles and links the C++ code segment. The actual work is done by the function `cxxfunction` from the **inline** package which compiles, links and loads code written in C++ and supplied as a character variable. This frees the user from having to know about compiler and linker details and options, which makes "exploratory programming" much easier. Here the program piece to be compiled is stored as a character variable named `transCpp`, and `cxxfunction` creates an executable function which is assigned to `ftrans`. This new function is used on the matrix $\boldsymbol{A}$ shown above, and one can check that it works as intended by comparing the output to an explicit transpose of the matrix argument.

```
R> ftrans <- cxxfunction(signature(AA = "matrix"), transCpp,
+    plugin = "RcppEigen")
R> (At <- ftrans(A))

     [,1] [,2] [,3]
[1,]    1    2    3
[2,]    4    5    6

R> stopifnot(all.equal(At, t(A)))
```

For numeric or integer matrices the `adjoint()` method is equivalent to the `transpose()` method. For complex matrices, the adjoint is the conjugate of the transpose. In keeping with

```
typedef Eigen::Map<Eigen::MatrixXi>   MapMati;
const MapMati    B(as<MapMati>(BB));
const MapMati    C(as<MapMati>(CC));
return List::create(Named("B %*% C")         = B * C,
                    Named("crossprod(B, C)") = B.adjoint() * C);
```

Figure 2: `prodCpp`: Product and cross-product of two matrices.

the conventions in the **Eigen** documentation the `adjoint()` method is used in what follows to create the transpose of numeric or integer matrices.

3.2. Products and cross-products

As mentioned in Section 2.2, the '`*`' operator is overloaded as matrix multiplication for the various matrix and vector classes in **Eigen**. The C++ code in Figure 2 produces a list containing both the product and cross-product (in the sense of the R function call `crossproduct(A, B)` evaluating $\boldsymbol{A}^\top \boldsymbol{B}$) of its two arguments

```
R> fprod <- cxxfunction(signature(BB = "matrix", CC = "matrix"),
+    prodCpp, "RcppEigen")
R> B <- matrix(1:4, ncol = 2)
R> C <- matrix(6:1, nrow = 2)
R> str(fp <- fprod(B, C))

List of 2
 $ B %*% C        : int [1:2, 1:3] 21 32 13 20 5 8
 $ crossprod(B, C): int [1:2, 1:3] 16 38 10 24 4 10

R> stopifnot(all.equal(fp[[1]], B %*% C), all.equal(fp[[2]], crossprod(B, C)))
```

Note that the `create` class method for `Rcpp::List` implicitly applies `Rcpp::wrap` to its arguments.

3.3. Crossproduct of a single matrix

As shown in the last example, the R function `crossprod` calculates the product of the transpose of its first argument with its second argument. The single argument form, `crossprod(X)`, evaluates $\boldsymbol{X}^\top \boldsymbol{X}$. One could, of course, calculate this product as

```
t(X) %*% X
```

but `crossprod(X)` is roughly twice as fast because the result is known to be symmetric and only one triangle needs to be calculated. The function `tcrossprod` evaluates `crossprod(t(X))` without actually forming the transpose.

To express these calculations in **Eigen**, a `SelfAdjointView`—which is a dense matrix of which only one triangle is used, the other triangle being inferred from the symmetry—is created. (The characterization "self-adjoint" is equivalent to symmetric for non-complex matrices.)

```
using Eigen::Map;
using Eigen::MatrixXi;
using Eigen::Lower;

const Map<MatrixXi> A(as<Map<MatrixXi> >(AA));
const int           m(A.rows()), n(A.cols());
MatrixXi           AtA(MatrixXi(n, n).setZero().
                       selfadjointView<Lower>().rankUpdate(A.adjoint()));
MatrixXi           AAt(MatrixXi(m, m).setZero().
                       selfadjointView<Lower>().rankUpdate(A));

return List::create(Named("crossprod(A)")  = AtA,
                    Named("tcrossprod(A)") = AAt);
```

Figure 3: `crossprodCpp`: Cross-product and transposed cross-product of a single matrix.

The **Eigen** class name is `SelfAdjointView`. The method for general matrices that produces such a view is called `selfadjointView`. Both require specification of either the `Lower` or `Upper` triangle.

For triangular matrices the class is `TriangularView` and the method is `triangularView`. The triangle can be specified as `Lower`, `UnitLower`, `StrictlyLower`, `Upper`, `UnitUpper` or `StrictlyUpper`.

For self-adjoint views the `rankUpdate` method adds a scalar multiple of $\boldsymbol{A}\boldsymbol{A}^\top$ to the current symmetric matrix. The scalar multiple defaults to 1. The code in Figure 3 produces both $\boldsymbol{A}^\top\boldsymbol{A}$ and $\boldsymbol{A}\boldsymbol{A}^\top$ from a matrix $\boldsymbol{A}$.

```
R> fcprd <- cxxfunction(signature(AA = "matrix"), crossprodCpp, "RcppEigen")
R> str(crp <- fcprd(A))

List of 2
 $ crossprod(A) : int [1:2, 1:2] 14 32 32 77
 $ tcrossprod(A): int [1:3, 1:3] 17 22 27 22 29 36 27 36 45

R> stopifnot(all.equal(crp[[1]], crossprod(A)),
+    all.equal(crp[[2]], tcrossprod(A)))
```

To some, the expressions in Figure 3 to construct `AtA` and `AAt` are compact and elegant. To others they are hopelessly confusing. If you find yourself in the latter group, you just need to read the expression left to right. So, for example, we construct `AAt` by creating a general integer matrix of size $m \times m$ (where $\boldsymbol{A}$ is $m \times n$), ensuring that all its elements are zero, regarding it as a self-adjoint (i.e., symmetric) matrix using the elements in the lower triangle, then adding $\boldsymbol{A}\boldsymbol{A}^\top$ to it and converting back to a general matrix form (i.e., the strict lower triangle is copied into the strict upper triangle).

In more detail:

1. `MatrixXi(n, n)` creates an $n \times n$ integer matrix with arbitrary contents

```
using   Eigen::LLT;
using   Eigen::Lower;
using   Eigen::Map;
using   Eigen::MatrixXd;
using   Eigen::MatrixXi;
using   Eigen::Upper;
using   Eigen::VectorXd;
typedef Map<MatrixXd>  MapMatd;
typedef Map<MatrixXi>  MapMati;
typedef Map<VectorXd>  MapVecd;
inline MatrixXd AtA(const MapMatd& A) {
    int    n(A.cols());
    return   MatrixXd(n,n).setZero().selfadjointView<Lower>()
             .rankUpdate(A.adjoint());
}
```

Figure 4: The contents of the character vector, `incl`, that will preface C++ code segments that follow.

2. `.setZero()` zeros out the contents of the matrix
3. `.selfAdjointView<Lower>()` causes what follows to treat the matrix as a symmetric matrix in which only the lower triangle is used, the strict upper triangle being inferred by symmetry
4. `.rankUpdate(A)` forms the sum $\boldsymbol{B} + \boldsymbol{A}\boldsymbol{A}^\top$ where $\boldsymbol{B}$ is the symmetric matrix of zeros created in the previous steps.

The assignment of this symmetric matrix to the (general) `MatrixXi` object `AAt` causes the result to be symmetrized during the assignment.

For these products one could define the symmetric matrix from either the lower triangle or the upper triangle as the result will be symmetrized before it is returned.

To cut down on repetition of `using` statements we gather them in a character variable, `incl`, that will be given as the `includes` argument in the calls to `cxxfunction`. We also define a utility function, `AtA`, that returns the crossproduct matrix as shown in Figure 4

3.4. Cholesky decomposition of the crossprod

The Cholesky decomposition of the positive-definite, symmetric matrix, $\boldsymbol{A}$, can be written in several forms. Numerical analysts define the "LLt" form as the lower triangular matrix, $\boldsymbol{L}$, such that $\boldsymbol{A} = \boldsymbol{L}\boldsymbol{L}^\top$ and the "LDLt" form as a unit lower triangular matrix $\boldsymbol{L}$ and a diagonal matrix $\boldsymbol{D}$ with positive diagonal elements such that $\boldsymbol{A} = \boldsymbol{L}\boldsymbol{D}\boldsymbol{L}^\top$. Statisticians often write the decomposition as $\boldsymbol{A} = \boldsymbol{R}^\top\boldsymbol{R}$ where $\boldsymbol{R}$ is an upper triangular matrix. Of course, this $\boldsymbol{R}$ is simply the transpose of $\boldsymbol{L}$ from the "LLt" form.

The templated **Eigen** classes for the LLt and LDLt forms are called `LLT` and `LDLT` and the corresponding methods are `.llt()` and `.ldlt()`. Because the Cholesky decomposition involves taking square roots, we pass a numeric matrix, $\boldsymbol{A}$, not an integer matrix.

```
const  LLT<MatrixXd> llt(AtA(as<MapMatd>(AA)));
return List::create(Named("L") = MatrixXd(llt.matrixL()),
                    Named("R") = MatrixXd(llt.matrixU()));
```

Figure 5: `cholCpp`: Cholesky decomposition of a cross-product.

```
R> storage.mode(A) <- "double"
R> fchol <- cxxfunction(signature(AA = "matrix"), cholCpp, "RcppEigen", incl)
R> (ll <- fchol(A))

$L
        [,1]    [,2]
[1,] 3.74166 0.00000
[2,] 8.55236 1.96396

$R
        [,1]    [,2]
[1,] 3.74166 8.55236
[2,] 0.00000 1.96396

R> stopifnot(all.equal(ll[[2]], chol(crossprod(A))))
```

3.5. Determinant of the cross-product matrix

The "D-optimal" criterion for experimental design chooses the design that maximizes the determinant, $|\boldsymbol{X}^\top\boldsymbol{X}|$, for the $n \times p$ model matrix (or Jacobian matrix), $\boldsymbol{X}$. The determinant, $|\boldsymbol{L}|$, of the $p \times p$ lower Cholesky factor $\boldsymbol{L}$, defined so that $\boldsymbol{L}\boldsymbol{L}^\top = \boldsymbol{X}^\top\boldsymbol{X}$, is the product of its diagonal elements, as is the case for any triangular matrix. By the properties of determinants,

$$|\boldsymbol{X}^\top\boldsymbol{X}| = |\boldsymbol{L}\boldsymbol{L}^\top| = |\boldsymbol{L}|\,|\boldsymbol{L}^\top| = |\boldsymbol{L}|^2$$

Alternatively, if using the "LDLt" decomposition, $\boldsymbol{L}\boldsymbol{D}\boldsymbol{L}^\top = \boldsymbol{X}^\top\boldsymbol{X}$ where $\boldsymbol{L}$ is unit lower triangular and $\boldsymbol{D}$ is diagonal then $|\boldsymbol{X}^\top\boldsymbol{X}|$ is the product of the diagonal elements of $\boldsymbol{D}$. Because it is known that the diagonal elements of $\boldsymbol{D}$ must be non-negative, one often evaluates the logarithm of the determinant as the sum of the logarithms of the diagonal elements of $\boldsymbol{D}$. Several options are shown in Figure 6.

```
R> fdet <- cxxfunction(signature(AA = "matrix"), cholDetCpp, "RcppEigen",
+    incl)
R> unlist(ll <- fdet(A))

      d1       d2       ld
54.00000 54.00000  3.98898
```

Note the use of the `array()` method in the calculation of the log-determinant. Because the `log()` method applies to arrays, not to vectors or matrices, an array must be created from `Dvec` before applying the `log()` method.

```
const MatrixXd  ata(AtA(as<MapMatd>(AA)));
const double   detL(MatrixXd(ata.llt().matrixL()).diagonal().prod());
const VectorXd Dvec(ata.ldlt().vectorD());
return List::create(Named("d1") = detL * detL,
                    Named("d2") = Dvec.prod(),
                    Named("ld") = Dvec.array().log().sum());
```

Figure 6: `cholDetCpp`: Determinant of a cross-product using the "LLt" and "LDLt" forms of the Cholesky decomposition.

4. Least squares solutions

A common operation in statistical computing is calculating a least squares solution, $\widehat{\boldsymbol{\beta}}$, defined as

$$\widehat{\boldsymbol{\beta}} = \underset{\boldsymbol{\beta}}{\operatorname{argmin}} \|\boldsymbol{y} - \boldsymbol{X}\boldsymbol{\beta}\|^2$$

where the model matrix, $\boldsymbol{X}$, is $n \times p$ ($n \geq p$) and $\boldsymbol{y}$ is an n-dimensional response vector. There are several ways, based on matrix decompositions, to determine such a solution. Earlier, two forms of the Cholesky decomposition were discussed: "LLt" and "LDLt", which can both be used to solve for $\widehat{\boldsymbol{\beta}}$. Other decompositions that can be used are the QR decomposition, with or without column pivoting, the singular value decomposition and the eigendecomposition of a symmetric matrix.

Determining a least squares solution is relatively straightforward. However, statistical computing often requires additional information, such as the standard errors of the coefficient estimates. Calculating these involves evaluating the diagonal elements of $\left(\boldsymbol{X}^\top\boldsymbol{X}\right)^{-1}$ and the residual sum of squares, $\|\boldsymbol{y} - \boldsymbol{X}\widehat{\boldsymbol{\beta}}\|^2$.

4.1. Least squares using the "LLt" Cholesky

Figure 7 shows a calculation of the least squares coefficient estimates (`betahat`) and the standard errors (`se`) through an "LLt" Cholesky decomposition of the crossproduct of the model matrix, $\boldsymbol{X}$. Next, the results from this calculation are compared to those from the `lm.fit` function in R (`lm.fit` is the workhorse function called by `lm` once the model matrix and response have been evaluated).

```
R> lltLS <- cxxfunction(signature(XX = "matrix", yy = "numeric"), lltLSCpp,
+    "RcppEigen", incl)
R> data("trees", package = "datasets")
R> str(lltFit <- with(trees, lltLS(cbind(1, log(Girth)), log(Volume))))

List of 7
 $ coefficients : num [1:2] -2.35 2.2
 $ fitted.values: num [1:31] 2.3 2.38 2.43 2.82 2.86 ...
 $ residuals    : num [1:31] 0.0298 -0.0483 -0.1087 -0.0223 0.0727 ...
 $ s            : num 0.115
 $ df.residual  : int 29
```

```
const MapMatd          X(as<MapMatd>(XX));
const MapVecd          y(as<MapVecd>(yy));
const int              n(X.rows()), p(X.cols());
const LLT<MatrixXd> llt(AtA(X));
const VectorXd  betahat(llt.solve(X.adjoint() * y));
const VectorXd   fitted(X * betahat);
const VectorXd    resid(y - fitted);
const int            df(n - p);
const double          s(resid.norm() / std::sqrt(double(df)));
const VectorXd       se(s * llt.matrixL().solve(MatrixXd::Identity(p, p))
                          .colwise().norm());
return     List::create(Named("coefficients")   = betahat,
                        Named("fitted.values")  = fitted,
                        Named("residuals")      = resid,
                        Named("s")              = s,
                        Named("df.residual")    = df,
                        Named("rank")           = p,
                        Named("Std. Error")     = se);
```

Figure 7: `lltLSCpp`: Least squares using the Cholesky decomposition.

```
 $ rank          : int 2
 $ Std. Error    : num [1:2] 0.2307 0.0898
R> str(lmFit <- with(trees, lm.fit(cbind(1, log(Girth)), log(Volume))))
List of 8
 $ coefficients : Named num [1:2] -2.35 2.2
  ..- attr(*, "names")= chr [1:2] "x1" "x2"
 $ residuals    : num [1:31] 0.0298 -0.0483 -0.1087 -0.0223 0.0727 ...
 $ effects      : Named num [1:31] -18.2218 2.8152 -0.1029 -0.0223 0.0721 ...
  ..- attr(*, "names")= chr [1:31] "x1" "x2" "" "" ...
 $ rank         : int 2
 $ fitted.values: num [1:31] 2.3 2.38 2.43 2.82 2.86 ...
 $ assign       : NULL
 $ qr           :List of 5
  ..$ qr   : num [1:31, 1:2] -5.57 0.18 0.18 0.18 0.18 ...
  ..$ qraux: num [1:2] 1.18 1.26
  ..$ pivot: int [1:2] 1 2
  ..$ tol  : num 1e-07
  ..$ rank : int 2
  ..- attr(*, "class")= chr "qr"
 $ df.residual  : int 29
```

```
R> for(nm in c("coefficients", "residuals", "fitted.values", "rank"))
+    stopifnot(all.equal(lltFit[[nm]], unname(lmFit[[nm]])))
R> stopifnot(all.equal(lltFit[["Std. Error"]],
+    unname(coef(summary(lm(log(Volume) ~ log(Girth), trees)))[,2])))
```

```
using Eigen::HouseholderQR;

const HouseholderQR<MatrixXd> QR(X);
const VectorXd betahat(QR.solve(y));
const VectorXd  fitted(X * betahat);
const int           df(n - p);
const VectorXd      se(QR.matrixQR().topRows(p).triangularView<Upper>()
                       .solve(MatrixXd::Identity(p,p)).rowwise().norm());
```

Figure 8: `QRLSCpp`: Least squares using the unpivoted QR decomposition.

There are several aspects of the C++ code in Figure 7 worth mentioning. The `solve` method for the `LLT` object evaluates, in this case, $\left(\boldsymbol{X}^\top\boldsymbol{X}\right)^{-1}\boldsymbol{X}^\top\boldsymbol{y}$ but without actually evaluating the inverse. The calculation of the residuals, $\boldsymbol{y}-\widehat{\boldsymbol{y}}$, can be written, as in R, as `y - fitted`. (But note that **Eigen** classes do not have a "recycling rule" as in R. That is, the two vector operands must have the same length.) The `norm()` method evaluates the square root of the sum of squares of the elements of a vector. Although one does not explicitly evaluate $\left(\boldsymbol{X}^\top\boldsymbol{X}\right)^{-1}$ one does evaluate $\boldsymbol{L}^{-1}$ to obtain the standard errors. Note also the use of the `colwise()` method in the evaluation of the standard errors. It applies a method to the columns of a matrix, returning a vector. The **Eigen** `colwise()` and `rowwise()` methods are similar in effect to the `apply` function in R.

In the descriptions of other methods for solving least squares problems, much of the code parallels that shown in Figure 7. The redundant parts are omitted, and only the evaluation of the coefficients, the rank and the standard errors is shown. Actually, the standard errors are calculated only up to the scalar multiple of s, the residual standard error, in these code fragments. The calculation of the residuals and s and the scaling of the coefficient standard errors is the same for all methods. (See the files `fastLm.h` and `fastLm.cpp` in the **RcppEigen** source package for details.)

4.2. Least squares using the unpivoted QR decomposition

A QR decomposition has the form

$$\boldsymbol{X}=\boldsymbol{Q}\boldsymbol{R}=\boldsymbol{Q}_1\boldsymbol{R}_1$$

where $\boldsymbol{Q}$ is an $n\times n$ orthogonal matrix, which means that $\boldsymbol{Q}^\top\boldsymbol{Q}=\boldsymbol{Q}\boldsymbol{Q}^\top=\boldsymbol{I}_n$, and the $n\times p$ matrix $\boldsymbol{R}$ is zero below the main diagonal. The $n\times p$ matrix $\boldsymbol{Q}_1$ is the first p columns of $\boldsymbol{Q}$ and the $p\times p$ upper triangular matrix $\boldsymbol{R}_1$ is the top p rows of $\boldsymbol{R}$. There are three **Eigen** classes for the QR decomposition: `HouseholderQR` provides the basic QR decomposition using Householder transformations, `ColPivHouseholderQR` incorporates column pivots and `FullPivHouseholderQR` incorporates both row and column pivots.

Figure 8 shows a least squares solution using the unpivoted QR decomposition. The calculations in Figure 8 are quite similar to those in Figure 7. In fact, if one had extracted the upper triangular factor (the `matrixU()` method) from the `LLT` object in Figure 7, the rest of the code would be nearly identical.

4.3. Handling the rank-deficient case

One important consideration when determining least squares solutions is whether rank($\boldsymbol{X}$) is p, a situation described by saying that $\boldsymbol{X}$ has "full column rank". When $\boldsymbol{X}$ does not have full column rank it is said to be "rank deficient".

Although the theoretical rank of a matrix is well-defined, its evaluation in practice is not. At best one can compute an effective rank according to some tolerance. Decompositions that allow to estimation of the rank of the matrix in this way are said to be "rank-revealing".

Because the `model.matrix` function in R does a considerable amount of symbolic analysis behind the scenes, one usually ends up with full-rank model matrices. The common cases of rank-deficiency, such as incorporating both a constant term and a full set of indicators columns for the levels of a factor, are eliminated. Other, more subtle, situations will not be detected at this stage, however. A simple example occurs when there is a "missing cell" in a two-way layout and the interaction of the two factors is included in the model.

```
R> dd <- data.frame(f1 = gl(4, 6, labels = LETTERS[1:4]),
+    f2 = gl(3, 2, labels = letters[1:3]))[-(7:8), ]
R> xtabs(~ f2 + f1, dd)

   f1
f2  A B C D
  a 2 0 2 2
  b 2 2 2 2
  c 2 2 2 2

R> mm <- model.matrix(~ f1 * f2, dd)
R> kappa(mm)

[1] 4.30923e+16
```

This yields a large condition number, indicating rank deficiency. Alternatively, the reciprocal condition number can be evaluated.

```
R> rcond(mm)

[1] 2.3206e-17

R> (c(rank = qr(mm)$rank, p = ncol(mm)))

rank    p
  11   12

R> set.seed(1)
R> dd$y <- mm %*% seq_len(ncol(mm)) + rnorm(nrow(mm), sd = 0.1)
R> fm1 <- lm(y ~ f1 * f2, dd)
R> writeLines(capture.output(print(summary(fm1),
+    signif.stars = FALSE))[9:22])
```

```
Coefficients: (1 not defined because of singularities)
            Estimate Std. Error t value Pr(>|t|)
(Intercept)   0.9779     0.0582    16.8  3.4e-09
f1B          12.0381     0.0823   146.3  < 2e-16
f1C           3.1172     0.0823    37.9  5.2e-13
f1D           4.0685     0.0823    49.5  2.8e-14
f2b           5.0601     0.0823    61.5  2.6e-15
f2c           5.9976     0.0823    72.9  4.0e-16
f1B:f2b      -3.0148     0.1163   -25.9  3.3e-11
f1C:f2b       7.7030     0.1163    66.2  1.2e-15
f1D:f2b       8.9643     0.1163    77.1  < 2e-16
f1B:f2c           NA         NA      NA       NA
f1C:f2c      10.9613     0.1163    94.2  < 2e-16
f1D:f2c      12.0411     0.1163   103.5  < 2e-16
```

The `lm` function for fitting linear models in R uses a rank-revealing form of the QR decomposition. When the model matrix is determined to be rank deficient, according to the threshold used in R's `qr` function, the model matrix is reduced to rank $(\boldsymbol{X})$ columns by pivoting selected columns (those that are apparently linearly dependent on columns to their left) to the right hand side of the matrix. A solution for this reduced model matrix is determined and the coefficients and standard errors for the redundant columns are flagged as missing.

An alternative approach is to evaluate the "pseudo-inverse" of $\boldsymbol{X}$ from the singular value decomposition (SVD) of $\boldsymbol{X}$ or the eigendecomposition of $\boldsymbol{X}^\top\boldsymbol{X}$. The SVD is of the form

$$\boldsymbol{X} = \boldsymbol{U}\boldsymbol{D}\boldsymbol{V}^\top = \boldsymbol{U}_1\boldsymbol{D}_1\boldsymbol{V}^\top$$

where $\boldsymbol{U}$ is an orthogonal $n \times n$ matrix and $\boldsymbol{U}_1$ is its leftmost p columns, $\boldsymbol{D}$ is $n \times p$ and zero off the main diagonal so that $\boldsymbol{D}_1$ is a $p \times p$ diagonal matrix with non-increasing, non-negative diagonal elements, and $\boldsymbol{V}$ is a $p \times p$ orthogonal matrix. The pseudo-inverse of $\boldsymbol{D}_1$, written $\boldsymbol{D}_1^+$ is a $p \times p$ diagonal matrix whose first $r = \operatorname{rank}(\boldsymbol{X})$ diagonal elements are the inverses of the corresponding diagonal elements of $\boldsymbol{D}_1$ and whose last $p - r$ diagonal elements are zero. The tolerance for determining if an element of the diagonal of $\boldsymbol{D}_1$ is considered to be (effectively) zero is a multiple of the largest singular value (i.e., the $(1,1)$ element of $\boldsymbol{D}$). The pseudo-inverse, $\boldsymbol{X}^+$, of $\boldsymbol{X}$ is defined as

$$\boldsymbol{X}^+ = \boldsymbol{V}\boldsymbol{D}_1^+\boldsymbol{U}_1^\top.$$

In Figure 9 a utility function, `Dplus`, is defined to return the diagonal of the pseudo-inverse, $\boldsymbol{D}_1^+$, as an array, given the singular values (the diagonal of $\boldsymbol{D}_1$) as an array. Calculation of the maximum element of $\boldsymbol{d}$ (the method is called `.maxCoeff()`) and the use of a `threshold()` function provides greater generality for the function. It can be used on the eigenvalues of $\boldsymbol{X}^\top\boldsymbol{X}$, as shown in Section 4.5, even though these are returned in increasing order, as opposed to the decreasing order of the singular values.

4.4. Least squares using the SVD

With these definitions the code for least squares using the singular value decomposition can be written as in Figure 10. Even in the rank-deficient case this code will produce a complete set

```
inline ArrayXd Dplus(const ArrayXd& d) {
    ArrayXd   di(d.size());
    double  comp(d.maxCoeff() * threshold());
    for (int j = 0; j < d.size(); ++j) di[j] = (d[j] < comp) ? 0. : 1./d[j];
    return di;
}
```

Figure 9: `DplusCpp`: Create the diagonal $\boldsymbol{d}^+$ of the pseudo-inverse, $\boldsymbol{D}_1^+$, from the array of singular values, $\boldsymbol{d}$.

of coefficients and their standard errors. The user must be careful to check if the computed rank is less than p, the number of columns in $\boldsymbol{X}$, in which case the estimated coefficients are just one of an infinite number of coefficient vectors that produce the same fitted values. It happens that the solution from this pseudo-inverse is the minimum norm solution (in the sense that $\|\boldsymbol{\beta}\|^2$ is minimized among those $\boldsymbol{\beta}$ that minimize $\|\boldsymbol{y} - \boldsymbol{X\beta}\|^2$).

The standard errors of the coefficient estimates in the rank-deficient case must be interpreted carefully. The solution with one or more missing coefficients, as returned by the `lm.fit` function in R and by the column-pivoted QR decomposition described in Section 4.6, does not provide standard errors for the missing coefficients. That is, both the coefficient and its standard error are returned as `NA` because the least squares solution is performed on a reduced model matrix. It is also true that the solution returned by the SVD method is with respect to a reduced model matrix but the p coefficient estimates and their p standard errors don't show this. They are, in fact, linear combinations of a set of r coefficient estimates and their standard errors.

```
const Eigen::JacobiSVD<MatrixXd>
          UDV(X.jacobiSvd(Eigen::ComputeThinU|Eigen::ComputeThinV));
const ArrayXd       Dp(Dplus(UDV.singularValues()));
const int            r((Dp > 0).count());
const MatrixXd     VDp(UDV.matrixV() * Dp.matrix().asDiagonal());
const VectorXd betahat(VDp * UDV.matrixU().adjoint() * y);
const VectorXd      se(s * VDp.rowwise().norm());
```

Figure 10: `SVDLSCpp`: Least squares using the SVD.

```
const Eigen::SelfAdjointEigenSolver<MatrixXd> VLV(AtA(X));
const ArrayXd       Dp(Dplus(eig.eigenvalues()).sqrt());
const int            r((Dp > 0).count());
const MatrixXd     VDp(VLV.eigenvectors() * Dp.matrix().asDiagonal());
const VectorXd betahat(VDp * VDp.adjoint() * X.adjoint() * y);
const VectorXd      se(s * VDp.rowwise().norm());
```

Figure 11: `SymmEigLSCpp`: Least squares using the eigendecomposition.

4.5. Least squares using the eigendecomposition

The eigendecomposition of $\boldsymbol{X}^\top\boldsymbol{X}$ is defined as

$$\boldsymbol{X}^\top\boldsymbol{X} = \boldsymbol{V}\boldsymbol{\Lambda}\boldsymbol{V}^\top$$

where $\boldsymbol{V}$, the matrix of eigenvectors, is a $p \times p$ orthogonal matrix and $\boldsymbol{\Lambda}$ is a $p \times p$ diagonal matrix with non-decreasing, non-negative diagonal elements, called the eigenvalues of $\boldsymbol{X}^\top\boldsymbol{X}$. When the eigenvalues are distinct, this $\boldsymbol{V}$ is the same (up to reordering of the columns) as that in the SVD and the eigenvalues of $\boldsymbol{X}^\top\boldsymbol{X}$ are the (reordered) squares of the singular values of $\boldsymbol{X}$.

With these definitions one can adapt much of the code from the SVD method for the eigendecomposition, as shown in Figure 11.

4.6. Least squares using the column-pivoted QR decomposition

The column-pivoted QR decomposition provides results similar to those from R in both the full-rank and the rank-deficient cases. The decomposition is of the form

$$\boldsymbol{X}\boldsymbol{P} = \boldsymbol{Q}\boldsymbol{R} = \boldsymbol{Q}_1\boldsymbol{R}_1$$

where, as before, $\boldsymbol{Q}$ is $n \times n$ and orthogonal and $\boldsymbol{R}$ is $n \times p$ and upper triangular. The $p \times p$ matrix $\boldsymbol{P}$ is a permutation matrix. That is, its columns are a permutation of the columns of $\boldsymbol{I}_p$. It serves to reorder the columns of $\boldsymbol{X}$ so that the diagonal elements of $\boldsymbol{R}$ are non-increasing in magnitude.

An instance of the class `Eigen::ColPivHouseholderQR` has a `rank()` method returning the computational rank of the matrix. When $\boldsymbol{X}$ is of full rank one can use essentially the same code as in the unpivoted decomposition except that one must reorder the standard errors. When $\boldsymbol{X}$ is rank-deficient, the coefficients and standard errors are evaluated for the leading r columns of $\boldsymbol{X}\boldsymbol{P}$ only.

In the rank-deficient case the straightforward calculation of the fitted values, as $\boldsymbol{X}\widehat{\boldsymbol{\beta}}$, cannot be used because some of the estimated coefficients, $\widehat{\boldsymbol{\beta}}$, are `NA` so the product will be a vector of `NA`'s. One could do some complicated rearrangement of the columns of X and the coefficient estimates but it is conceptually (and computationally) easier to employ the relationship

$$\widehat{\boldsymbol{y}} = \boldsymbol{Q}_1\boldsymbol{Q}_1^\top\boldsymbol{y} = \boldsymbol{Q}\begin{bmatrix}\boldsymbol{I}_r & \boldsymbol{0}\\ \boldsymbol{0} & \boldsymbol{0}\end{bmatrix}\boldsymbol{Q}^\top\boldsymbol{y}$$

The vector $\boldsymbol{Q}^\top\boldsymbol{y}$ is called the "effects" vector in R.

Just to check that the code in Figure 12 does indeed provide the desired answer

```
typedef Eigen::ColPivHouseholderQR<MatrixXd>  CPivQR;
typedef CPivQR::PermutationType              Permutation;

const CPivQR       PQR(X);
const Permutation Pmat(PQR.colsPermutation());
const int          r(PQR.rank());
VectorXd       betahat, fitted, se;
if (r == X.cols()) { // full rank case
    betahat = PQR.solve(y);
    fitted  = X * betahat;
    se      = Pmat * PQR.matrixQR().topRows(p).triangularView<Upper>()
                      .solve(MatrixXd::Identity(p, p)).rowwise().norm();
} else {
    MatrixXd    Rinv(PQR.matrixQR().topLeftCorner(r, r)
         .triangularView<Upper>().solve(MatrixXd::Identity(r, r)));
    VectorXd effects(PQR.householderQ().adjoint() * y);
    betahat.fill(::NA_REAL);
    betahat.head(r)  = Rinv * effects.head(r);
    betahat          = Pmat * betahat;
    se.fill(::NA_REAL);
    se.head(r)       = Rinv.rowwise().norm();
    se               = Pmat * se;
                      // create fitted values from effects
    effects.tail(X.rows() - r).setZero();
    fitted           = PQR.householderQ() * effects;
}
```

Figure 12: ColPivQRLSCpp: Least squares using the pivoted QR decomposition.

```
R> print(summary(fmPQR <- fastLm(y ~ f1 * f2, dd)), signif.st = FALSE)

Call:
fastLm.formula(formula = y ~ f1 * f2, data = dd)

             Estimate Std. Error t value  Pr(>|t|)
(Intercept)  0.977859   0.058165  16.812 3.413e-09
f1B         12.038068   0.082258 146.346 < 2.2e-16
f1C          3.117222   0.082258  37.896 5.221e-13
f1D          4.068523   0.082258  49.461 2.833e-14
f2b          5.060123   0.082258  61.516 2.593e-15
f2c          5.997592   0.082258  72.912 4.015e-16
f1B:f2b     -3.014763   0.116330 -25.916 3.266e-11
f1C:f2b      7.702999   0.116330  66.217 1.156e-15
f1D:f2b      8.964251   0.116330  77.059 < 2.2e-16
f1B:f2c            NA         NA      NA        NA
f1C:f2c     10.961326   0.116330  94.226 < 2.2e-16
```

```
f1D:f2c     12.041081   0.116330 103.508 < 2.2e-16

Residual standard error: 0.2868 on 11 degrees of freedom
Multiple R-squared: 0.9999,       Adjusted R-squared: 0.9999
```

```
R> all.equal(coef(fm1), coef(fmPQR))
```

```
[1] TRUE
```

```
R> all.equal(unname(fitted(fm1)), fitted(fmPQR))
```

```
[1] TRUE
```

```
R> all.equal(unname(residuals(fm1)), residuals(fmPQR))
```

```
[1] TRUE
```

The rank-revealing SVD method produces the same fitted values but not the same coefficients.

```
R> print(summary(fmSVD <- fastLm(y ~ f1 * f2, dd, method = 4)),
+    signif.st = FALSE)
```

```
Call:
fastLm.formula(formula = y ~ f1 * f2, data = dd, method = 4)

             Estimate Std. Error t value  Pr(>|t|)
(Intercept)  0.977859   0.058165  16.812 3.413e-09
f1B          7.020458   0.038777 181.049 < 2.2e-16
f1C          3.117222   0.082258  37.896 5.221e-13
f1D          4.068523   0.082258  49.461 2.833e-14
f2b          5.060123   0.082258  61.516 2.593e-15
f2c          5.997592   0.082258  72.912 4.015e-16
f1B:f2b      2.002847   0.061311  32.667 2.638e-12
f1C:f2b      7.702999   0.116330  66.217 1.156e-15
f1D:f2b      8.964251   0.116330  77.059 < 2.2e-16
f1B:f2c      5.017610   0.061311  81.838 < 2.2e-16
f1C:f2c     10.961326   0.116330  94.226 < 2.2e-16
f1D:f2c     12.041081   0.116330 103.508 < 2.2e-16

Residual standard error: 0.2868 on 11 degrees of freedom
Multiple R-squared: 0.9999,       Adjusted R-squared: 0.9999
```

```
R> all.equal(coef(fm1), coef(fmSVD))
```

```
[1] "'is.NA' value mismatch: 0 in current 1 in target"
```

```
R> all.equal(unname(fitted(fm1)), fitted(fmSVD))
```

```
[1] TRUE
```

```
R> all.equal(unname(residuals(fm1)), residuals(fmSVD))
```

```
[1] TRUE
```

The coefficients from the symmetric eigendecomposition method are the same as those from the SVD, hence the fitted values and residuals must be the same for these two methods.

```
R> summary(fmVLV <- fastLm(y ~ f1 * f2, dd, method = 5))
R> all.equal(coef(fmSVD), coef(fmVLV))
```

```
[1] TRUE
```

4.7. Comparative speed

In the **RcppEigen** package the R function to fit linear models using the methods described above is called `fastLm`. It follows an earlier example in the **Rcpp** package which was carried over to both **RcppArmadillo** and **RcppGSL**. The natural question to ask is, "Is it indeed fast to use these methods based on **Eigen**?". To this end, the package provides benchmarking code for these methods, R's `lm.fit` function and the `fastLm` implementations in the **RcppArmadillo** (François *et al.* 2014) and **RcppGSL** (François and Eddelbuettel 2012) packages, if they are installed. The benchmark code, which uses the **rbenchmark** (Kusnierczyk 2012) package, is in a file named `lmBenchmark.R` in the `examples` subdirectory of the installed **RcppEigen** package.

It can be run as

```
R> source(system.file("examples", "lmBenchmark.R", package = "RcppEigen"))
```

Results will vary according to the speed of the processor and the implementation of the BLAS (Basic Linear Algebra Subroutines) used. (**Eigen** methods do not use the BLAS but the other methods do.) The **Eigen**3 template library does not use multi-threaded code for these operations but does use the graphics pipeline instructions (SSE and SSE2, in this case) in some calculations.

Results obtained on a desktop computer, circa 2010, are shown in Table 2. The processor used for these timings is a 4-core processor but almost all the methods are single-threaded and not affected by the number of cores. Only the `arma`, `lm.fit`, `GESDD` and `GSL` methods benefit from the multi-threaded BLAS implementation provided by OpenBLAS, and the relative speed increase is modest for this problem size and number of cores (at 7.76 seconds relative to 10.29 seconds for `arma`, 13.74 seconds relative to 16.91 seconds for `lm.fit`, and 176.95 seconds relative to 193.29 seconds for the `GSL`). Parallel computing approaches will always have to trade-off increased communication and overhead costs against the potential gains from running multiple execution threads.

These results indicate that methods based on forming and decomposing $\boldsymbol{X}^\top\boldsymbol{X}$ (LDLt, LLt and SymmEig) are considerably faster than the others. The SymmEig method, using a rank-revealing decomposition, would be preferred, although the LDLt method could probably be

Method	Relative	Elapsed	User	Sys
LDLt	1.00	1.18	1.17	0.00
LLt	1.01	1.19	1.17	0.00
SymmEig	2.76	3.25	2.70	0.52
QR	6.35	7.47	6.93	0.53
arma	6.60	7.76	25.69	4.47
PivQR	7.15	8.41	7.78	0.62
lm.fit	11.68	13.74	21.56	16.79
GESDD	12.58	14.79	44.01	10.96
SVD	44.48	52.30	51.38	0.80
GSL	150.46	176.95	210.52	149.86

Table 2: `lmBenchmark` results on a desktop computer for the default size, $100,000 \times 40$, full-rank model matrix running 20 repetitions for each method. Times (Elapsed, User, and Sys) are in seconds. The BLAS in use is a locally-rebuilt version of the OpenBLAS library included with Ubuntu 11.10.

modified to be rank-revealing. However, the dimensions of the problem will influence the comparative results. Because there are 100,000 rows in $\boldsymbol{X}$, methods that decompose the whole $\boldsymbol{X}$ matrix (all the methods except those named above) will be at a disadvantage.

The pivoted QR method is 1.6 times faster than R's `lm.fit` on this test and provides nearly the same information as `lm.fit`. Methods based on the singular value decomposition (SVD and GSL) are much slower but, as mentioned above, this is caused in part by $\boldsymbol{X}$ having many more rows than columns. The GSL method from the GNU Scientific Library uses an older algorithm for the SVD and is clearly out of contention.

The `GESDD` method provides an interesting hybrid: It uses the **Eigen** classes, but then deploys the LAPACK routine `dgesdd` for the actual SVD calculation. The resulting time is much faster than using the SVD implementation of **Eigen** which is not a particularly fast SVD method.

5. Delayed evaluation

A form of delayed evaluation is used in **Eigen**. That is, many operators and methods do not evaluate the result but instead return an "expression object" that is evaluated when needed. As an example, even though one writes the $\boldsymbol{X}^\top\boldsymbol{X}$ evaluation as `.rankUpdate(X.adjoint())` the `X.adjoint()` part is not evaluated immediately. The `rankUpdate` method detects that it has been passed a matrix that is to be used in its transposed form and evaluates the update by taking inner products of columns of $\boldsymbol{X}$ instead of rows of $\boldsymbol{X}^\top$.

As **Eigen** has developed some of these unevaluated expressions have been modified. In **Eigen 3.1**, which is incorporated in version 0.3.1 of **RcppEigen**, the `.adjoint()` method applied to a real dense matrix copies the contents of the original matrix, flags it as row-major and interchanges the number of rows and columns. This is indeed the adjoint of the original matrix but, at one time, the `wrap` method for the **Eigen** dense matrix classes would fail on row-major matrices.

In the code for the transpose of an integer matrix shown in Figure 1 the transpose is assigned

```
const MapMati  A(as<MapMati>(AA));
return wrap(A.transpose());
```

Figure 13: `badtransCpp`: Transpose producing a run-time error in early versions of **RcppEigen**.

to a `MatrixXi` object before applying `wrap` to it. The assignment forces the evaluation as a column-major matrix. In early versions of the **RcppEigen** package if this step is skipped, as in Figure 13, the result would have been incorrect.

```
R> Ai <- matrix(1:6, ncol = 2L)
R> ftrans2 <- cxxfunction(signature(AA = "matrix"), badtransCpp,
+    "RcppEigen", incl)
R> (At <- ftrans2(Ai))
```

```
     [,1] [,2] [,3]
[1,]    1    2    3
[2,]    4    5    6
```

Although the problem no longer persists for this particular example, the recommended practice is to first assign objects before wrapping them for return to R.

6. Sparse matrices

Eigen provides sparse matrix classes. An R object of class `dgCMatrix` (from the **Matrix** package by Bates and Maechler (2014)) can be mapped as in Figure 14.

```
R> sparse1 <- cxxfunction(signature(AA = "dgCMatrix", yy = "numeric"),
+    sparseProdCpp, "RcppEigen", incl)
R> data("KNex", package = "Matrix")
R> rr <- sparse1(KNex$mm, KNex$y)
R> stopifnot(all.equal(rr$At, t(KNex$mm)),
+    all.equal(rr$Aty, as.vector(crossprod(KNex$mm, KNex$y))))
```

```
using Eigen::MappedSparseMatrix;
using Eigen::SparseMatrix;

const MappedSparseMatrix<double> A(as<MappedSparseMatrix<double> >(AA));
const MapVecd                    y(as<MapVecd>(yy));
const SparseMatrix<double>       At(A.adjoint());
return List::create(Named("At")  = At,
                    Named("Aty") = At * y);
```

Figure 14: `sparseProdCpp`: Transpose and product with sparse matrices.

```
typedef Eigen::MappedSparseMatrix<double>  MSpMat;
typedef Eigen::SparseMatrix<double>         SpMat;
typedef Eigen::SimplicialLDLT<SpMat>       SpChol;

const SpMat      At(as<MSpMat>(AA).adjoint());
const VectorXd  Aty(At * as<MapVecd>(yy));
const SpChol     Ch(At * At.adjoint());
if (Ch.info() != Eigen::Success) return R_NilValue;
return List::create(Named("betahat")  = Ch.solve(Aty),
                    Named("perm")     = Ch.permutationP().indices());
```

Figure 15: `sparseLSCpp`: Solving a sparse least squares problem.

Sparse Cholesky decompositions are provided by the `SimplicialLLT` and `SimplicialLDLT` classes in the **RcppEigen** package for R. These are subclasses of the `SimplicialCholesky` templated. A sample usage is shown in Figure 15.

```
R> sparse2 <- cxxfunction(signature(AA = "dgCMatrix", yy = "numeric"),
+    sparseLSCpp, "RcppEigen", incl)
R> str(rr <-  sparse2(KNex$mm, KNex$y))
```

```
List of 2
 $ betahat: num [1:712] 823 340 473 349 188 ...
 $ perm   : int [1:712] 572 410 414 580 420 425 417 426 431 445 ...
```

```
R> res <- as.vector(solve(Ch <- Cholesky(crossprod(KNex$mm)),
+    crossprod(KNex$mm, KNex$y)))
R> stopifnot(all.equal(rr$betahat, res))
R> all(rr$perm == Ch@perm)
```

```
[1] FALSE
```

The fill-reducing permutations are different.

7. Summary

This paper introduced the **RcppEigen** package which provides high-level linear algebra computations as an extension to the R system. **RcppEigen** is based on the modern C++ library **Eigen** which combines extended functionality with excellent performance, and utilizes **Rcpp** to interface R with C++. Several illustrations covered common matrix operations and several approaches to solving a least squares problem—including an extended discussion of rank-revealing approaches. Sparse matrix computations were also discussed, and a short example provided an empirical demonstration of the excellent run-time performance of the **RcppEigen** package.

References

Abrahams D, Gurtovoy A (2004). *C++ Template Metaprogramming: Concepts, Tools and Techniques from Boost and Beyond*. Addison-Wesley, Boston.

Bates D, Eddelbuettel D (2013). "Fast and Elegant Numerical Linear Algebra Using the RcppEigen Package." *Journal of Statistical Software*, **52**(5), 1–24. URL http://www.jstatsoft.org/v52/i05/.

Bates D, Maechler M (2014). ***Matrix**: Sparse and Dense Matrix Classes and Methods*. R package version 1.1-2, URL http://CRAN.R-Project.org/package=Matrix.

Eddelbuettel D (2013). *Seamless R and C++ Integration with Rcpp*. Use R! Springer, New York. ISBN 978-1-4614-6867-7.

Eddelbuettel D, François R (2011). "Rcpp: Seamless R and C++ Integration." *Journal of Statistical Software*, **40**(8), 1–18. URL http://www.jstatsoft.org/v40/i08/.

Eddelbuettel D, François R, Allaire J, Chambers J, Bates D, Ushey K (2014). *Rcpp: Seamless R and C++ Integration*. R package version 0.11.0, URL http://CRAN.R-Project.org/package=Rcpp.

François R, Eddelbuettel D (2012). *RcppGSL: Rcpp integration for GNU GSL vectors and matrices*. R package version 0.2.0, URL http://CRAN.R-Project.org/package=RcppGSL.

François R, Eddelbuettel D, Bates D (2014). *RcppArmadillo: Rcpp integration for Armadillo templated linear algebra library*. R package version 0.4.000.2, URL http://CRAN.R-Project.org/package=RcppArmadillo.

Guennebaud G, Jacob B, *et al.* (2012). "Eigen v3." URL http://eigen.tuxfamily.org.

ISO/IEC (2011). "C++ 2011 Standard Document 14882:2011." ISO/IEC Standard Group for Information Technology / Programming Languages / C++. URL http://www.iso.org/iso/iso_catalogue/catalogue_tc/catalogue_detail.htm?csnumber=50372.

Kusnierczyk W (2012). ***rbenchmark**: Benchmarking routine for R*. R package version 1.0.0, URL http://CRAN.R-Project.org/package=rbenchmark.

Meyers S (1995). *More Effective C++: 35 New Ways to Improve Your Programs and Designs*. Addison-Wesley, Boston. ISBN 020163371X.

Meyers S (2005). *Effective C++: 55 Specific Ways to Improve Your Programs and Designs*. Addison-Wesley, Boston, 3rd edition. ISBN 978-0321334879.

R Development Core Team (2012). *Writing R extensions*. R Foundation for Statistical Computing, Vienna, Austria. URL http://CRAN.R-Project.org/doc/manuals/R-exts.html.

R Development Core Team (2013). *R: A Language and Environment for Statistical Computing*. R Foundation for Statistical Computing, Vienna, Austria. URL http://www.R-project.org/.

Sklyar O, Murdoch D, Smith M, Eddelbuettel D, François R (2013). *inline: Inline C, C++, Fortran function calls from R.* R package version 0.3.13, URL `http://CRAN.R-Project.org/package=inline`.

Veldhuizen TL (1998). "Arrays in Blitz++." In "ISCOPE '98: Proceedings of the Second International Symposium on Computing in Object-Oriented Parallel Environments," pp. 223–230. Springer-Verlag, London. ISBN 3-540-65387-2.

Affiliation:

Douglas Bates
Department of Statistics
University of Wisconsin-Madison
Madison, WI, United States of America
E-mail: `bates@stat.wisc.edu`
URL: `http://www.stat.wisc.edu/~bates/`

Dirk Eddelbuettel
Debian Project
River Forest, IL, United States of America
E-mail: `edd@debian.org`
URL: `http://dirk.eddelbuettel.com`
