# Supplementary material for: The relationship between leaf area growth and biomass accumulation in Arabidopsis thaliana
Source: Front Plant Sci. 2015 Apr 9;6:167. doi: 10.3389/fpls.2015.00167 (PMC4391269; doi:10.3389/fpls.2015.00167)
Supplement: Supplementary file 4 [file DataSheet1.ZIP › R_libs - parallization.zip!/R_libs/RcppEigen/html/00Index.html]

R: Rcpp integration for the Eigen templated linear algebra library.

# Rcpp integration for the Eigen templated linear algebra library.

---

## Documentation for package ‘RcppEigen’ version 0.3.2.2.0

- DESCRIPTION file.
- User guides, package vignettes and other documentation.
- Package NEWS.

## Help Pages

|  |  |
| --- | --- |
| RcppEigen-package | Rcpp/Eigen bridge |
| fastLm | Bare-bones linear model fitting function |
| fastLm.default | Bare-bones linear model fitting function |
| fastLm.formula | Bare-bones linear model fitting function |
| fastLmPure | Bare-bones linear model fitting function |
| RcppEigen | Rcpp/Eigen bridge |
| RcppEigen.package.skeleton | Create a skeleton for a new package that intends to use RcppEigen |
