# Supplementary material for: The relationship between leaf area growth and biomass accumulation in Arabidopsis thaliana
Source: Front Plant Sci. 2015 Apr 9;6:167. doi: 10.3389/fpls.2015.00167 (PMC4391269; doi:10.3389/fpls.2015.00167)
Supplement: Supplementary file 4 [file DataSheet1.ZIP › R_libs - parallization.zip!/R_libs/RCppODE/html/00Index.html]

R: What the package does (short line)

# What the package does (short line)

---

## Documentation for package ‘RCppODE’ version 1.0

- DESCRIPTION file.

## Help Pages

|  |  |
| --- | --- |
| RCppODE-package | What the package does (short line) |
| RCppODE | What the package does (short line) |
| rcpp\_hello\_world | Simple function using Rcpp |
