# Supplementary material for: The relationship between leaf area growth and biomass accumulation in Arabidopsis thaliana
Source: Front Plant Sci. 2015 Apr 9;6:167. doi: 10.3389/fpls.2015.00167 (PMC4391269; doi:10.3389/fpls.2015.00167)
Supplement: Supplementary file 4 [file DataSheet1.ZIP › R_libs - parallization.zip!/R_libs/RCppODE/tests/HIRES.pdf]

```
#include <array>
#include <iostream>
#include <map>
using namespace std;

extern "C" {
  array<NamedVector<double>, 2> HIRES_CPP(const double& t,
                              const NamedVector<double>& states,
                              const NamedVector<double>& rparms,
                              const NamedVector<double>& forcings,
                              const NamedVector<double>& derived,
                              map<string, double>& rextra,
                              map<string, int>& iextra) {
 // Prepare output
  array<NamedVector<double>, 2> dydt {states, derived};
  // Get inputs
  const double y1 = states["y1"];
  const double y2 = states["y2"];
  const double y3 = states["y3"];
  const double y4 = states["y4"];
  const double y5 = states["y5"];
  const double y6 = states["y6"];
  const double y7 = states["y7"];
  const double y8 = states["y8"];
  const double p1 = rparms["p1"];
  const double p2 = rparms["p2"];
  const double p3 = rparms["p3"];
  const double p4 = rparms["p4"];
  const double p5 = rparms["p5"];
  const double p6 = rparms["p6"];
  const double p7 = rparms["p7"];
  const double p8 = rparms["p8"];
  const double p9 = rparms["p9"];
  const double p10 = rparms["p10"];
  const double p11 = rparms["p11"];
  const double p12 = rparms["p12"];
  const double p13 = rparms["p13"];
  const double p14 =rparms["p14"];
  // Output calculations
  dydt[0]["y1"]  = p1*y1 + p2*y2 + p3*y3 + p4 + forcings["dummy"];
  dydt[0]["y2"]  = -p1*y1 - p5*y2;
  dydt[0]["y3"]  = -p6*y3 + p7*y4 + p8*y5;
  dydt[0]["y4"]  = p9*y2 - p1*y3 - p10*y4;
  dydt[0]["y5"]  = -p11*y5 + p7*y6 + p7*y7;
  dydt[0]["y6"]  = -p12*y6*y8 + p13*y4 - p1*y5 - p7*y6 + p13*y7;
  dydt[0]["y7"]  = p12*y6*y8 - p14*y7;
  dydt[0]["y8"]  = -p12*y6*y8 + p14*y7;
  dydt[1]["balance"] = dydt[0][0] + dydt[0][1] + dydt[0][2] + dydt[0][3] +
      dydt[0][4] + dydt[0][5] +
                           dydt[0][6] + dydt[0][7];
  return(dydt);
```

```
    };
}
```
