# Supplementary material for: The relationship between leaf area growth and biomass accumulation in Arabidopsis thaliana
Source: Front Plant Sci. 2015 Apr 9;6:167. doi: 10.3389/fpls.2015.00167 (PMC4391269; doi:10.3389/fpls.2015.00167)
Supplement: Supplementary file 4 [file DataSheet1.ZIP › R_libs - parallization.zip!/R_libs/RCppODE/tests/Rplots.pdf]

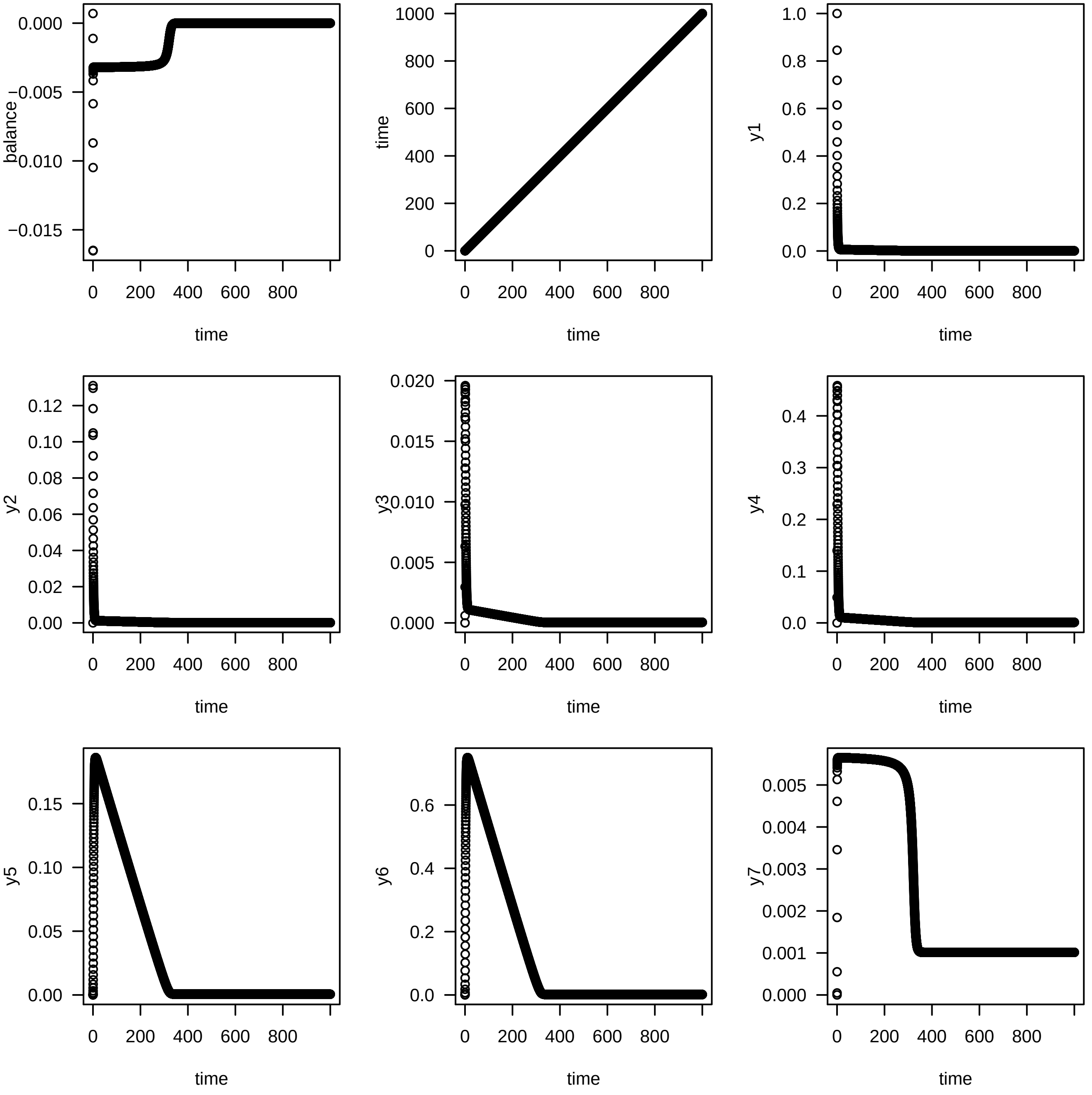

balance
time
time
time
y1
time
y2
time
y3
time
y4
time
y5
time
y6
time
y7
time

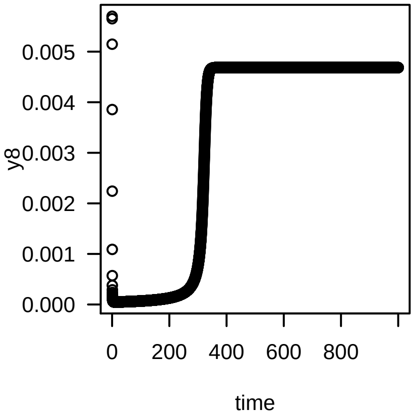

0.005
0.004
0.003
0.002
0.001
0.000
y8
0
200
400
600
800
time
