# Supplementary material for: The relationship between leaf area growth and biomass accumulation in Arabidopsis thaliana
Source: Front Plant Sci. 2015 Apr 9;6:167. doi: 10.3389/fpls.2015.00167 (PMC4391269; doi:10.3389/fpls.2015.00167)
Supplement: Supplementary file 4 [file DataSheet1.ZIP › R_libs - parallization.zip!/R_libs/rngWELL/html/00Index.html]

R: toolbox for WELL random number generators.

# toolbox for WELL random number generators.

---

## Documentation for package ‘rngWELL’ version 0.10-3

- DESCRIPTION file.
- Package NEWS.

## Help Pages

|  |  |
| --- | --- |
| dogetRngWELL | Toolbox for pseudo and quasi random number generation |
| doinitMT2002 | Toolbox for pseudo and quasi random number generation |
| doputRngWELL | Toolbox for pseudo and quasi random number generation |
| pseudo.rngWELL | Toolbox for pseudo and quasi random number generation |
| setSeed4WELL | Toolbox for pseudo and quasi random number generation |
| WELL2test | Toolbox for pseudo and quasi random number generation |
